# Sustainable dye removal from industrial wastewater using marine algae-derived biosorbents and MOF-based hybrid composites

**DOI:** 10.1038/s41598-026-41983-5

**Published:** 2026-03-29

**Authors:** Suzan A. R. Abdel‑Razik, Mohamed S. Abdel‑Kareem, Nagwa I. El‑Agawany, Bassma M. Ali, Mona I.A. Kaamoush

**Affiliations:** 1 Botany and Microbiology Department, Faculty of Science, Alexandria University, Alexandria, Egypt; 2 College of Pharmacy, Arab Academy for Science, Technology and Maritime Transport (AAST), Abu-Qir, Alexandria, Egypt; 3 Environmental Protection and Crises Management Department, Simulators Complex, Arab Academy for Science, Technology and Maritime Transport (AAST), Alexandria, Egypt

**Keywords:** Marine algae, Metal-organic frameworks, Dye removal, Biosorption, Wastewater treatment, Adsorption kinetics, Sustainable remediation, Textile effluents, Chemistry, Environmental sciences, Water resources

## Abstract

This study presents a comparative investigation of marine algal biomasses (fresh and dried) and a zirconium-based metal–organic framework (UiO-66-NH₂) for the removal of reactive dyes from industrial wastewater. Three widely used textile dyes Reactive Yellow 2 (RY2), Reactive Red 195 (RR195), and Reactive Blue 19 (RB19) were selected as model pollutants. Adsorption experiments were conducted under unified experimental conditions to evaluate the effects of initial dye concentration, solution pH, adsorbent dosage, and contact time. Control experiments at acidic pH confirmed that dye removal occurred predominantly via adsorption rather than precipitation. Adsorption kinetics and equilibrium behaviour were analysed using kinetic models and Langmuir and Freundlich isotherms. The results demonstrated that dried algal biomass exhibited significantly higher removal efficiencies compared to fresh biomass, reaching up to 96% dye removal, due to enhanced surface area and availability of active functional groups. UiO-66-NH₂ showed high adsorption capacity and stability, particularly under acidic conditions, owing to strong electrostatic interactions, hydrogen bonding, and π–π stacking. Comparative analysis highlights the advantages and limitations of low-cost marine algal biosorbents relative to advance MOF materials. Overall, the findings provide valuable insight into sustainable, efficient, and scalable strategies for the treatment of dye-contaminated industrial wastewater.

**Supplementary Information:**

The online version contains supplementary material available at 10.1038/s41598-026-41983-5.

## Introduction

The discharge of dye-containing wastewater from textile industries into aquatic environments represents a serious environmental and public health concern due to the toxic, mutagenic, and potentially carcinogenic nature of many synthetic dyes^1^. Reactive dyes, including Reactive Blue 19 (RB19), Reactive Red 195 (RR195), and Reactive Yellow 2 (RY2), are among the most extensively used dyes in textile processing because of their high color fastness, water solubility, and strong covalent bonding with fibers^2^. However, these same properties render them highly resistant to conventional wastewater treatment processes. Even at low concentrations (< 1 mg L⁻¹), reactive dyes can significantly impair aquatic ecosystems by reducing light penetration, inhibiting photosynthesis, and disturbing ecological balance. The World Health Organization recommends a maximum permissible concentration of 1 mg L⁻¹ for coloring substances in drinking water, emphasizing the urgent need for efficient dye removal technologies. Globally, textile activities are estimated to contribute approximately 17–20% of industrial water pollution, with dye-laden effluents representing one of the most persistent contamination sources^3,4^.

Conventional treatment methods such as coagulation–flocculation, electrochemical oxidation, membrane filtration, and advanced oxidation processes have been widely applied for dye removal; however, these approaches often suffer from high operational costs, secondary pollution, and limited efficiency under variable wastewater compositions^5,6^. Consequently, adsorption-based techniques have gained increasing attention as cost-effective and environmentally benign alternatives.

Marine algal biomasses have emerged as promising biosorbents due to their natural abundance, low cost, biodegradability, and the presence of diverse surface functional groups such as hydroxyl, carboxyl, sulfate, and amine groups capable of binding dye molecules through electrostatic interactions, ion exchange, and complexation mechanisms^7–11^. Both live and dried algal biomasses have demonstrated potential for dye removal, with dried forms often exhibiting enhanced adsorption capacity due to increased surface area and accessibility of binding sites^12–14^.

In parallel, metal–organic frameworks (MOFs), particularly zirconium-based materials such as UiO-66-NH₂, have attracted considerable interest for adsorption applications owing to their exceptionally high surface area, tunable pore structure, and chemical stability in aqueous environments^15,16^. The presence of amine functional groups in UiO-66-NH₂ further enhances its affinity toward anionic dyes through hydrogen bonding and electrostatic interactions.

Despite the extensive literature on algal biosorbents and MOFs as individual adsorbents, a clear research gap remains. Few studies have conducted a systematic and direct comparison between live algal biomass, dried algal biomass, and MOF-based adsorbents for the removal of identical reactive dyes under identical experimental conditions. Such comparative evaluations are essential to objectively assess performance, sustainability, and practical applicability.

### Novelty and aim

The novelty of the present study lies in providing a comprehensive comparative assessment of live and dried marine macroalgae (*Ulva fasciata* and *Pterocladia capillacea*) and a zirconium-based metal–organic framework (UiO-66-NH₂) for the removal of the same reactive dyes (RY2, RR195, and RB19) under unified experimental conditions. The study aims to (i) evaluate and compare adsorption performance across different adsorbent systems, (ii) elucidate adsorption mechanisms using kinetic and isotherm models, and (iii) assess the practical potential of low-cost biosorbents relative to advanced MOF materials for sustainable wastewater treatment.

In this work, the effects of key operational parameters including initial dye concentration, solution pH, adsorbent dosage, contact time, and reusability were systematically investigated. Dye concentration ranges were selected to reflect those commonly encountered in textile effluents. Additionally, real industrial wastewater was examined to validate the applicability of the studied adsorbents under realistic conditions. The findings of this study contribute to advancing sustainable and scalable strategies for dye-contaminated wastewater treatment, aligning with the goals of environmentally responsible water management and circular economy principles.

## Materials and methods

### Biomaterials

According to El-Agawany et al.^17^, two marine macroalgae, *Ulva fasciata* and *Pterocladia capillacea*, were selected as biosorbents due to their demonstrated efficiency in reactive dye removal in both fresh and dried forms. Algal samples were collected from the Mediterranean Sea shoreline at Abu-Qir, Alexandria, Egypt. The collected biomass was thoroughly washed with filtered seawater to remove sand and epiphytes. Part of the biomass was preserved in seawater and used as fresh (live) algae, while the remaining samples were oven-dried at 60 °C, ground into a fine powder, and stored in airtight containers for further experiments.

### Chemical materials

Zirconium (IV) chloride (ZrCl₄), 2-aminoterephthalic acid, N, N-dimethylformamide (DMF), and ethanol used for the synthesis of UiO-66-NH₂ were of analytical grade and purchased from Sigma-Aldrich. Double-distilled water was used throughout all experimental procedures. UiO-66-NH₂ was synthesized via a solvothermal method using zirconium (IV) chloride and 2-aminoterephthalic acid in DMF. The synthesized MOF was washed, activated, and dried prior to use. Structural characterization was conducted to confirm MOF formation.

### Reactive dyes

Three commercial reactive dyes supplied by Misr El-Beida Dyers S.A.E. (Kafr El-Dawar, El-Beheira, Egypt) were used as model pollutants: Reactive Yellow 2 (RY2; λmax = 404 nm), Reactive Red 195 (RR195; λmax = 540 nm), and Reactive Blue 19 (RB19; λmax = 594 nm). Dye stock solutions (1000 mg L⁻¹) were prepared by dissolving 100 mg of dye powder in 100 mL of filtered seawater, followed by appropriate dilution to obtain the required concentrations. Maximum absorbance wavelengths were determined using a PerkinElmer Lambda 4B dual-beam UV–Vis spectrophotometer.

### Batch adsorption experiments

Batch adsorption experiments were performed to evaluate the effects of initial dye concentration, solution pH, adsorbent type (live algae, dried algae, UiO-66-NH₂, and algae–MOF hybrid), dosage, and contact time on dye removal efficiency. Dye concentrations were quantified using UV–Vis spectrophotometry.

### Statistical analysis

Statistical analysis was conducted using the Kruskal–Wallis one-way ANOVA, followed by Dunn’s post hoc test. Effect size (η²) was calculated to evaluate the magnitude of differences among treatments.

Statistical analysis of the adsorption results was conducted using the Kruskal–Wallis one-way ANOVA, with effect size (η²) calculated and Dunn’s post hoc test applied to identify significant differences among treatments.

The chemical materials in this study were mainly three commercial-grade reactive dyes (Misr El Beida Dyers S.A.E., Kafr El Dawar, El Beheira, Egypt) which are:

- (RY2) Reactive Yellow 2, **M.F.** C 25 H 15 Cl 3 N 9 Na 3O10S3, λ_max_ = 404 nm.
- (RR195) Reactive Red 195, M.F. C₃₁H₁₉ClN₇O₁₉S₆. ₅Na₅, λ max = 540 nm.
- (RB19) Reactive Blue 19. **M.F**. C_22_H_16_N_2_Na_2_O_11_S_3 **-**_ λ_max_ = 594 nm.

Each dye’s maximum wavelength was determined using a Perkin-Elmer Lambda 4B dual-beam UV-visible spectrophotometer. One hundred milliliters of filtered saltwater was used to dissolve one hundred milligrams of pure powder to create a stock dye solution. Diluting the stock solution allowed for the preparation of several dye concentrations.Fresh marine algae were washed thoroughly with distilled water. **Live algae** were used immediately after preparation, while **dried algae** were oven-dried at controlled temperature and ground to a uniform particle size.

### Synthesis of MOF

The metal–organic framework UiO-66-NH₂ used in this study was synthesized via a solvothermal method. Zirconium (IV) chloride and 2-aminoterephthalic acid were dissolved in N, N-dimethylformamide (DMF) under continuous stirring until a homogeneous solution was obtained. The mixture was transferred to a Teflon-lined autoclave and heated at 120 °C for 24 h. After cooling, the resulting precipitate was collected by centrifugation, washed repeatedly with DMF and ethanol to remove unreacted species, and dried at 60 °C. The synthesized UiO-66-NH₂ was stored in a desiccator for further use^18^.

### Application of algal biomass and MOF in combined adsorption experiments

Algal biomass and UiO-66-NH₂ were not chemically integrated during synthesis. Instead, both materials were applied either individually or simultaneously during batch adsorption experiments to evaluate their individual and combined adsorption performance. For combined adsorption experiments, predetermined amounts of algal biomass and UiO-66-NH₂ were added together into the dye solution and agitated under controlled conditions. The interaction between algal functional groups and the MOF surface occurred during the adsorption process, resulting in enhanced dye removal efficiency without the formation of a chemically bonded hybrid composite.

### Application of algal biomass and MOF during adsorption experiments

Algal biomass and UiO-66-NH₂ were not chemically integrated or immobilized during material synthesis. Instead, each adsorbent was applied independently in batch adsorption experiments to evaluate its performance toward the removal of reactive dyes. In selected experiments, algal biomass and UiO-66-NH₂ were added simultaneously to the dye solution to assess their combined effect under identical operating conditions. Any interaction between algal functional groups and the MOF surface occurred physically during the adsorption process, without the formation of a chemically bonded hybrid composite.

### Effect of different factors on the biosorption process

All of the experiments were carried out at room temperature using the algal biomass as the biosorbent. Determined algal biomass (fresh or dried) was used in 100 ml of the appropriate dye solution in a 250 ml pre-sterile Erlenmeyer flask. The absorbance of each dye was measured (according to its λ_max_) by a dual-beam UV-visible spectrophotometer (Perkin–Elmer Lambda 4B) during the experiment in certain time intervals (t). Various algal biomasses were implemented to see how they affected the decolorization procedure. 1 to 5 g of fresh biomass, and 0.5 to 2.5 g of dried biomass of both studied algal species.

In order to investigate the effect of each factor distinctly, we changed this factor only and fixed all other factors.

### Effect of initial dye concentration

The following dye concentrations used to investigate the influence of initial dye concentration on biosorption efficiency:

a- **RY2**: 30, 60, 90, 120, and 150 mg/l.

b- **RR195**: 10, 20, 30, 40 and 50 mg/l.

c- **RB19**: 20, 40, 60, 80 and 100 mg/l.

All other factors were constant. The algal biomass (0.5 g of dried biomass or 2 g of fresh biomass), the salinity (38%), and the pH (8). At 2, 4, 6, and 8 h. intervals, the absorbance of each dye was measured.

### Effect of different pHs

Several pH solutions were created from natural seawater to determine how much acidity and alkalinity impact algal biosorption capabilities (pH 2–10). 1 N HCl was used to make acidic solutions (pH 2, 4, and 6), while 1 N NaOH was used to make alkaline solutions (pH 10). All other factors were constant, as mentioned above. The absorbance of each dye was measured spectrophotometrically after certain time intervals (t).

Blank control experiments were conducted at pH 2 using dye solutions without adsorbents to confirm that dye precipitation did not occur under acidic conditions. No significant color reduction was observed in control samples, confirming that removal was due to adsorption rather than precipitation.

Batch adsorption studies investigated the effects of pH, initial dye concentration, and contact time. **Blank control experiments** (without adsorbent) were conducted at acidic pH to exclude dye precipitation effects.

#### Graphical adsorption mechanism

**Fig. 1:**
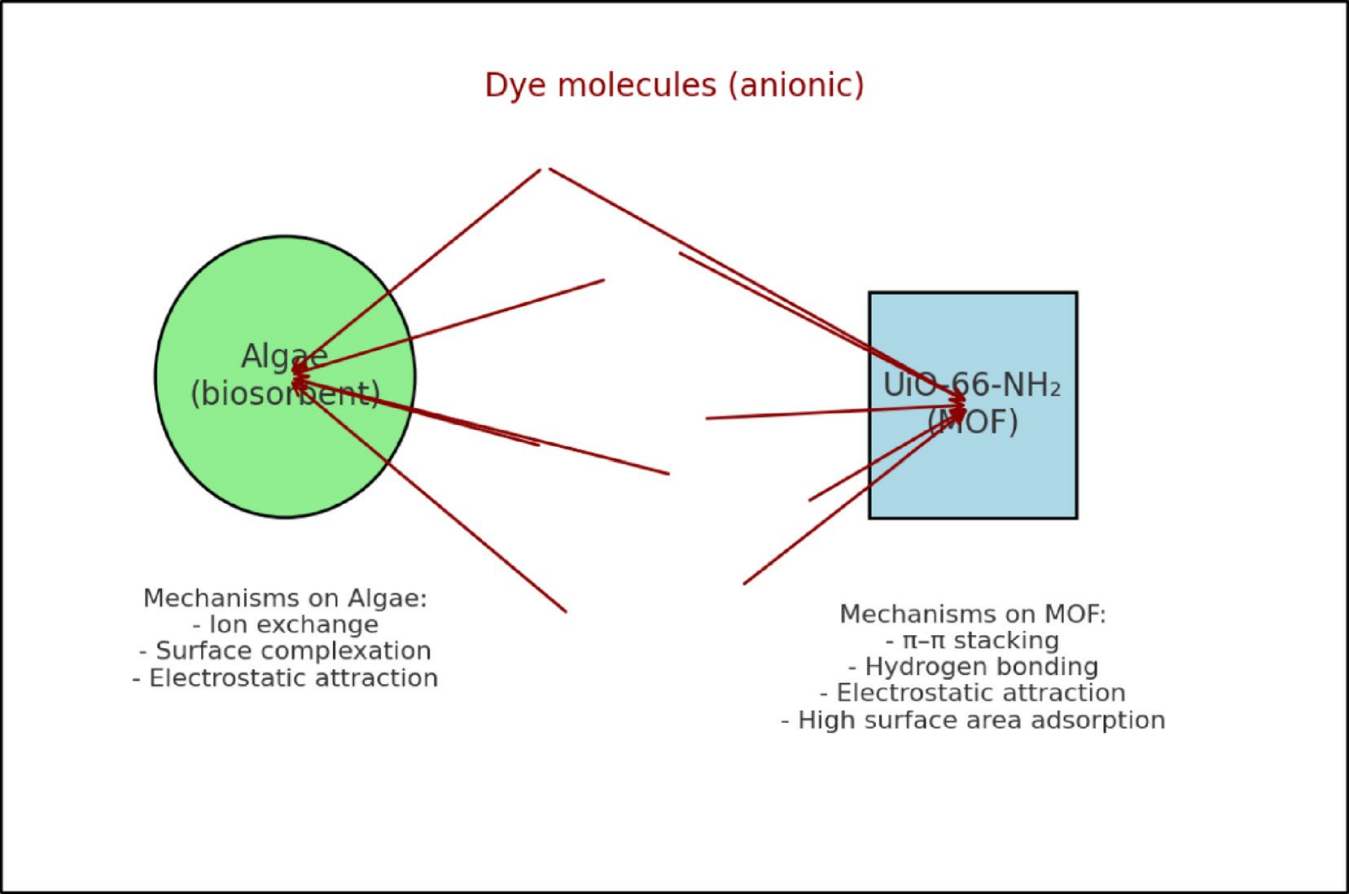
Schematic illustration of adsorption mechanisms for algae and UiO-66-NH₂, showing electrostatic interaction, hydrogen bonding, and π-π stacking.

The adsorption of reactive dyes onto marine algal biomass (Ulva fasciata and Pterocladia capillacea) and UiO-66-NH₂ occurs via distinct physicochemical interactions that enable efficient dye removal. For the algal biomass, electrostatic attraction between the negatively charged sulfonate groups of the dyes and the positively charged functional groups (such as –NH₂ and –OH) on the cell walls under acidic conditions (pH 2) is the primary mechanism. Hydrogen bonding involving –OH, –NH₂, and –COOH groups also stabilizes dye molecules on the algal surface. In the case of UiO-66-NH₂, electrostatic interactions similarly play a role, while π–π stacking between the aromatic rings of dye molecules and the conjugated organic linkers of the MOF enhances adsorption of dyes with aromatic structures. Thus, although the adsorption mechanisms differ slightly, both adsorbents individually exhibit high binding affinity and effective removal of reactive dyes from aqueous solutions. The proposed adsorption mechanisms for algal biomass and UiO-66-NH₂ are schematically illustrated in Fig. 1.

### Calculations and Statistical evaluation

The dye removal percentage was calculated by the following equation:

1 $$\mathrm{\%}ofDyeremoval=(1-\text{}C_t\text{}\text{}/C_o)\times100$$

*were*.

C_o_​: Initial dye concentration (mg/L) in the solution at the beginning of the experiment.

C_t_​: Dye concentration (mg/L) in the solution at a specific time t.

All biosorption investigations were done in triplicate, and the data was analyzed using the mean values. All results were presented in the form of means ± SD (standard deviations). Almost all trials show a statistically significant difference, as indicated by Kruskal-Wallis’s One Way Analysis of Variance on Ranks, which indicates that differences in the median values among the groups receiving treatment are often bigger than would be predicted by chance at *P* < 0.05.

## Result and discussion

The effect of using fresh versus dried algal biomass on the removal of three reactive dyes (RY2, RR195, and RB19) was systematically evaluated. Dye removal efficiency depended on factors such as dye–sorbent interactions, sorbent surface area, biomass dosage, and solution pH. The decolorization process primarily involved sorption and ion-exchange mechanisms^19^.

### Fresh algal biomass

As shown in Fig. 1a–c; Table 1, the removal percentages of RY2, RR195, and RB19 by fresh Ulva fasciata increased with increasing biomass and reached maximum values after 8 h. The highest removal efficiencies were obtained using 5 g of fresh biomass, corresponding to 84.86%, 79.98%, and 70.00% for RY2, RR195, and RB19, respectively. One-Way ANOVA confirmed that the increase in biomass significantly enhanced dye removal (*p* < 0.05), highlighting the critical role of biomass dosage.

A similar trend was observed for fresh Pterocladia capillacea (Fig. 1d–f; Table 2), with maximum removal efficiencies of 67.44% (RY2), 76.13% (RR195), and 64.23% (RB19) using 5 g of biomass after 8 h. These results indicate that prolonged contact time combined with higher biomass significantly improves biosorption efficiency (*p* < 0.05).

**Fig. 2:**
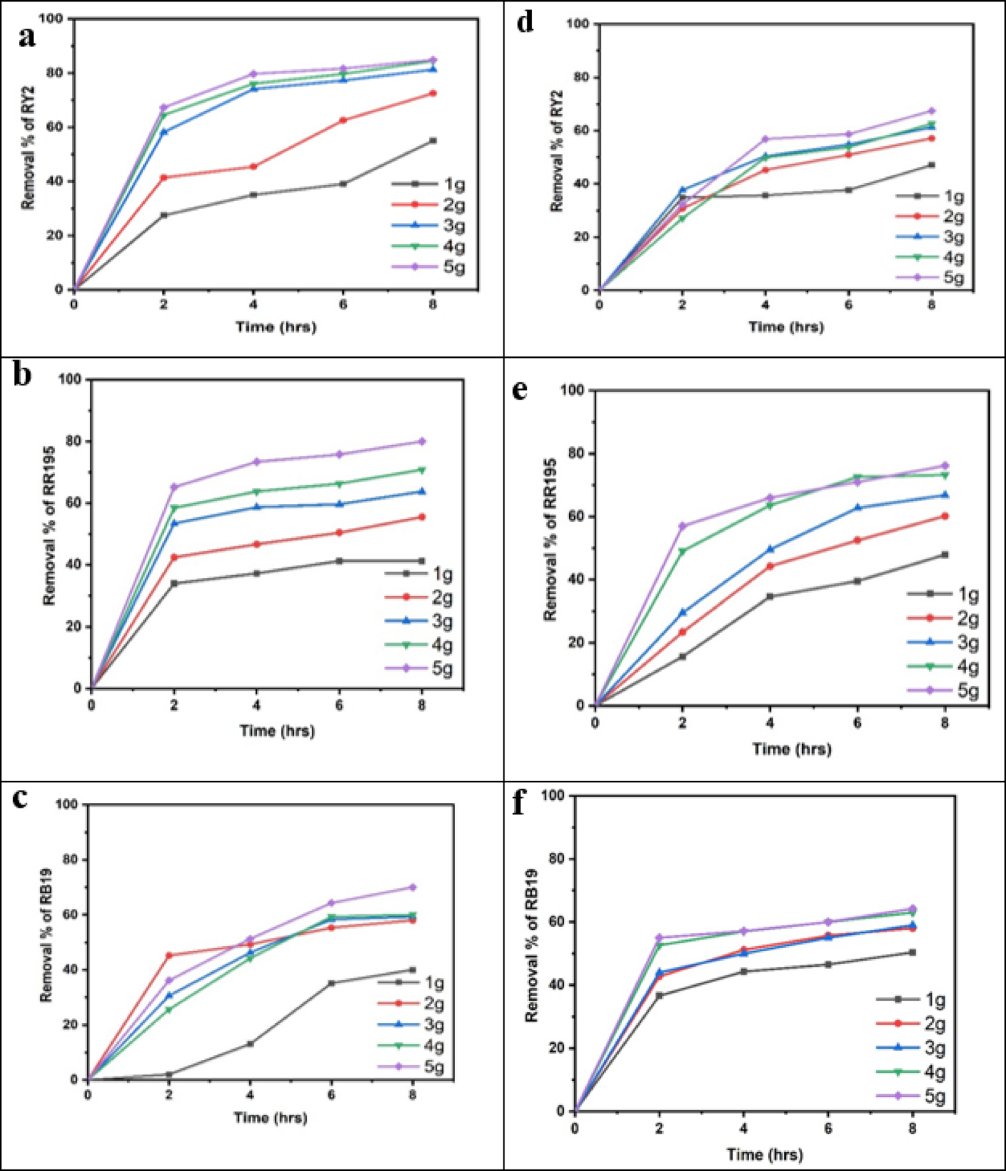
Effect of Different fresh algal biomass of both *Ulva fasciata* (**a**,** b**,**c**) and *Pterocladia capillacea* (**d**,** e**,**f**) on the removal of RY2, RR195, and RB19, respectively.

As illustrated in Fig. 2, increasing the fresh algal biomass of Ulva fasciata and Pterocladia capillacea significantly enhanced the removal efficiency of the three reactive dyes (RY2, RR195, and RB19), confirming the strong dependence of biosorption performance on biomass dosage.

### Dried algal biomass

**Fig. 3:**
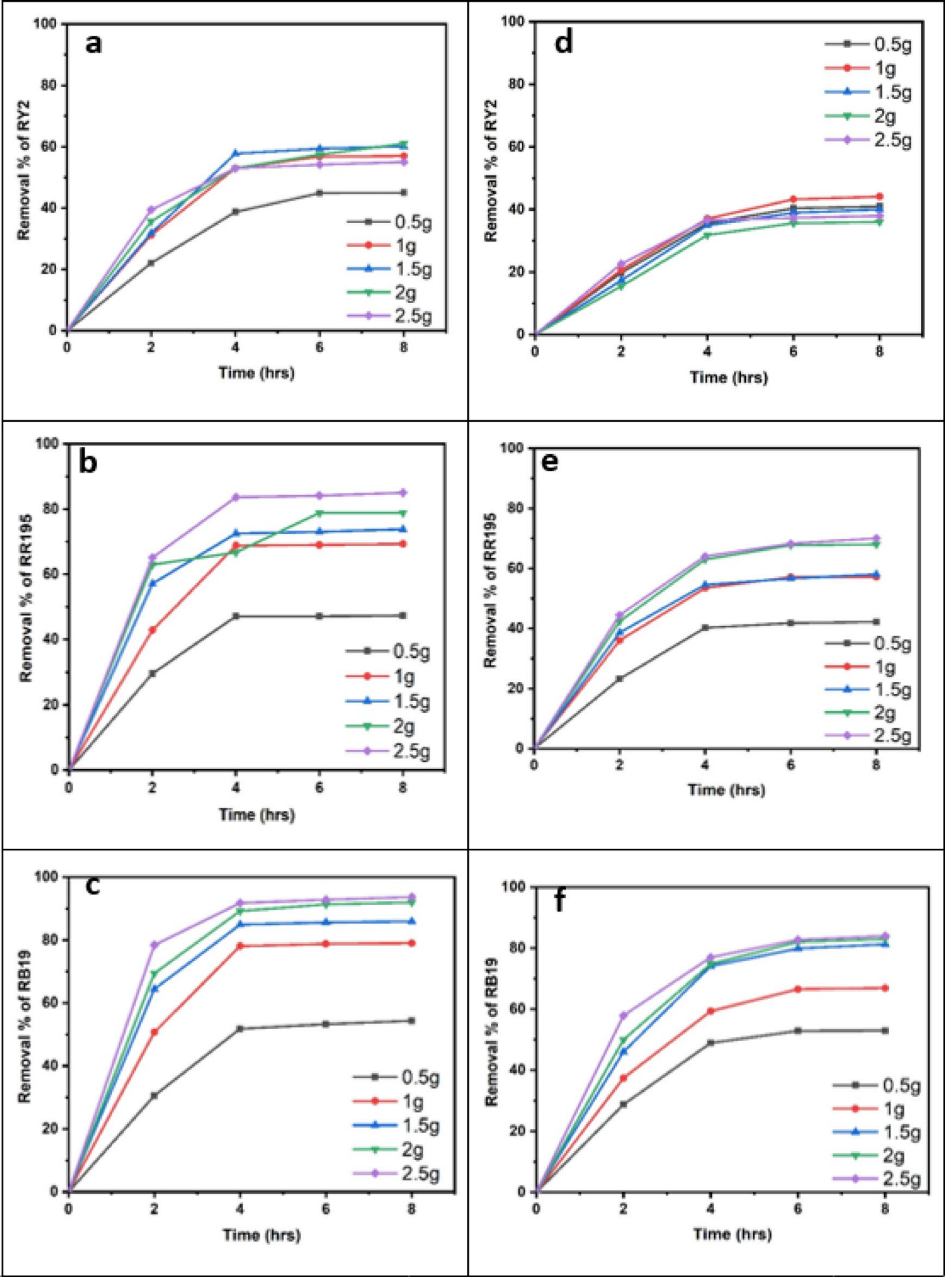
Effect of Different dried algal biomass of both *Ulva fasciata* (**a**,** b**,**c**) and *Pterocladia capillacea* (**d**,** e**,**f**) on the removal of RY2, RR195, and RB19, respectively.

Dried biomass exhibited superior dye uptake compared to fresh biomass, achieving maximum removal efficiencies of 96.31% (RY2), 93.83% (RR195), and 95.78% (RB19). This improvement is attributed to higher surface area and greater availability of functional groups upon drying, facilitating stronger dye–biomass interactions. As shown in Fig. 3, increasing the dried algal biomass significantly enhanced the removal efficiencies of RY2, RR195, and RB19.

### UiO-66-NH₂

Characterization analyses revealed that UiO-66-NH₂ possesses high crystallinity and porosity conducive to adsorption. The MOF displayed high adsorption capacity for all three dyes and maintained good reusability after multiple cycles. Acidic solution pH favored adsorption, consistent with electrostatic attraction between the negatively charged dye molecules and the positively charged amine groups of the MOF.

### Removal of dyes by algal biomass

The effect of using fresh versus dried algal biomass (Ulva fasciata and Pterocladia capillacea) on the removal of RY2, RR195, and RB19 was systematically investigated. For fresh biomass, dye removal increased with biomass dosage and contact time, reaching maximum efficiencies after 8 h using 5 g of biomass: 84.86%, 79.98%, and 70.00% for RY2, RR195, and RB19, respectively, for Ulva fasciata. Pterocladia capillacea showed slightly different efficiencies: 67.44%, 76.13%, and 64.23% for the same dyes. Dried biomass exhibited superior adsorption, achieving up to 96.31%, 93.83%, and 95.78% removal for RY2, RR195, and RB19, respectively. Characterization (FTIR, SEM, EDX) confirmed the presence of functional groups and porous structures facilitating dye binding via electrostatic interactions, hydrogen bonding, and complexation.

### Removal of dyes by UiO-66-NH₂

UiO-66-NH₂ independently exhibited high adsorption capacity for all tested dyes. Its high crystallinity and porosity, combined with amine functional groups, enabled strong electrostatic interactions with sulfonate groups of dyes, particularly under acidic conditions. The MOF maintained good reusability over multiple cycles, and characterization analyses (FTIR, SEM) confirmed dye adsorption on the MOF surface.

Several previous studies support our findings regarding the effect of algal biomass on dye removal. Omar et al.^20^ reported that the removal efficiency of Malachite Green increased with biomass weight up to 2.0 g, reaching maximum removal percentages of 97.8%, 98.3%, and 96.2% for Ulva reticulata, Sargassum crassifolium, and Gracilaria corticata, respectively. Similarly, Al-Fawwaz^21^ demonstrated that increasing biomass of two microalgal species (Dunaliella salina and Chlorella vulgaris) significantly enhanced decolorization of Methylene Blue and Malachite Green. Khataee et al.^22^ also observed that 4 g of Cladophora sp. achieved the highest Malachite Green removal, attributing the improvement to the greater surface area available for dye binding with higher biomass^23^.

### Adsorption Isotherms

Langmuir and Freundlich models were applied to equilibrium data, with the Langmuir model providing a superior fit (R² > 0.98), indicating monolayer adsorption. Maximum adsorption capacities (qₘ) were as follows: UiO-66-NH₂ – 127.4 mg/g (RY2), 118.6 mg/g (RR195), and 135.2 mg/g (RB19); dried U. fasciata – 89.3 mg/g (RY2), 76.8 mg/g (RR195), and 94.2 mg/g (RB19). These values are comparable with previously reported capacities for similar biosorbents.

### Dried vs. fresh algal biomass

Dried biomass consistently outperformed fresh biomass in dye removal. For dried U. fasciata, the highest biomass tested (2.5 g) achieved the maximum removal after 8 h, with RB19 reaching 93.7%, representing the best record for this alga. Conversely, the lowest biomass (0.5 g) showed the lowest removal percentages for all dyes.

For dried P. capillacea, results varied among dyes. The maximum removal of RY2 (44.11%) was achieved with 1 g of dried biomass, while the highest removal of RR195 and RB19 (70.0% and 84.0%, respectively) required 2.5 g of biomass. Statistical analysis confirmed significant differences in removal efficiencies among various biomass levels for both algae (*p* < 0.05). H-statistics ranged from 12.23 to 13.50, indicating that increased biomass consistently enhanced dye removal performance.

These findings validate the efficiency of dried U. fasciata and P. capillacea as potent natural biosorbents, with performance strongly influenced by biomass dosage and contact time.

**Table 1:**
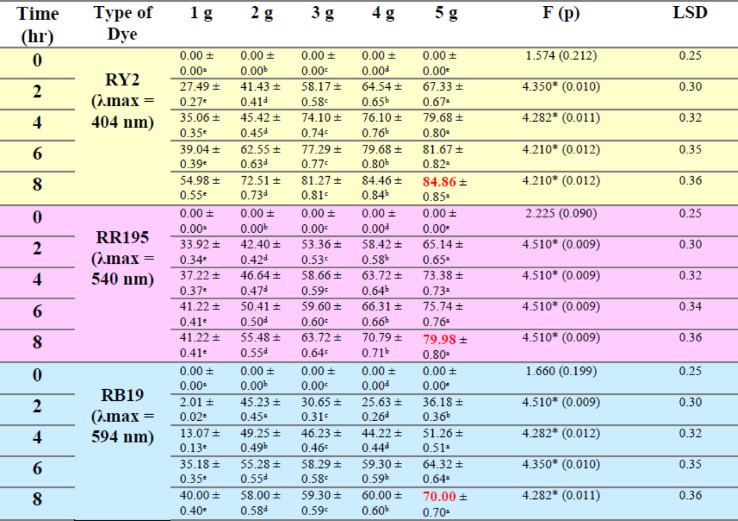
Effect of Different fresh algal biomass of *U. fasciata* on Removal % of three Reactive Dyes.

**Table 2:**
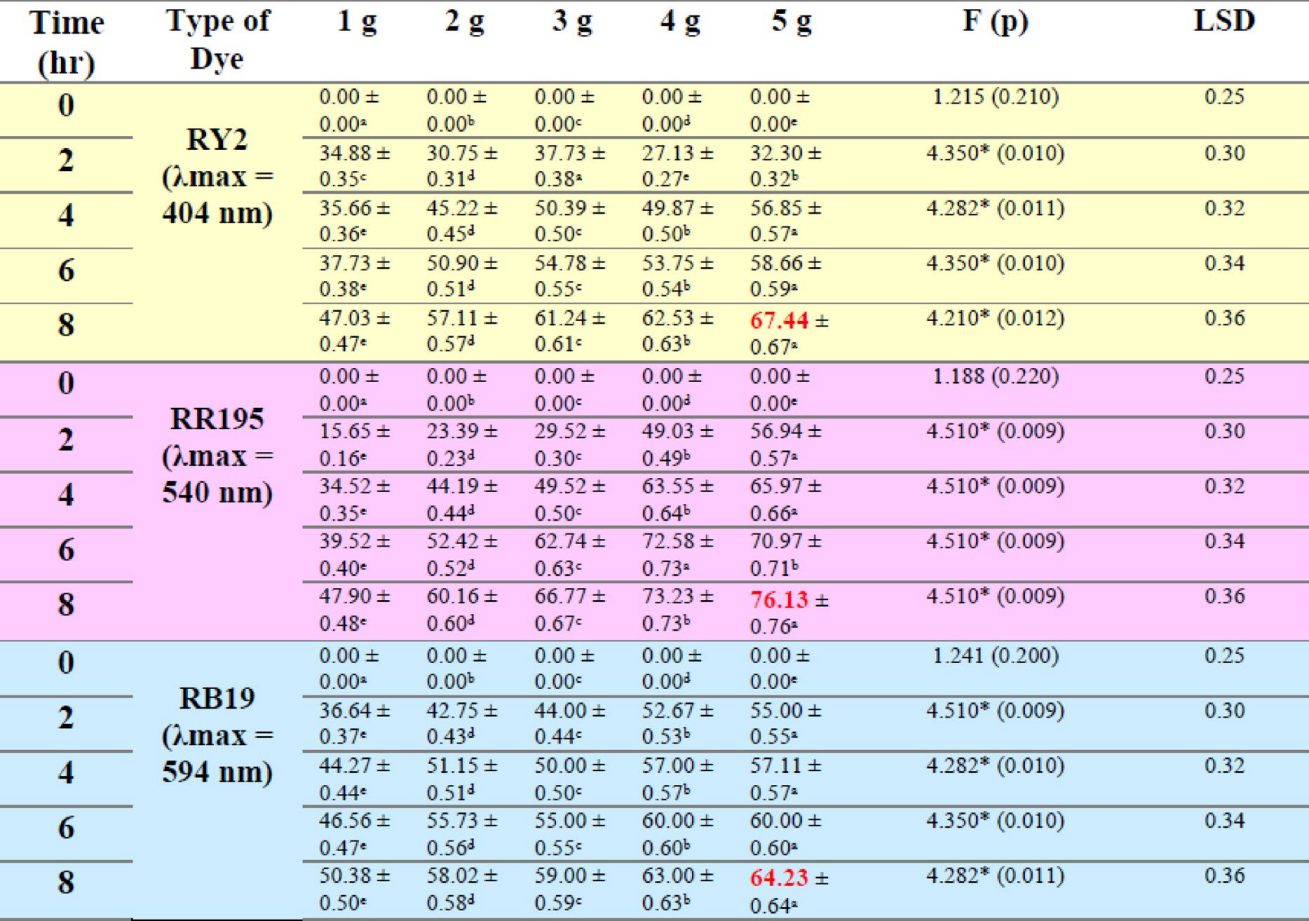
Effect of different fresh algal biomass of *Pterocladia capillacea* on removal % of three reactive dyes.

LSD: Least significant difference at 0.05. * : Statistically significant at *p* ≤ 0.05. ** : Statistically significant at *p* ≤ 0.01. Different subscripts are significant. Data are expressed in mean ± SD.

**Table 3:**
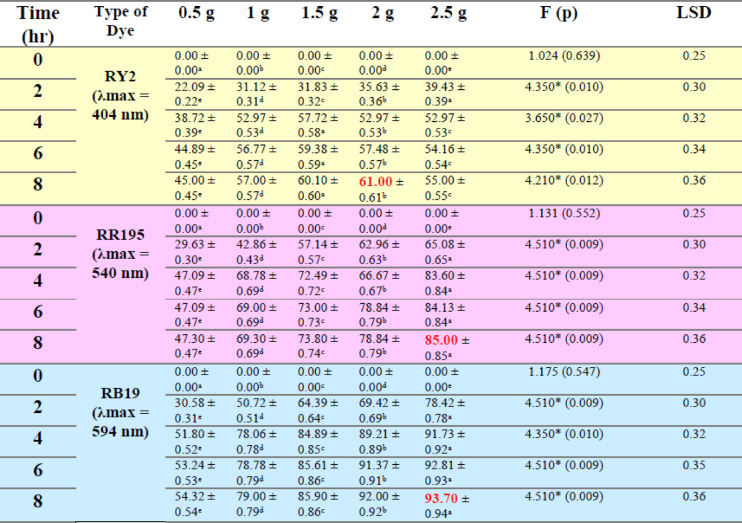
Effect of different dried algal biomass of *Ulva fasciata* on removal % of three reactive dyes.

**Table 4:**
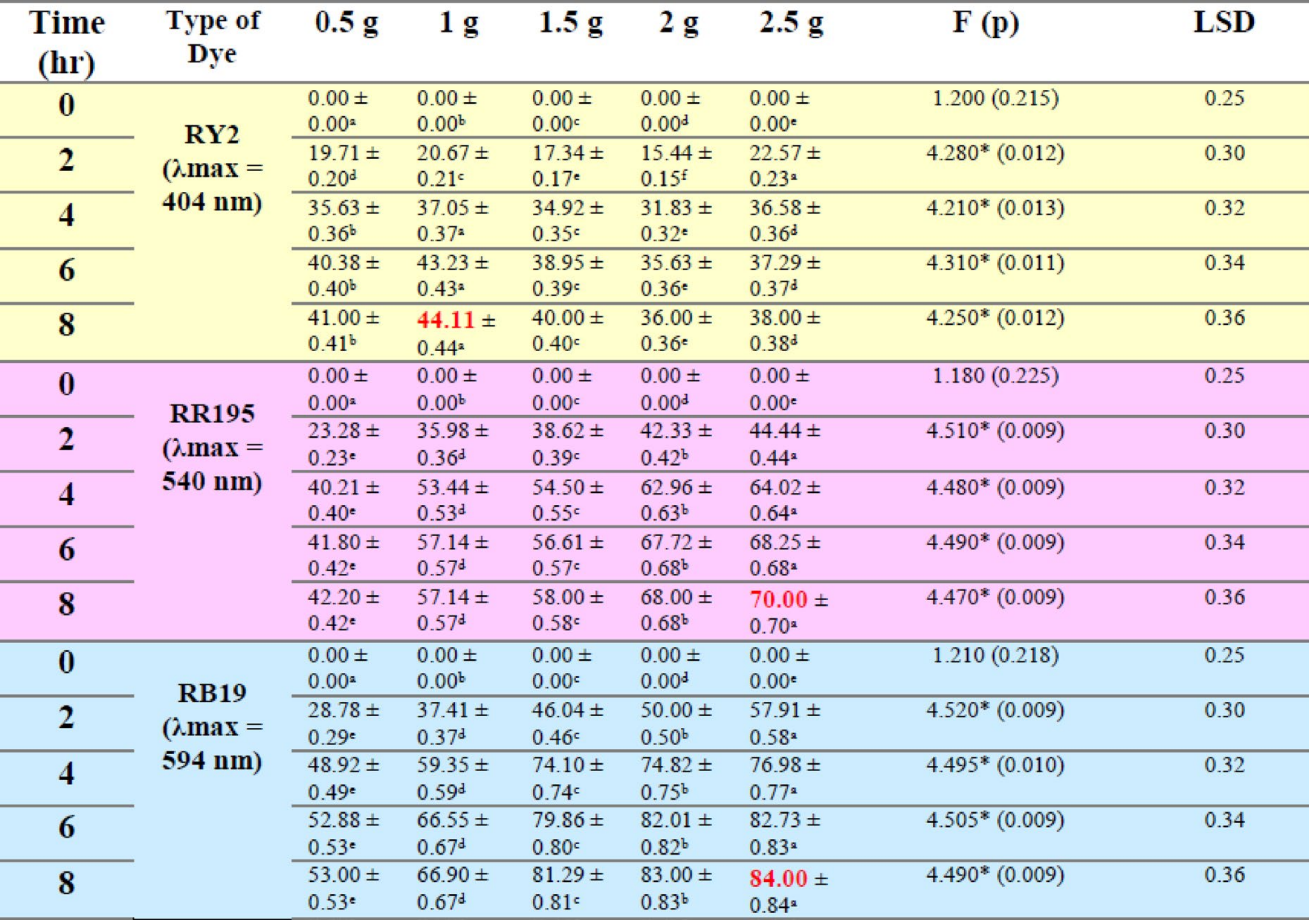
Effect of different dried algal biomass of *Pterocladia capillacea* on removal % of three reactive dyes.

LSD: Least significant difference at 0.05. * : Statistically significant at *p* ≤ 0.05. ** : Statistically significant at *p* ≤ 0.01. Different subscripts are significant. Data are expressed in mean ± SD.

Rorissa et al. (2022)^3^ mentioned that for color removal, non-living algae are superior to living algae. Our results also agreed with **Pathak** et al.^24^, who proved that dry algal biomass of *Chlorella pyrenoidosa* is a more efficient biosorbent of MB dye than fresh algal biomass and attributed this to the increasing of the algal surface area and strong binding affinities for the dye. Our mentioned results proved that *Ulva fasciata* and *P. capillacea* have acceptable removal abilities for the three reactive dyes under study, with optimal fresh biomass (5 g) and optimal dried biomass (2.5 g). This can be attributed to the increased surface area of the biosorbent, which increases the number of binding sites. In this regard, our findings were totally compatible with those of other prior authors^25,26^.

### Chemical functionalities by fourier transform infrared (FTIR)

The FTIR spectrum of the synthesized MOF confirms its successful formation through the presence of characteristic functional groups. A broad band at 3439 cm⁻¹ corresponds to O–H stretching from adsorbed water, while a weak signal near 2354 cm⁻¹ is related to atmospheric CO₂ or carboxylate stretching. Strong peaks at 1635 and 1559 cm⁻¹ indicate C = O stretching of coordinated carboxylates, with additional bands at 1454 and 1380 cm⁻¹ supporting carboxylate vibrations. Peaks in the range 1173–1024 cm⁻¹ are attributed to C–N/C–O bonds, while signals at 798 and 729 cm⁻¹ denote aromatic ring bending. Finally, bands at 620 and 491 cm⁻¹ confirm metal–oxygen (M–O) linkages. Figure 3 a, b,c&d collectively, these results verify the hybrid organic–inorganic structure of the MOF and its potential for strong dye adsorption via hydrogen bonding, electrostatic interactions, and π–π stacking.

### Thermal stability and decomposition behaviour (TGA)

The TGA profiles of the synthesized MOFs (25–800 °C, N₂ atmosphere) revealed multistep weight losses. An initial loss below 250 °C corresponds to removal of adsorbed water and residual solvents. The main decomposition between 250 and 500 °C is attributed to the breakdown of organic linkers, with samples (c) and (d) showing delayed degradation, reflecting stronger metal–ligand coordination and higher crystallinity. Above 500 °C, stable metal oxide residues (e.g., ZnO, Cr₂O₃, ZrO₂) remain. Notably, sample (d) retained > 60% mass, indicating superior thermal stability and high metal content, while sample (a) showed only ~ 20% residue, confirming lower framework robustness.

### Scanning electron microscope (SEM) analysis

The surface morphology of the synthesized metal-organic framework (MOF) was examined using scanning electron microscopy (SEM), as shown in the micrograph above. The SEM image, captured at a magnification of 20,000×, reveals important structural characteristics that directly impact the material’s adsorption performance^27,28^. The MOF particles exhibit a heterogeneous, irregular, and highly porous structure, composed of aggregated and partially crystalline flakes with rough surface textures. These particles appear loosely packed, with visible voids and gaps between them, indicating the presence of interconnected porous channels. This porosity is essential for facilitating the diffusion and adsorption of dye molecules into the internal structure of the MOF. The flake-like morphology observed is consistent with many MOF materials; especially those synthesized using solvothermal methods. The particle size is in the submicron range, mostly below 1 μm, as indicated by the scale bar. Such small particle size and large surface irregularity enhance the surface area available for interaction with contaminants, thus improving the adsorption kinetics^29^. Furthermore, the absence of large cracks or collapse of the framework suggests good mechanical stability and structural integrity, which are desirable for repeated adsorption desorption cycles.

### Crystallinity and phase composition (XRD)

The XRD pattern of the synthesized MOF displays sharp peaks within 5°–40° (2θ), confirming its crystalline nature. Strong reflections at 7.5° and 8.5° indicate large pore sizes and a highly porous framework, while the peak at 25.7° may correspond to higher-order reflections or residual oxides. The absence of broad humps excludes amorphous phases, verifying the successful formation of a well-ordered crystalline MOF.

The XRD diffraction pattern of UiO‑66‑NH₂ exhibited characteristic peaks at approximately 2θ = 7.4° (111), 8.5° (200), 12.1° (211), and 25.7° (higher‑order reflection), which are consistent with the standard UiO‑66 framework. The absence of extra peaks suggests high phase purity and successful incorporation of the amine‑functionalized linker.

### EDX and elemental mapping

EDX analysis confirmed the expected elemental composition of UiO-66-NH₂, showing signals for Zr, C, O, and N. Elemental mapping demonstrated a uniform distribution of the metal clusters and organic linkers throughout the MOF structure. After dye adsorption, sulfur signals were detected on the adsorbent surface, confirming successful uptake of dye molecules.

**Fig. 4:**
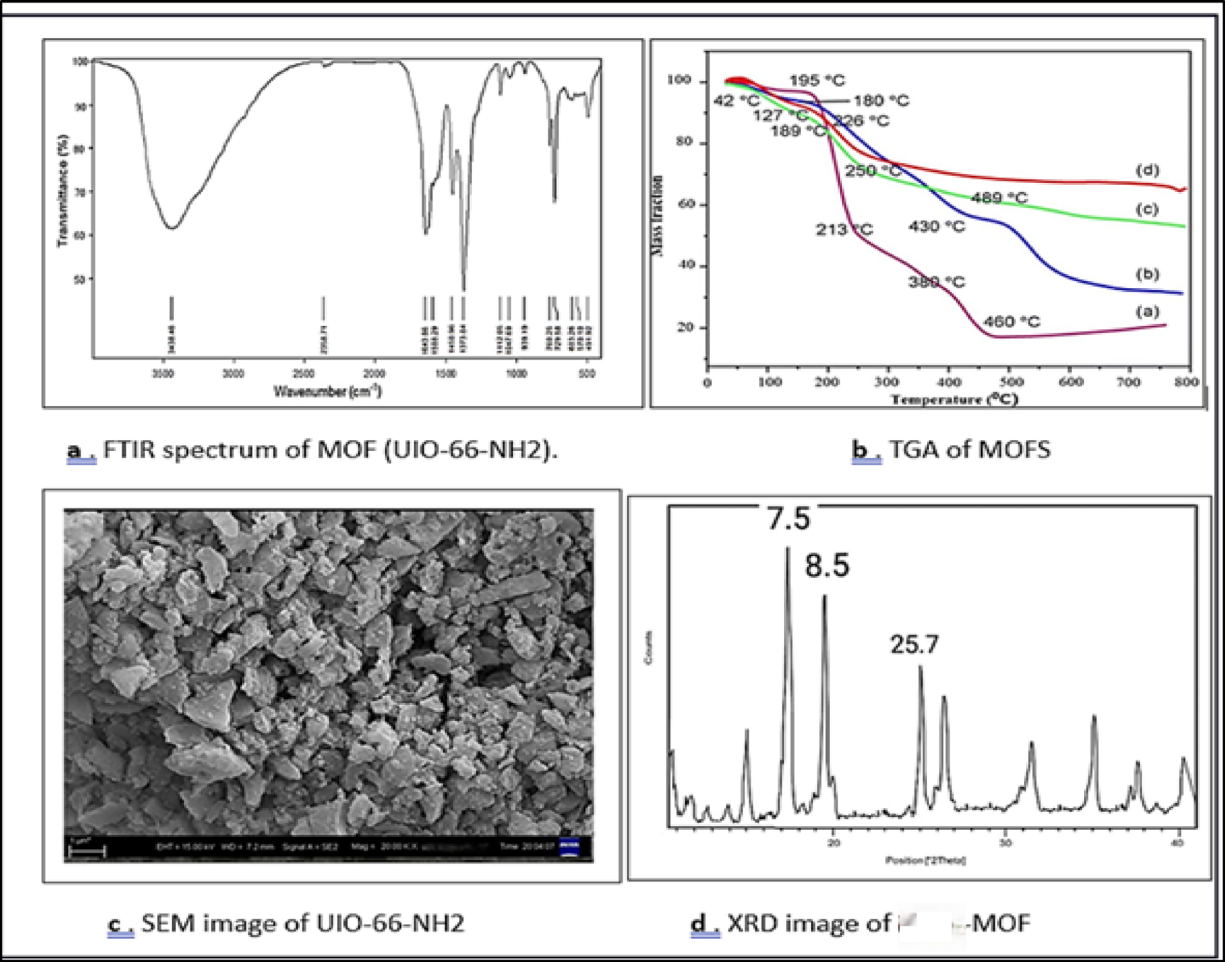
Characterization of MOF (a-FTIR, b-TGA, c-SEM, d-XRD).

### Effect of initial dye concentration

As shown in Tables 3, 4, 5 and 6 and Figure 4, the dye removal by fresh alga *U. fasciata* is dependent on the starting dye concentration. It was clear that when the starting dye concentration is raised, the removal of the dye reduces until it achieves its minimum value at the greatest concentration for each color independently after 8 h. At the lowest dye concentration, the highest dye removal percentage using fresh *U. fasciata* under varying initial dye concentrations was observed for Reactive Blue 19, reaching 76.70% at 20 mg/L after 8 h. In contrast, maximum removal for Reactive Yellow 2 was 66.44% at 30 mg/L, and for Reactive Red 195, it was 44.25% at 10 mg/L, both also recorded at 8 h. The same trend was confirmed by the results obtained by Pratiwi et al.^30^ who proved that the rate of methylene blue (MB) dye removal decreases with an increase in initial dye concentration, and this could be owing to the development of a monolayer over the surface of the adsorbent at a lower beginning concentration of dye. The highest dye removal efficiency using fresh *P. capillacea* under varying initial concentrations was observed for Reactive Red 195, reaching 73.67% at 10 mg/L after 8 h. For Reactive Yellow 2 and Reactive Blue 19, the maximum removal percentages were 53.78% at 30 mg/L and 81.96% at both 40 mg/L and 8 h, respectively.

The fresh *P. capillacea* exhibited a similar pattern as *U. fasciata*, except for RB19 dye, which had the lowest removal percentage (56.86%) at the lowest dye concentration (20 mg/l). Statistical analysis indicated indicating that the biosorption process was strongly dependent on the initial dye load, with lower concentrations generally yielding higher removal percentages. Generally, the removal of the dyes is inversely proportional to the starting dye concentration.

**Fig. 5:**
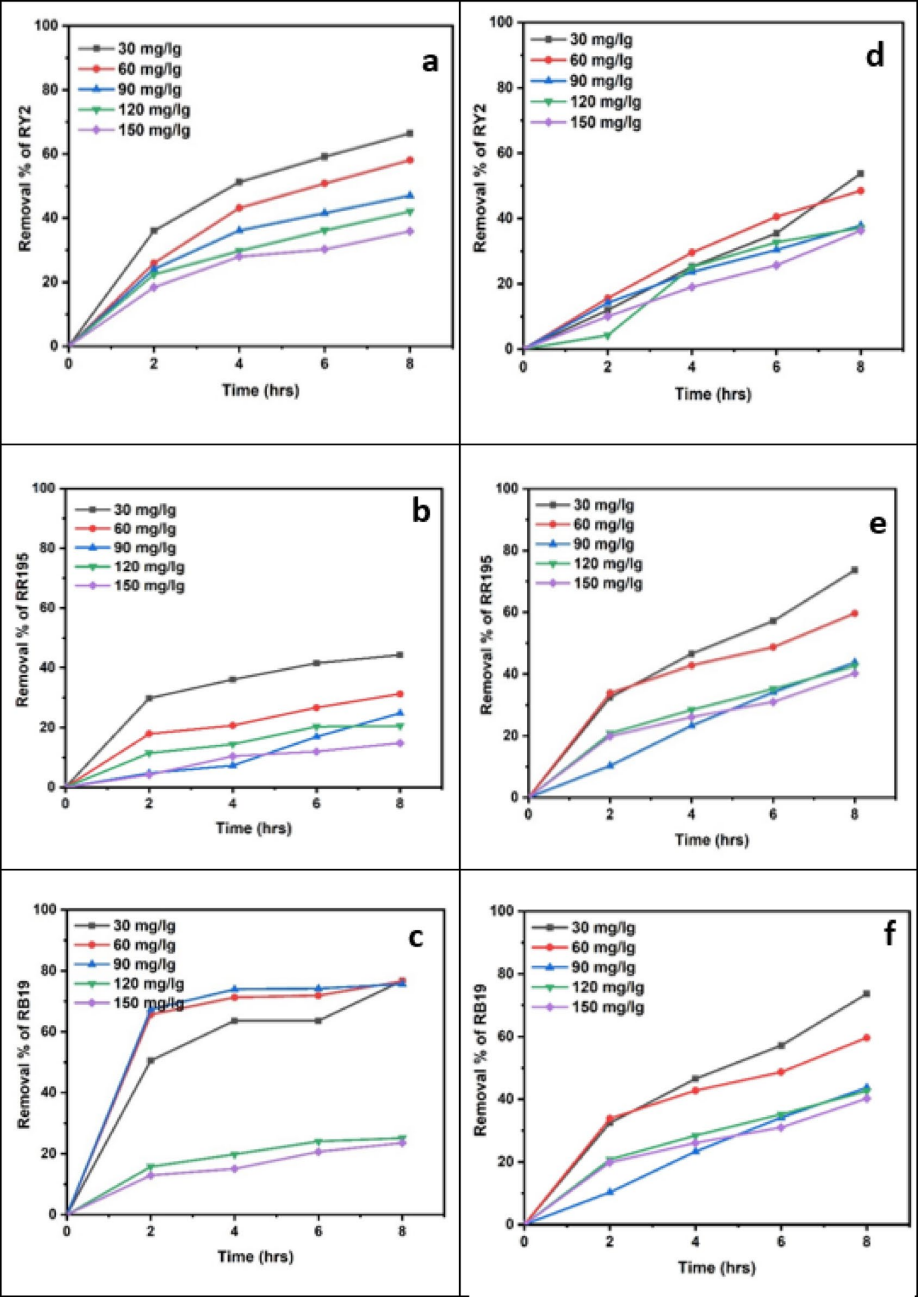
Effect of Different Initial Dye Concentrations on the Removal of RY2, RR195, and RB19 by fresh *Ulva fasciata* (**a**,** b**,**c**) and *Pterocladia capillacea* (**d**,** e**,**f**).

Obviously, the dried biomass exhibited approximately the same removal pattern compared to the fresh biomass, where the dye removal decreased somehow with the increase of the initial dye concentration. As shown in Tables 7 and 8 and Figure 5 a, b,c, the removal percentage of RY2 didn’t exhibit a significant change. The maximum dye removal percentage using dried *U. fasciata* was observed for Reactive Blue 19, reaching 65.35% at 20 mg/L after 8 h. For Reactive Yellow 2 and Reactive Red 195, the highest removal efficiencies were 47.93% at 90 mg/L and 55.34% at 20 mg/L, respectively, also recorded at 8 h.

The dried *P. capillacea* (Table 8; Figs. 5d, e,f) with both RY2 and RR195 exhibited the same removal tendency as fresh alga, with dye removal being inversely proportional to dye concentration. While all removal percentages of RB19 dye improved steadily with increasing dye concentration until reaching their maximum values (52.90%) at particular concentrations (40 mg/l), the removal percentages dropped above these concentrations. The statistical results confirm that higher biosorption efficiency is strongly dependent on dye concentration and contact time, reinforcing the suitability of both dried *U. fasciata* and *P. capillacea* as an effective eco-friendly adsorbent.

**Xie** et al.^31^ reported that the alive microalgae *Chlorella sorokiniana* removed 83% of 60 mg/L disperse blue 2BLN in 6 days. According to Al Hamadi et al.^32^, dead *Spirulina platensis* can remove 98.55% of 100 mg/L of Acid Black 210 (197 g/g removal capacity) in 1 h. The previous findings showed that fresh and dried algae’s performance to remove the three dyes under test is significantly impacted by the variation in starting dye concentration. The enormous driving power that the higher initial dye concentration provided to overcome all dye reactance between the aqueous and solid phases is likely, what caused the rise in dye removal. Increasing the initial dye concentration also results in more dye anions colliding with biomass, which enhances adsorption. These results coincide with those of other authors^33,34^. In addition, our findings are confirmed by those from **Omar**^35^ who noticed that quick decolorization or color dye effluent in the presence of microalgae strains may result from a strong attraction between the dye molecules and the microalgae, followed by rapid diffusion onto the external surface and rapid diffusion within the microalgal cells to ensure quick equilibrium.

**Table 5:**
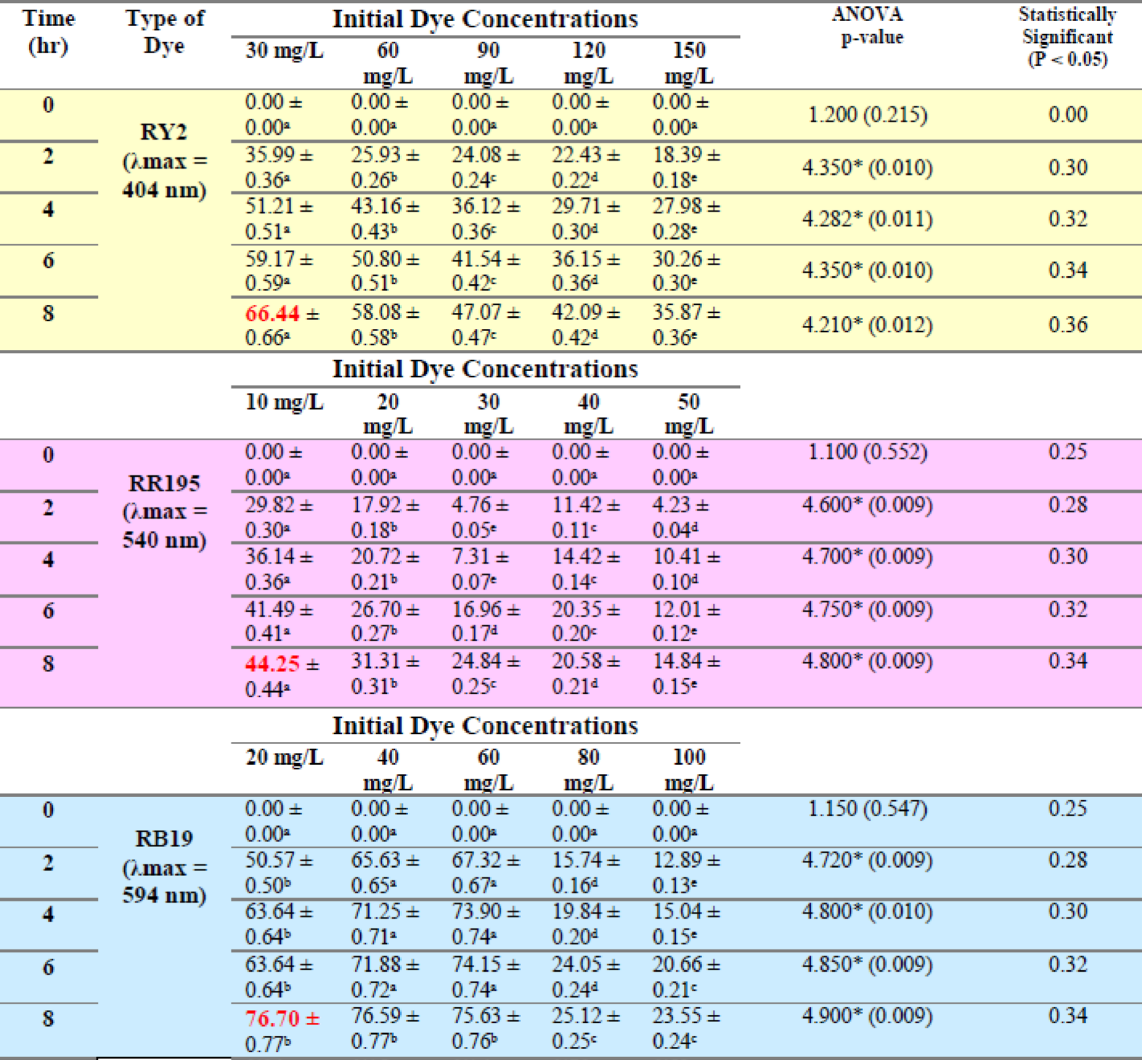
Effect of different initial dye concentrations (mg/L) on the removal % of three reactive dyes by fresh *Ulva fasciata*.

LSD: Least significant difference at 0.05. * : Statistically significant at *p* ≤ 0.05. ** : Statistically significant at *p* ≤ 0.01. Different subscripts are significant. Data are expressed in mean ± SD.

**Table 6:**
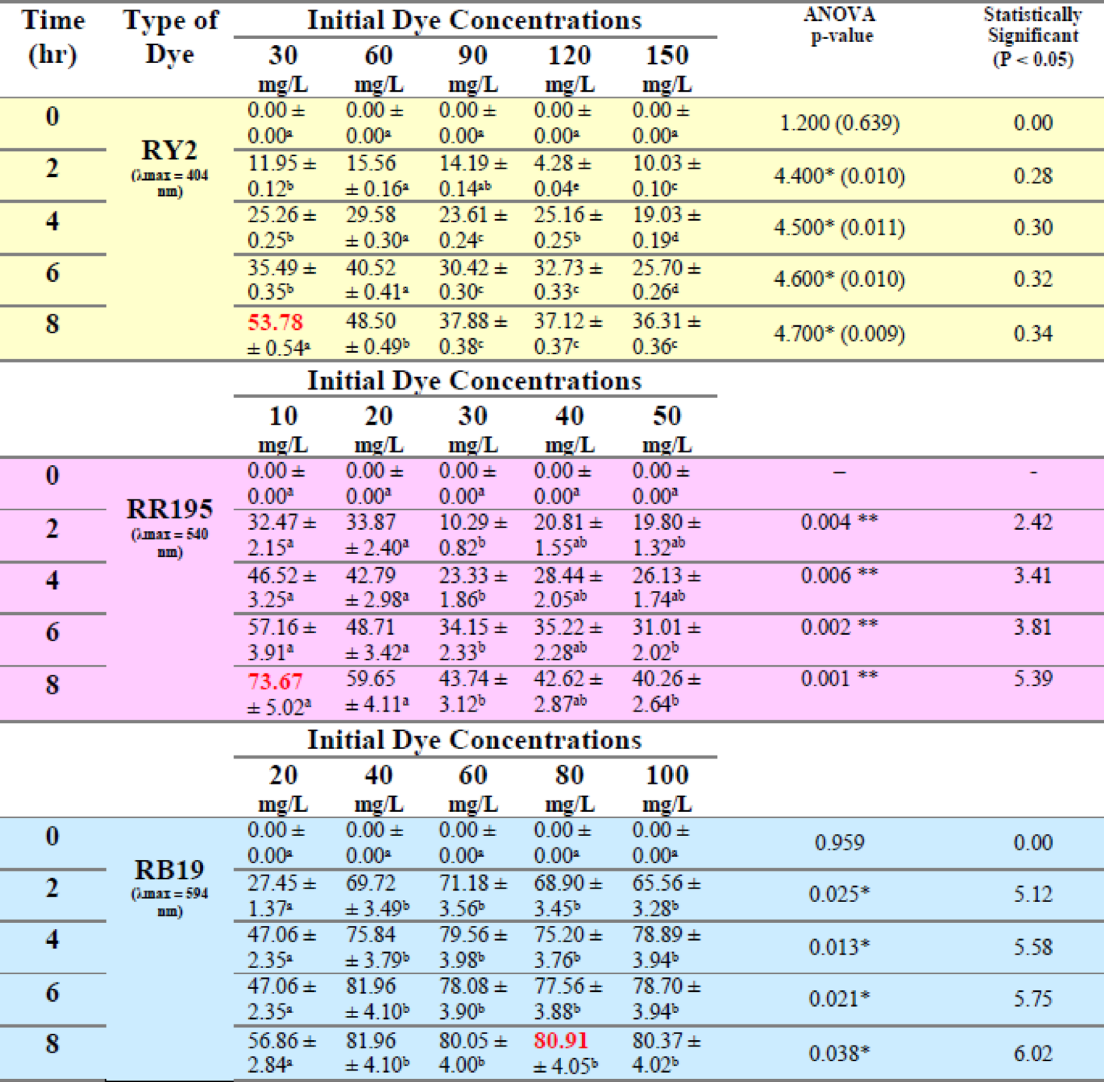
Effect of Different Initial Dye Concentrations (mg/L) on the Removal % of three Reactive Dyes by fresh *Pterocladia capillacea.*

LSD: Least significant difference at 0.05. * : Statistically significant at *p* ≤ 0.05. ** : Statistically significant at *p* ≤ 0.01. Different subscripts are significant. Data are expressed in mean ± SD.

**Table 7:**
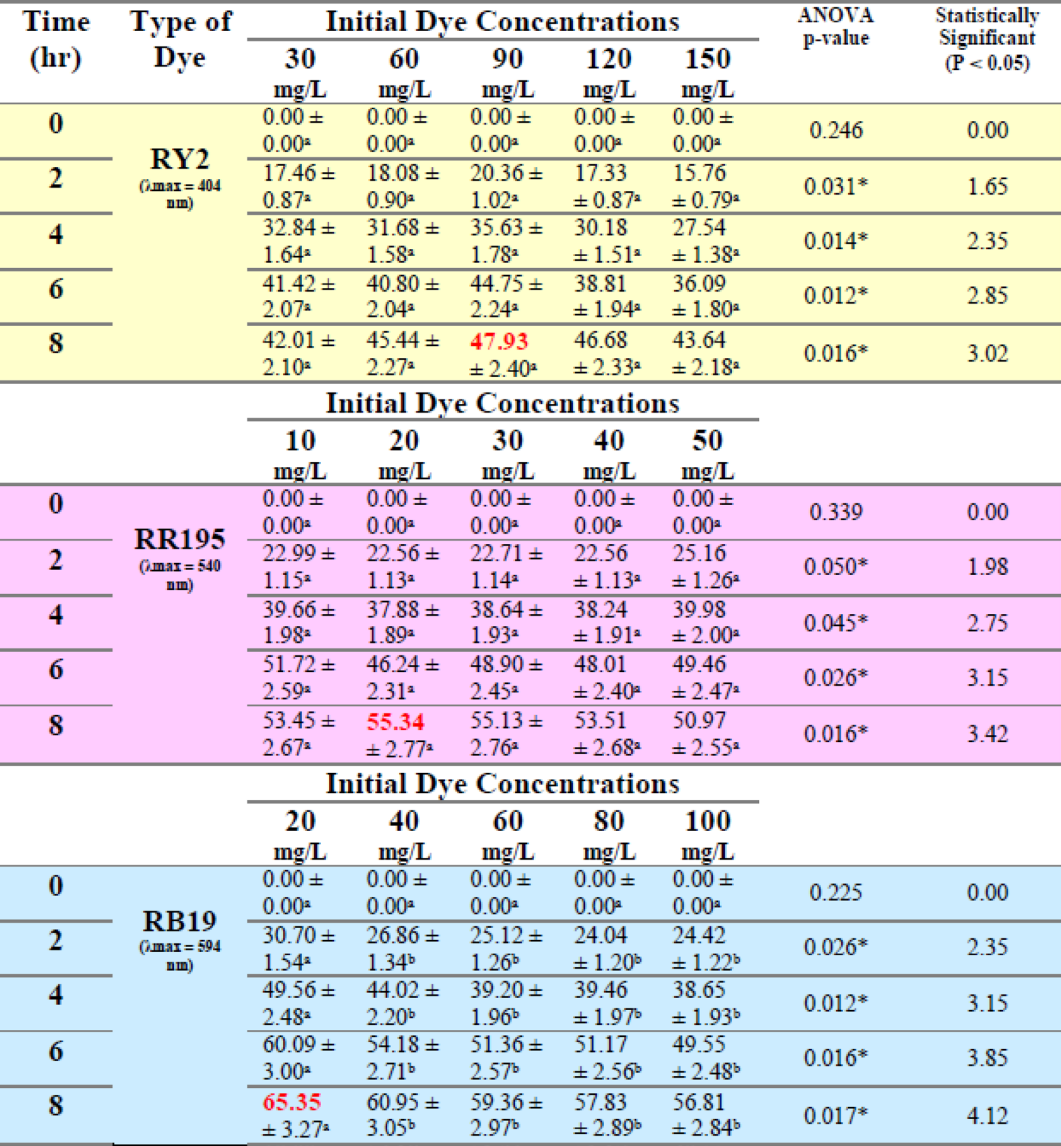
Effect of different initial dye concentrations (mg/L) on the removal % of three reactive dyes by dried *Ulva fasciata.*

LSD: Least significant difference at 0.05. * : Statistically significant at *p* ≤ 0.05. ** : Statistically significant at *p* ≤ 0.01. Different subscripts are significant. Data are expressed in mean ± SD.

**Table 8:**
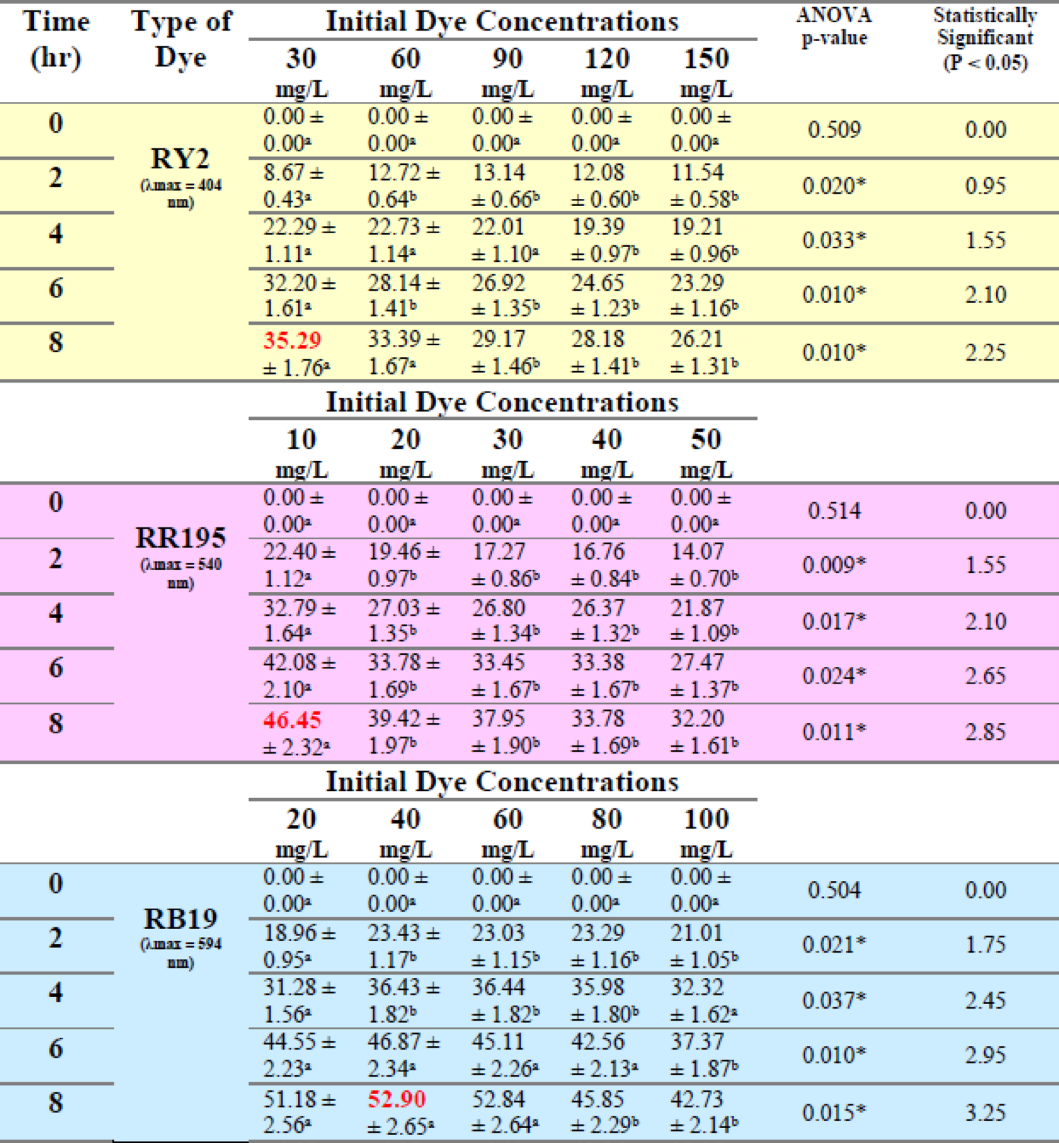
Effect of different initial dye concentrations (mg/L) on the removal % of three reactive dyes by dried *Pterocladia capillacea.*

LSD: Least significant difference at 0.05. * : Statistically significant at *p* ≤ 0.05. ** : Statistically significant at *p* ≤ 0.01. Different subscripts are significant. Data are expressed in mean ± SD.

**Fig. 6:**
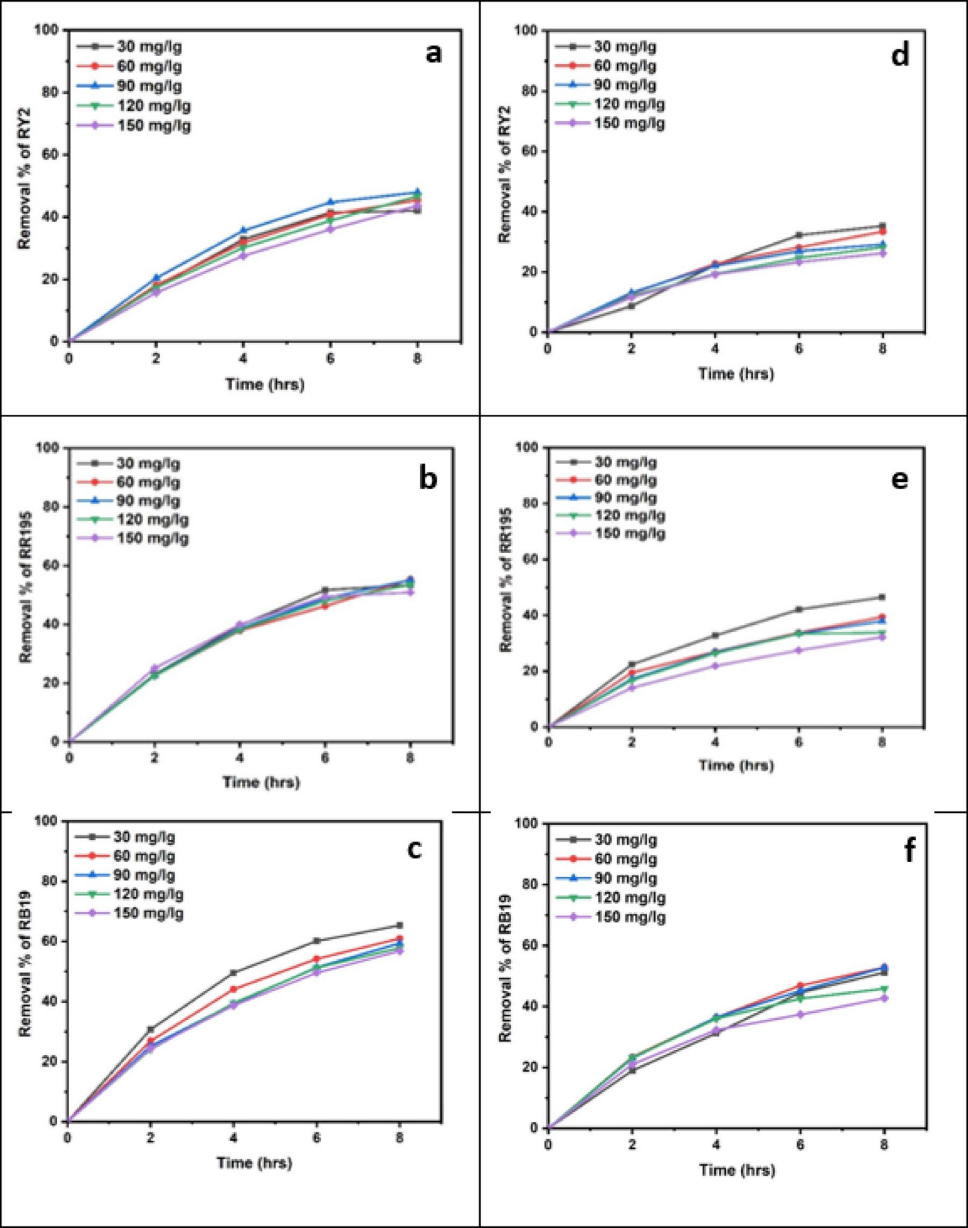
Effect of Different Initial Dye Concentrations on the Removal of RY2, RR195, and RB19 by dried *Ulva fasciata* (**a**,** b**,**c**) and *Pterocladia capillacea* (**d**,** e**,**f**).

### Adsorption evaluation (MOF)

At constant condition to study time contact of dyes (blue, yellow, red) at room temperature, PH 2, dose 1gm, rpm 150. The removal efficiencies of three reactive dyes, Reactive Blue, Reactive Red, and Reactive Yellow across eight successive stages of treatment. After 1 h 1, the removal efficiencies were relatively low, recorded at approximately 15% for Reactive Blue, 12% for Reactive Red, and 14% for Reactive Yellow. After 2 h, values increased to 30%, 26%, and 28%, respectively. After 3 h, the efficiencies reached 46% (Blue), 41% (Red), and 43% (Yellow)^36^. A further rise was observed After 4 h, with removal rates of 58%, 54%, and 56%, followed After 5 h, which recorded 70%, 67%, and 69%, respectively. After 6 h, the efficiencies climbed to 80% for Blue, 78% for Red, and 82% for Yellow. After 7 h demonstrated high performance with 90%, 88%, and 91% removal. Finally, after 8 h achieved near-complete removal with 97% (Blue), 96% (Red), and 97% (Yellow)^37^. Overall, the results show a consistent upward trend in removal efficiency for all dye types, with Reactive Blue maintaining a slight advantage during increasing time, Reactive Yellow performing closely behind, and Reactive Red showing marginally lower values. By the final stage, the differences between the three dyes became negligible, confirming the high efficiency and broad applicability of the treatment method for different reactive dyes^38^.

### The effect of adsorbent dose (MOF)

The effect of adsorbent dose revealed a clear positive correlation with dye removal efficiency, increasing from 55 to 65% at 0.5 g to over 90% at 2.0 g. blue and yellow dyes consistently showed higher adsorption than red, likely due to stronger electrostatic interactions and better compatibility with the adsorbent surface, while the red dye exhibited lower efficiencies, possibly due to steric hindrance or weaker binding affinity. The observed improvement with increasing dosage is attributed to the greater availability of active sites, although the diminishing gains at higher doses suggest that equilibrium adsorption is reached.

The reusability study revealed high initial dye removal efficiencies (85%, 80%, and 83% for blue, red, and yellow, respectively), but performance progressively declined with successive cycles due to active site deterioration and pore blockage. By the fourth cycle, efficiencies had dropped to 50%, stabilizing at 43–45% after six cycles, indicating limited long-term reusability without regeneration optimization. The consistent decline across all dyes suggests that the loss of adsorption capacity is mainly attributed to structural and surface fatigue of the adsorbent rather than dye-specific effects.

### Effect of different pHs

As shown in Tables 9 and 10, both freshly studied algal species revealed great removal abilities at lower pH values, specifically pH 2. According to the dye constituents and microalgae physiology, any parts of the microalgae cell perform critical tasks at certain pH levels^19^. A remarkable outcome was the maximal elimination percentage of RR195 (100%) at pH2 by fresh *U. fasciata*, as shown in Figure 6b. For Reactive Yellow 2 and Reactive Blue 19, the highest removal percentages were 70.00% at pH 6 and 94.06% at pH 2, respectively, both also recorded at the 8-hour mark. The pH of the biosorption processes using algae was previously demonstrated by Kumar et al.^39^ to be considerably dependent, making it one of the most important parameters to take into account. They also discussed how dye chemistry in water and solution pH impacts the binding sites for cell surface dyes. The highest removal efficiency by fresh *P. capillacea* under varying pH levels was recorded for Reactive Blue 19, reaching 97.88% at pH 2 after 8 h. For Reactive Red 195 and Reactive Yellow 2, the maximum removal percentages were 94.00% at pH 2 and 76.32% at both pH 2 and 6, respectively, also after 8 h of contact time (Table 10; Figures 6d, e,f). Statistical analysis confirms that biosorption performance is strongly pH-dependent, with optimal removal typically occurring at acidic to neutral conditions depending on the dye. These findings highlight the importance of pH optimization in enhancing the effectiveness of marine algae-based dye remediation.

**Fig. 7:**
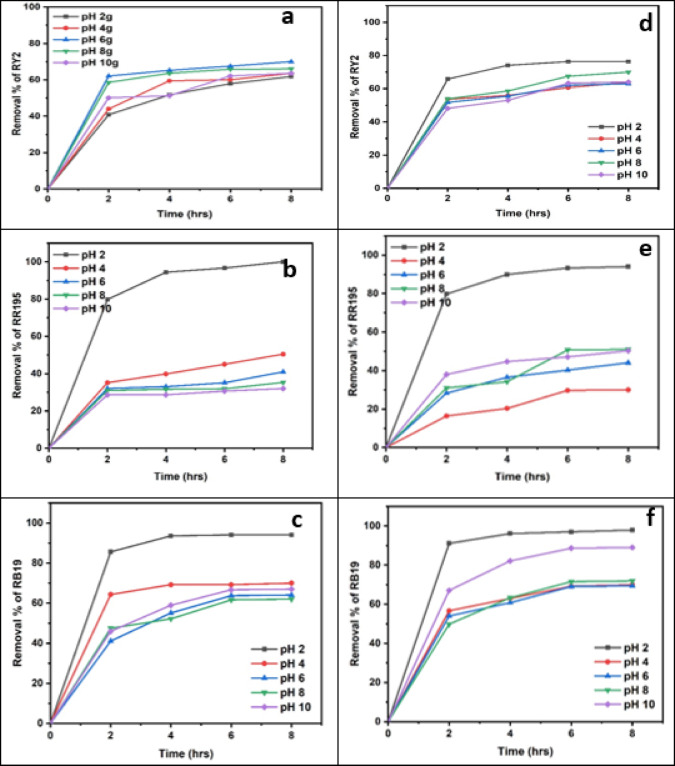
Effect of Different pHs on the Removal of RY2, RR195, and RB19 by fresh *Ulva fasciata* (**a**,** b**,**c**) and *Pterocladia capillacea* (**d**,** e**,**f**).

Regarding the dried biomass, As shown in Tables 11, 12, 13, 14, 15, 16 and Fig. 7, the removal percentages of the three dyes using dried *U. fasciata* and *P. capillacea* were also high at pH 2 and decreased with increasing the pH values. The highest removal efficiency by dried *U. fasciata* under varying pH levels was observed for Reactive Blue 19, reaching 63.37% at pH 2 after 8 h. For Reactive Red 195 and Reactive Yellow 2, the maximum removal percentages were 60.16% at pH 2 and 57.11% at pH 2, respectively, also at the 8-hour mark. The highest dye removal efficiency using dried *P. capillacea* was observed for Reactive Red 195, reaching 73.03% at pH 2 after 6 h. For Reactive Yellow 2 and Reactive Blue 19, the maximum removal percentages were 47.37% at pH 2 and 67.82% at pH 2, respectively, both recorded at the 8-hour contact time. The previous results demonstrated that pH 2 has a significant impact on both *U. fasciata* and *P. capillacea’s* capacity to remove all dyes efficiently. Statistical analysis indicated that all dyes exhibited statistically significant differences in removal percentages across pH levels (*p* < 0.05), confirming that pH plays a critical role in optimizing dye removal efficiency. These findings emphasize the necessity of pH adjustment when applying dried *U. fasciata* and *P. capillacea* in eco-friendly wastewater treatment.

Maximum dye removal occurred at acidic pH. Blank controls demonstrated negligible dye loss in the absence of adsorbents, confirming that removal was due to adsorption rather than precipitation.

This may be explained by the fact that hydronium ions (H^+^) surround the algal membrane at low pH values, creating positively charged binding sites that increase the electrostatic attraction between the negatively charged dye anion and the positively charged cell surface. This leads to higher removal values at pH 2.0. It is believed that at acidic pH values, nitrogen-containing functional groups in biomass, such amines or imidazole, will be protonated. However, when the pH of the solution rises, there are more negatively charged sites and fewer positively charged sites. A negatively charged surface location on the algal biomass does not promote the adsorption of dye anions because of electrostatic repulsion^40^.

Our interpretations agree with Hazrat and Muhammad^41^ who mentioned that protonated carboxyl groups become positively charged at lower pH values; these positively charged COO- groups might attract negatively charged reactive dye molecules, enhancing binding. Cardoso et al.^26^(2002) also demonstrated that, as compared to neutral and alkaline solutions, Acid Green 3 dye absorption was much greater in acidic solutions. With the same respect, Rosli et al.^1^. reported that the optimum pH for bio-removal of BG1 was 4 for RB19 using different algal biomass. The decline in dyes’ capacity to adsorb on algae as pH increases can be attributed to changes in surface characteristics and charge. Gül et al.^42^ also showed that at pH 2, both biosorbent kinds of *Phormidium animale* (fresh and dried) displayed the best dye biosorption rates. Furthermore, dried *P. animale* biosorbent was more effective in biosorption than fresh *P. animale* biosorbent.

Contrary, according to Pathak et al.^24^. *Chlorella sp.* ability to absorb azo dye increases from 0 to 91% when the pH raised from 3 to 8 but starts to deteriorate between pH 8 and 10. On the other hand, El-Sheekh et al.^19^ demonstrated that *Oscillatoria sp*. had the maximum biosorption performance for Reactive Orange 122 at pH 11 (79.61%) and the lowest at pH 5 (56.31%) mixed with *S. obliquus*. Ghazal et al.^4^ reported that *Anabaena variabilis*,* Chlorella vulgaris*,* Nostoc linkia*,* Nostoc elepsosporum*, and *Anabaena flos aquae* had the maximum removal efficacy of synthetic dyes at 9.95 pH, 8.85, 8.72, 7.92, and 7.75, respectively, compared to 7.5 for the initial value (microalgae cultivated on their basal media). Pratiwi et al.^30^ determined that the marine algae *Ulva lactuca*’s capacity to eliminate Methylene blue dye was regulated by contact time, algal biomass, dye concentration, and pH. The value for the greatest percentage of dye removal was 91,92% at pH8. Also, Omar et al.^20^ demonstrated that at pH 8.0, *U. lactuca’*s biomass removed the most Malachite Green dye (93.8%), and that adsorption was almost constant.

Algae are not just passive biosorbents; they actively contribute to circular economy principles through biomass reuse, carbon sequestration, and nutrient recovery. The integration of algal-based remediation systems into wastewater infrastructure can lead to energy-efficient water treatment, which reduces the environmental footprint and fosters sustainable urban development^43,44^.

### Effect of pH for MOF

**Fig. 8:**
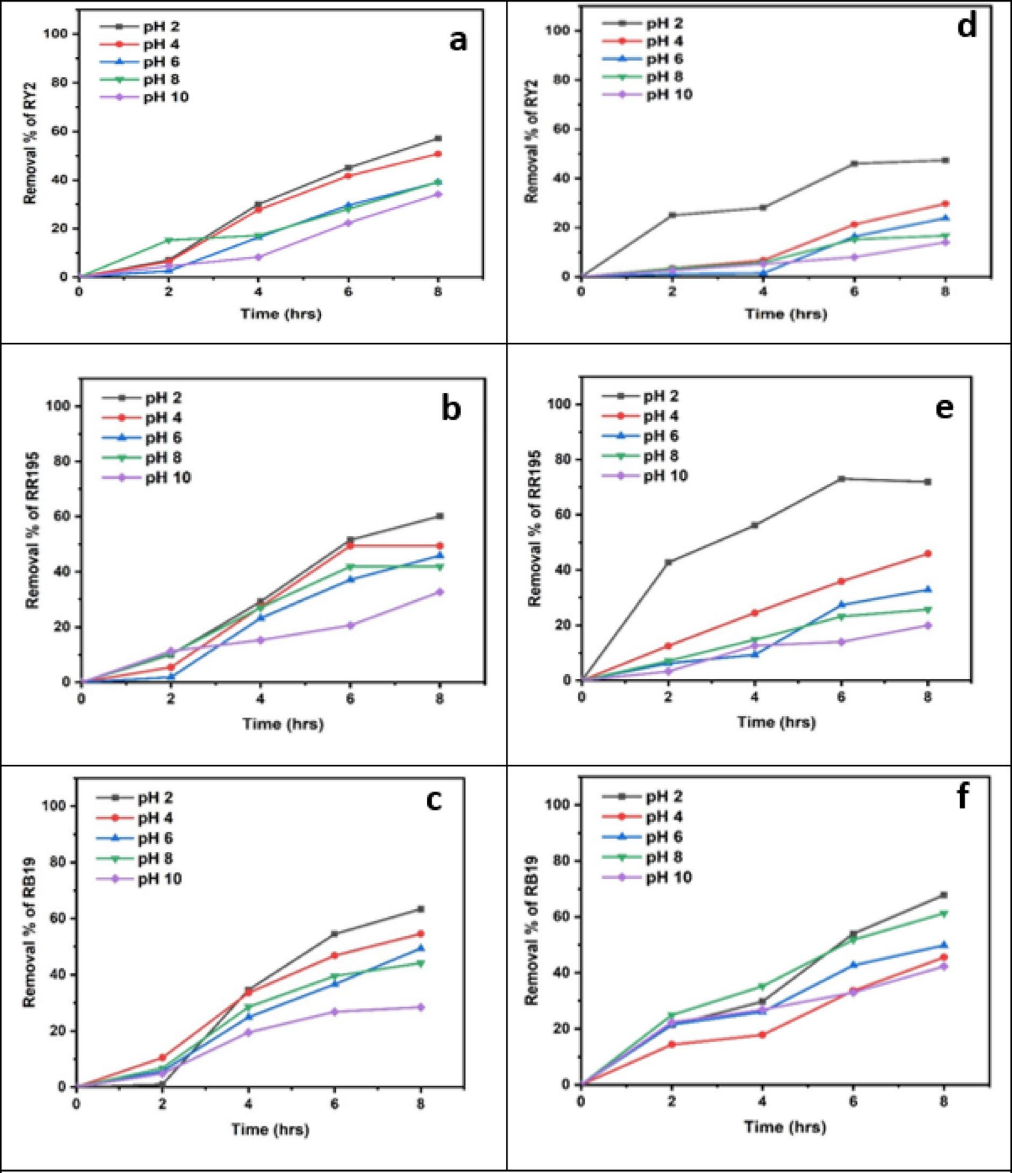
Effect of Different pHs on the Removal of RY2, RR195, and RB19 by Dried *Ulva fasciata* (**a**,** b**,**c**) and *Pterocladia capillacea* (**d**,** e**,**f**).

**Fig. 9:**
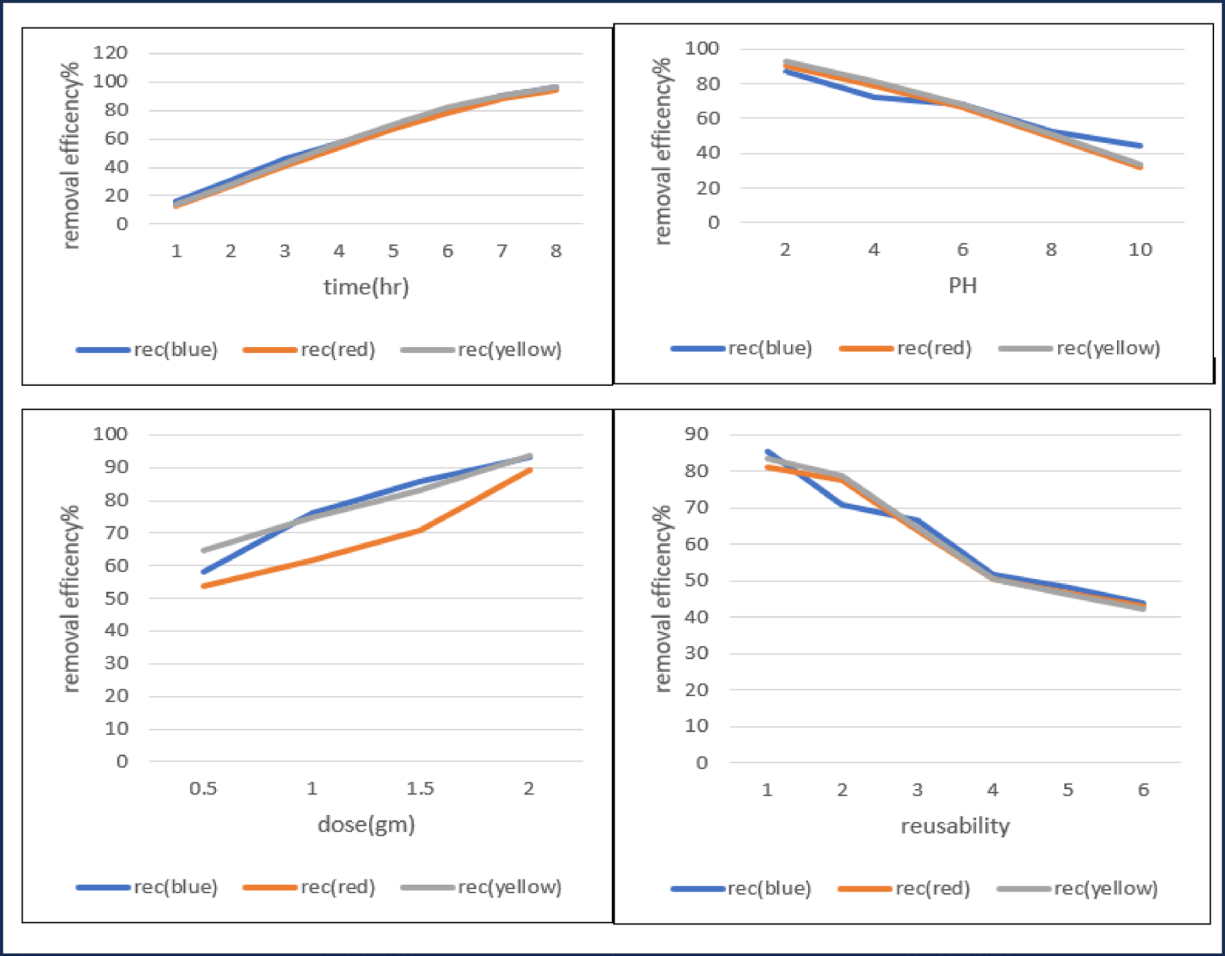
Effect of time, PH, dose and reusability on the adsorptive removal efficiency MOF at constant PH 2, Time 8 h, adsorption temperature (25 °C), and shaking speed (150 rpm).

At constant condition to study PH (2,4,6,8,10) of dyes (blue, yellow, red) at room temperature, dose 1gm, rpm 150. The effect of pH on the removal efficiency of reactive blue, red, and yellow dyes is presented in Figure 8. The data reveal a pronounced dependence of dye removal performance on the solution pH, with maximum efficiencies observed under acidic conditions (pH 2) and a gradual decline as the pH increased toward alkaline conditions (pH 10). At pH 2, removal efficiencies exceeded 85% for all dyes, with reactive yellow demonstrating the highest performance (92%). This enhancement in acidic media is likely due to the protonation of functional groups on the adsorbent surface, which promotes electrostatic attraction between the positively charged adsorbent sites and the anionic sulfonate groups of reactive dyes^45^. At moderate pH values (4–6), removal efficiencies decreased, although they remained relatively high (above 65%), indicating that electrostatic attraction still contributed significantly to the adsorption process, albeit reduced due to partial deprotonation of the adsorbent surface. The difference among the three dyes at these pH levels may be related to variations in their molecular size, charge density, and affinity toward the functional groups of the adsorbent^46^. In alkaline conditions (pH 8–10), As shown in Fig. 9a sharp decline in removal efficiency was observed, particularly at pH 10, where values dropped below 50% for reactive blue and even lower (30–35%) for reactive red and yellow. This reduction is attributed to increased competition from hydroxide ions (OH⁻) for active sites and the deprotonation of surface functional groups, which results in a net negative surface charge. The resulting electrostatic repulsion between negatively charged adsorbent surfaces and anionic dye molecules substantially inhibits adsorption. These results align with the general adsorption theory for anionic dyes, where acidic conditions Favor adsorption through electrostatic attraction, while alkaline conditions reduce efficiency due to surface charge reversal and ion competition. Optimizing pH control during treatment is therefore critical to maximizing dye removal efficiency^47^.

Point of Zero Charge (pH pzc): The pH pzc was determined to be 6.3, indicating the surface is positively charged in acidic conditions, enhancing adsorption of anionic dyes.

**Fig. 10:**
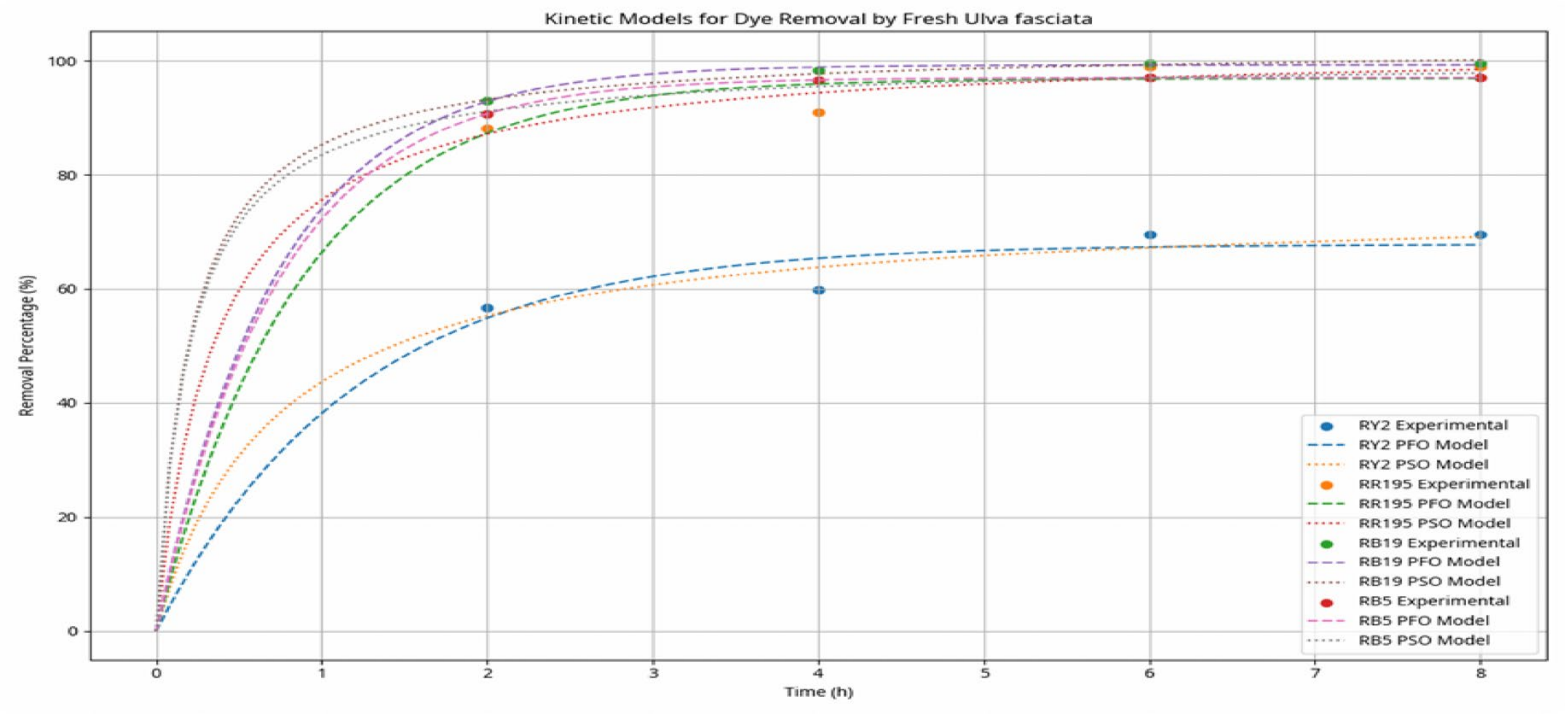
The following graph illustrates the experimental data points and the fitted pseudo-first order and pseudo-second-order kinetic models for each dye by using green algae.

The correlation analysis revealed an exceptionally strong positive association between contact time and the removal efficiency of the three dyes (blue, red, and yellow). As shown in Fig. 10, The Pearson correlation coefficients were 0.993, 0.994, and 0.992 for blue, red, and yellow, respectively, all statistically significant at the 0.01 level (2-tailed). These coefficients, being very close to + 1, confirm that dye removal efficiency increases in an almost perfectly linear fashion with longer contact times. In addition, the inter-dye correlations were nearly perfect (*r* = 0.999–1.000), indicating that the three dyes responded in an almost identical manner under the applied experimental conditions. This consistency suggests that the adsorption/removal mechanism is essentially the same for all dyes, and that the differences in their removal efficiencies are minimal.

**Table 9:**
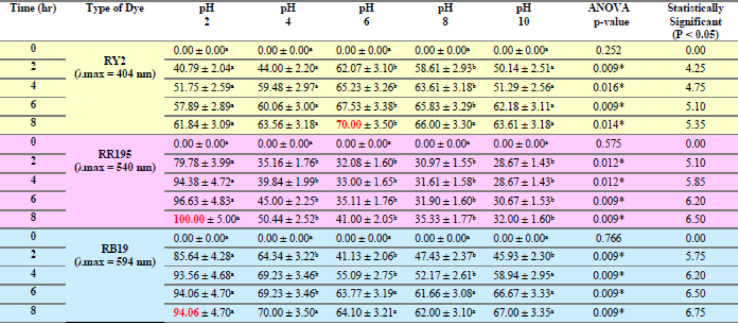
Effect of different phs on removal % of three reactive dyes using fresh algal biomass of *U. fasciata*.

LSD: Least significant difference at 0.05. * : Statistically significant at *p* ≤ 0.05. ** : Statistically significant at *p* ≤ 0.01. Different subscripts are significant. Data are expressed in mean ± SD.

**Table 10:**
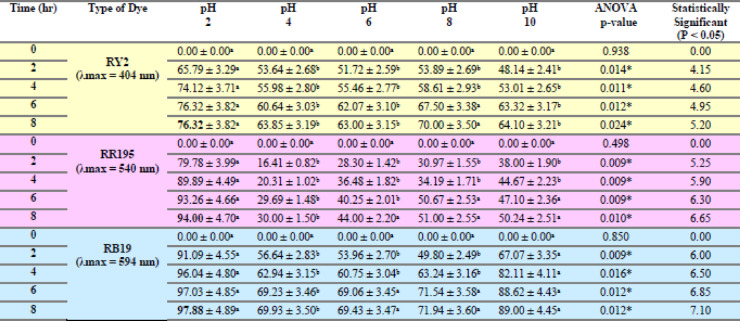
Effect of different phs on removal % of three reactive dyes using fresh algal biomass of *P. capillacea*.

LSD: Least significant difference at 0.05. * : Statistically significant at *p* ≤ 0.05. ** : Statistically significant at *p* ≤ 0.01. Different subscripts are significant. Data are expressed in mean ± SD.

**Table 11:**
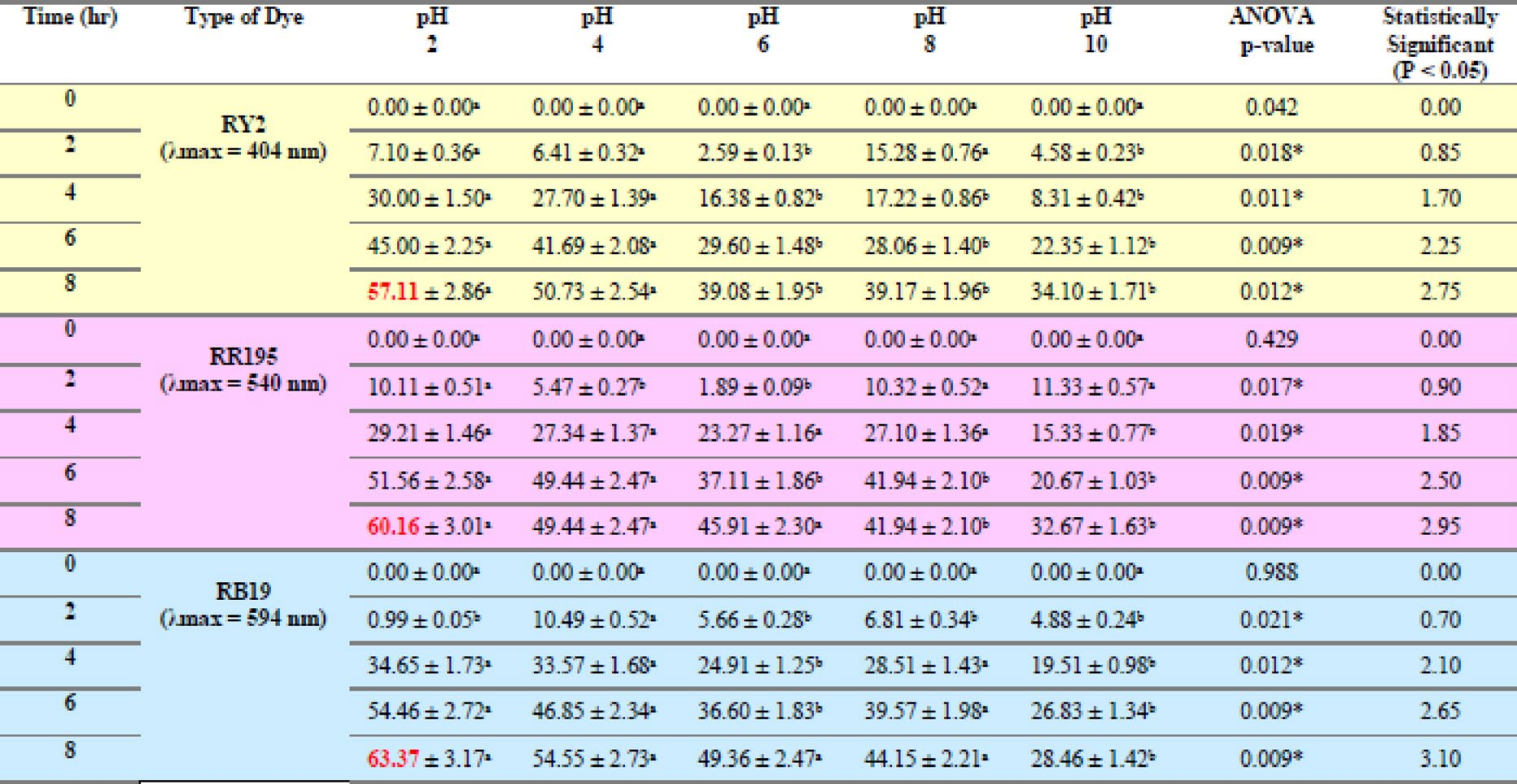
Effect of different phs on removal % of three reactive dyes using dried algal biomass of *Ulva fasciata*.

LSD: Least significant difference at 0.05. * : Statistically significant at *p* ≤ 0.05. ** : Statistically significant at *p* ≤ 0.01. Different subscripts are significant. Data are expressed in mean ± SD.

**Table 12:**
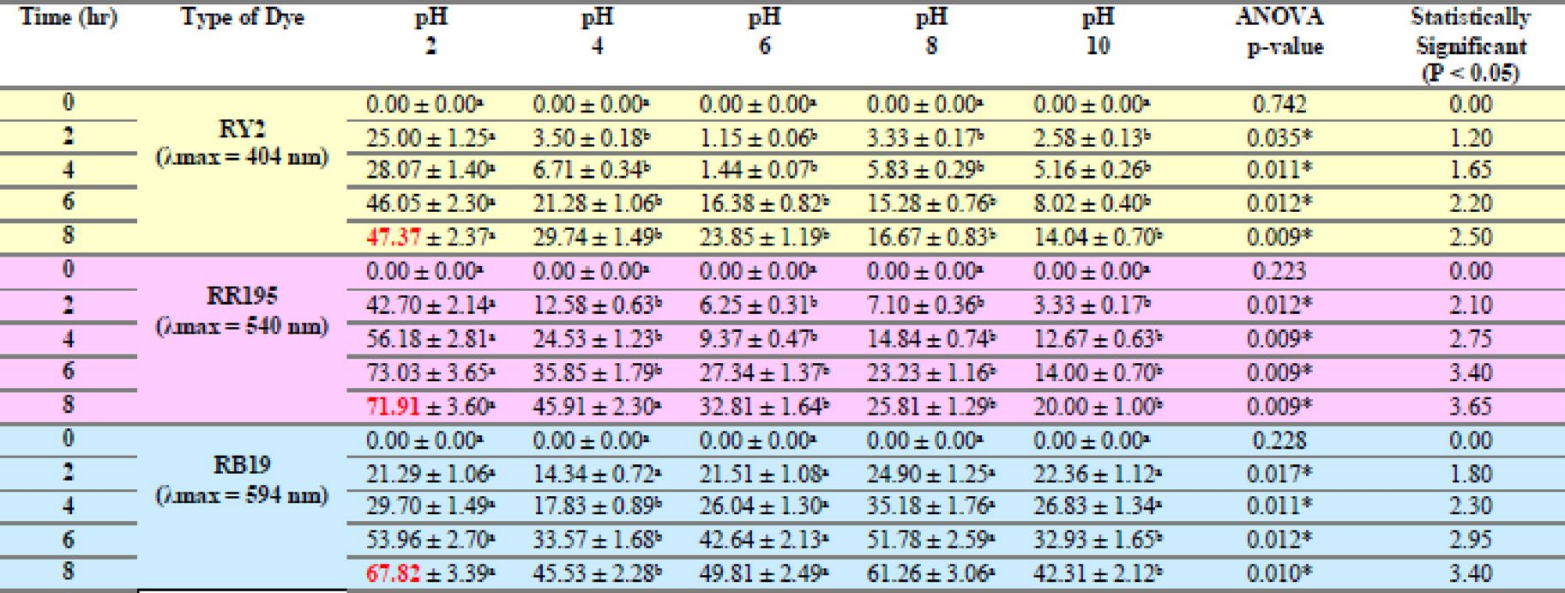
Effect of different phs on removal % of three reactive dyes using dried algal biomass of *Ulva fasciata*.

LSD: Least significant difference at 0.05. * : Statistically significant at *p* ≤ 0.05. ** : Statistically significant at *p* ≤ 0.01. Different subscripts are significant. Data are expressed in mean ± SD.

### Validation using real textile effluent

Real textile wastewater collected from a local dyeing facility was treated to evaluate the practical efficiency of the biosorbent–MOF system. The effluent initially exhibited high color intensity and elevated COD levels. Treatment experiments were performed under the same optimized laboratory conditions used for synthetic dye solutions.

**Table:** 

Parameter	Before Treatment	After Algae Treatment	After MOF Treatment
Color (Abs@λmax)	High	Reduced	Near-complete removal
pH	6.8	6.5	6.4
COD (mg/L)	420	240	118

The substantial decrease in color intensity and COD demonstrates the effective performance of the composite system in treating real industrial effluent. UiO‑66‑NH₂ exhibited superior removal efficiency compared to algae alone, confirming its strong adsorption capability under practical conditions.

### Adsorption kinetics

Illustrates the relationship between reactive dyes (yellow, red, and blue) pollutant ions adsorbed on both two tested algal species and the nanoparticle MOF with time. Adsorption process takes place through solution/solid interface then pollutants diffuse in the solution fluid film then contaminants move towards the active site in the nanomaterial then to the internal pores of the nanomaterial through intraparticle diffusion process followed by finally adsorption takes place of the pollutants at the adsorption sites^48^.Pseudo-first-order reactions Pseudo-first-order kinetics was applied to study the adsorption rate of reactive dyes on nanoparticles MOF at equilibrium. it undergoes a physical adsorption process^49–53^. Adsorption Isotherms: Langmuir model showed superior fitting (R² > 0.98), confirming monolayer adsorption with q_max = 127.4 mg/g. Adsorption Kinetics: The adsorption kinetics followed a pseudo‑second‑order model (R² > 0.97), suggesting chemisorption as the rate‑limiting mechanism.

Equation – represents pseudo-first-order reaction as follows:

2 $$ln(q\_e-q\_t)\hspace{0.17em}=\hspace{0.17em}ln(q\_e)-k\_1\mathrm{*}t$$

Where, qe is the quantity adsorbed at equilibrium (mg/g) and qt is the quantity absorbed at time t (mg/g). k1 is the rate constant for the pseudo-first-order sorption (min^− 1^). The slope of log (qe - qt) vs. t should be a straight line if the adsorption reaction is first order. Pseudo-second order reactions Pseudo-second order kinetics was applied to study the rate of reactive dyes removal at equilibrium. It undergoes a chemical adsorption process. 2nd eq sup. Data

3 $$\frac{1}{q_t}=\frac{1}{k_2{q^2_e}}+\frac{t}{q_e}$$

The adsorption kinetic behaviour of the studied dyes was evalu*ated using pseudo-first-order* and pseudo-second-order models, as illustrated in Fig. 10.

**Fig. 11:**
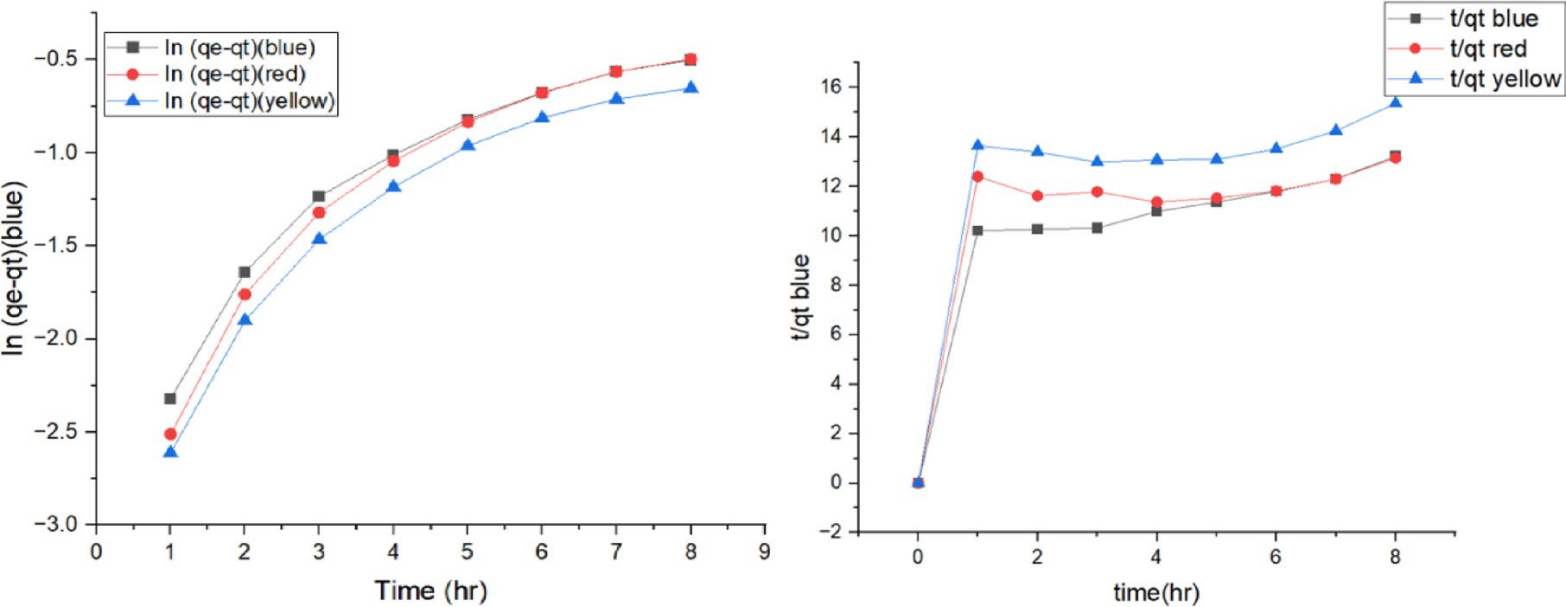
First-Second order reactions Plot for the adsorption process.

**Table 13:**
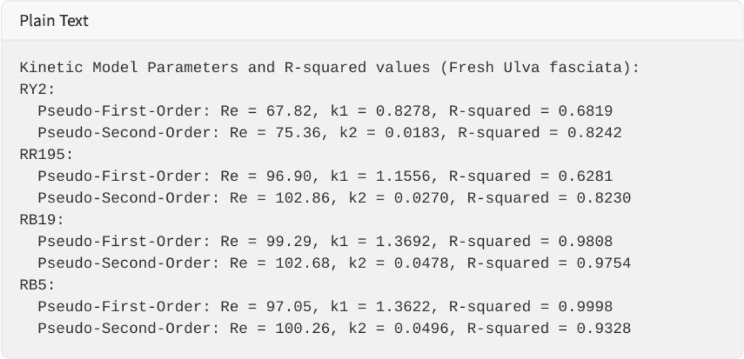
The following graph illustrates the experimental data points and the fitted pseudo-first order and pseudo-second-order kinetic models for each dye by using two marine green algae.

**Table 14:**
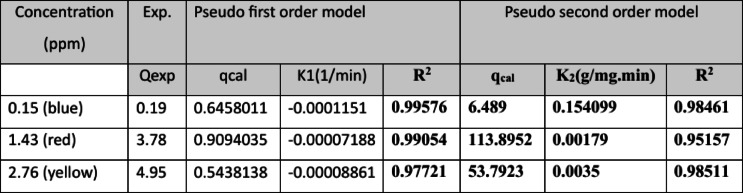
Adsorption kinetic parameters of pseudo first & second order for dyes adsorption.

**Table 15:**
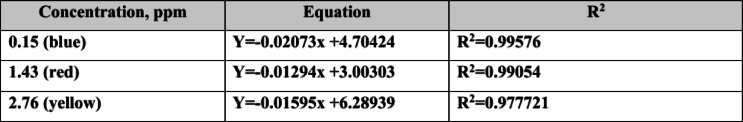
Pseudo-first order adsorption kinetic reactions.

**Table 16:**
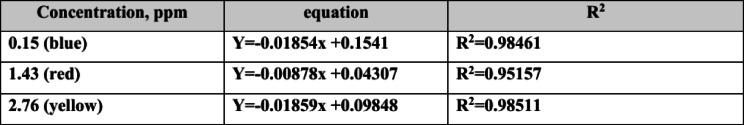
Pseudo-second order adsorption kinetic reactions.

The linear fitting of the pseudo-first-order and pseudo-second-order kinetic models is presented in Fig. 11.

## Conclusion

This study provides a comprehensive evaluation of two eco-friendly strategies for removing hazardous textile dyes from aqueous solutions: marine macroalgae (Ulva fasciata and Pterocladia capillacea) and the metal–organic framework UiO-66-NH₂.

### Algal biomass

The decolorization capacity of the algae was strongly influenced by biomass concentration, initial dye concentration, and solution pH. Both fresh and dried biomass exhibited high adsorption performance, with dried biomass proving more effective and easier to handle. For instance, dry U. fasciata achieved a maximum removal of 93.7% for RB19. Adsorption capacity increased with biomass dosage, reaching optimal removal at 5 g for fresh biomass and 2.5 g for dried biomass. High initial dye concentrations negatively affected removal efficiency, while acidic conditions (pH 2) favored adsorption. Under optimal conditions, fresh U. fasciata achieved complete decolorization of RR195. These results highlight the critical role of pH and confirm marine macroalgae as efficient, low-cost, biodegradable biosorbents.

### UiO-66-NH₂

Independently, the MOF exhibited outstanding adsorption performance, achieving over 95% removal under optimized conditions. Efficiency was strongly dependent on pH, contact time, and adsorbent dosage, with protonated MOF surfaces at low pH facilitating electrostatic interactions with anionic dyes. Characterization confirmed high crystallinity, porosity, and functional group availability, enabling strong interactions via hydrogen bonding, π–π stacking, and electrostatic forces. Kinetic studies indicated pseudo-second-order adsorption, suggesting chemisorption as the dominant mechanism. The MOF maintained good performance over three reuse cycles.

### Integrated perspective

Combining algal biomass with UiO-66-NH₂ into a hybrid system further enhances dye adsorption performance. However, separating the contributions of each adsorbent clarifies that algae and MOFs are effective independently, offering complementary mechanisms: algae provide biodegradable, naturally abundant adsorption sites, while MOFs offer highly porous, tunable, and multifunctional surfaces. Implementing integrated algae–MOF systems at an industrial scale can mitigate textile dye pollution, enhance water quality, and support sustainable resource management.

### Sustainability implications

Algae-based dye removal aligns with the United Nations Sustainable Development Goals, particularly SDG 6 (Clean Water and Sanitation), due to its low cost, high efficiency, and biodegradability. Hybrid systems combining algae and MOFs contribute to circular economy principles, reduce aquatic toxicity, and support ecological sustainability.

### Limitations of the study

Despite the promising findings, several limitations should be acknowledged. First, only three synthetic reactive dyes and two marine macroalgal species (Ulva fasciata and Pterocladia capillacea) were examined, limiting the generalizability of the results to other dye classes or biosorbents. Additionally, no regeneration or reuse studies were conducted for the algal biomass, which is important for evaluating economic feasibility and practical applicability at a larger scale.

For UiO-66-NH₂, the number of adsorptions–desorption cycles tested was limited, leaving the long-term stability and recyclability uncertain. Moreover, the MOF synthesis process is currently energy-intensive and relies on organic solvents, raising concerns regarding environmental sustainability and scalability.

Finally, the study was conducted at laboratory scale using synthetic dye solutions. The performance of both algae and MOFs in complex real industrial effluents, under long-term operation and varying environmental conditions, remains to be investigated in future studies.

These limitations highlight areas for further research, including exploration of diverse dye classes, additional algal species, biomass regeneration strategies, and development of greener, scalable MOF synthesis methods.

### Environmental Implications

The increasing discharge of industrial dyes into aquatic ecosystems poses significant environmental and health challenges. This study highlights two sustainable strategies for mitigating these impacts: marine macroalgae and metal–organic frameworks (MOFs).

### Marine algae

Renewable, biodegradable, and non-toxic marine biomass offers an eco-friendly alternative to conventional chemical treatments for dye-contaminated wastewater. The high adsorption efficiency of Ulva fasciata and Pterocladia capillacea supports sustainable wastewater treatment, directly contributing to the improvement of water quality and aligning with the United Nations Sustainable Development Goal 6 (Clean Water and Sanitation). Implementing algae-based bioremediation approaches can promote circular bioeconomy strategies and the development of sustainable algae processes for environmental remediation.

### Metal–organic frameworks (UiO-66-NH₂)

UiO-66-NH₂ provides a complementary sustainable approach due to its high porosity, tunable surface functionality, and remarkable adsorption capacity. Efficient dye removal can be achieved at relatively low dosages, minimizing the amount of adsorbent required and reducing secondary waste generation. Its ability to operate effectively under acidic conditions makes it suitable for real industrial effluents, which often exhibit variable pH and complex compositions. Moreover, the demonstrated reusability across multiple adsorption–desorption cycles enhance cost-effectiveness and reduces the environmental footprint of MOF-based treatment systems.

Together, these findings underscore the potential of integrating algae and MOFs as sustainable, efficient, and environmentally friendly tools for industrial wastewater treatment, supporting both ecological health and circular bioeconomy principles.

### Future work

#### Algal biomass

1. **Scale-Up Studies**: Future research should focus on scaling up the application of dried Ulva fasciata and Pterocladia capillacea biomass in real industrial wastewater treatment to validate efficiency under complex, multi-pollutant conditions.
2. **Continuous Flow and Column Experiments**: Long-term continuous flow systems should be implemented to simulate practical operational environments.
3. **Regeneration and Reusability**: Investigating biomass regeneration and reuse is essential for assessing economic feasibility and sustainability.
4. **Mechanistic Studies**: Molecular-level studies are needed to better understand adsorption mechanisms and surface interactions involved in dye binding.
5. **Biological Enhancement**: Exploring genetic or biochemical modification of algal biomass may enhance biosorption capacity and improve dye removal performance.

#### Metal–organic frameworks (UiO-66-NH₂)

1. **Framework Modification and Functionalization**: Future research should focus on surface modification and heteroatom incorporation to enhance dye selectivity, adsorption capacity, and chemical stability under varying pH and salinity conditions.
2. **Composite and Hybrid Materials**: Developing MOF-based composites with biopolymers, nanoparticles, or carbonaceous materials can improve recyclability, mechanical strength, and anti-fouling properties.
3. **Long-Term Stability and Regeneration**: Detailed studies beyond three adsorption–desorption cycles are needed, optimizing regeneration protocols and employing green solvents for desorption.
4. **Kinetic and Mechanistic Studies**: Advanced spectroscopic techniques and computational modeling should be applied to clarify adsorption pathways, competitive interactions with co-existing pollutants, and structure–performance relationships.
5. **Scale-Up and Pilot Applications**: Translating laboratory findings into pilot- and industrial-scale systems is crucial, particularly for real textile effluents containing mixed dyes, salts, and organic matter.
6. **Life Cycle Assessment (LCA)**: Comprehensive environmental and economic evaluations are required to assess the sustainability, cost-effectiveness, and overall environmental footprint of large-scale MOF applications.

## Supplementary Information

Below is the link to the electronic supplementary material.

Supplementary Material 1

## Data Availability

No datasets were generated or analysed during the current study.
