# Supplementary material for: Sustainable dye removal from industrial wastewater using marine algae-derived biosorbents and MOF-based hybrid composites
Source: Sci Rep. 2026 Mar 29;16:11349. doi: 10.1038/s41598-026-41983-5 (PMC13049084; doi:10.1038/s41598-026-41983-5)
Supplement: Supplementary file 1 — Supplementary Material 1 [file 41598_2026_41983_MOESM1_ESM.pdf]

Supplementary Information

Supplementary figures and tables associated with the manuscript entitled: 'Sustainable Dye Removal from Industrial Wastewater Using Marine Algae-Derived Biosorbents and MOF-Based Hybrid Composites'.

Figure S1

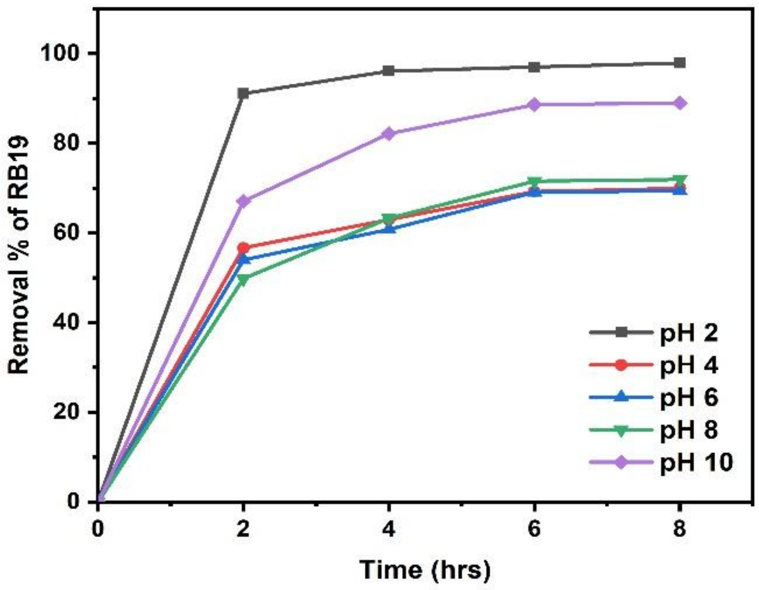

Figure S2

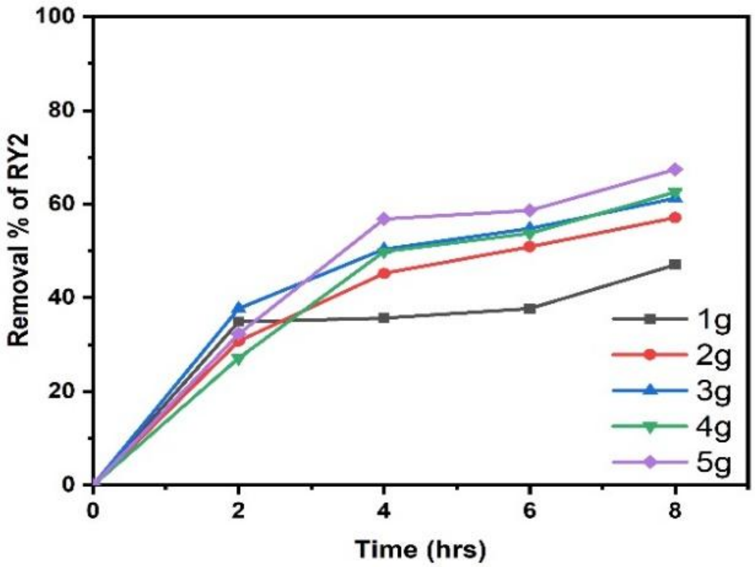

Supplementary data associated with the manuscript entitled 'Sustainable Dye Removal from Industrial Wastewater Using Marine Algae-Derived Biosorbents and MOF-Based Hybrid Composites'.

Figure S3

Concentration (ppm)	Exp.	Pseudo first order model			Pseudo second order model		
	Qexp	qcal	K1(1/min)	**R^2**	**q_{cal}**	**K_2(g/mg.min)**	**R^2**
0.15 (blue)	0.19	0.6458011	-0.0001151	**0.99576**	**6.489**	**0.154099**	**0.98461**
1.43 (red)	3.78	0.9094035	-0.00007188	**0.99054**	**113.8952**	**0.00179**	**0.95157**
2.76 (yellow)	4.95	0.5438138	-0.00008861	**0.97721**	**53.7923**	**0.0035**	**0.98511**

Sustainable Dye Removal from Industrial Wastewater Using Marine Algae-Derived Biosorbents and MOF-Based Hybrid Composites

Figure S4

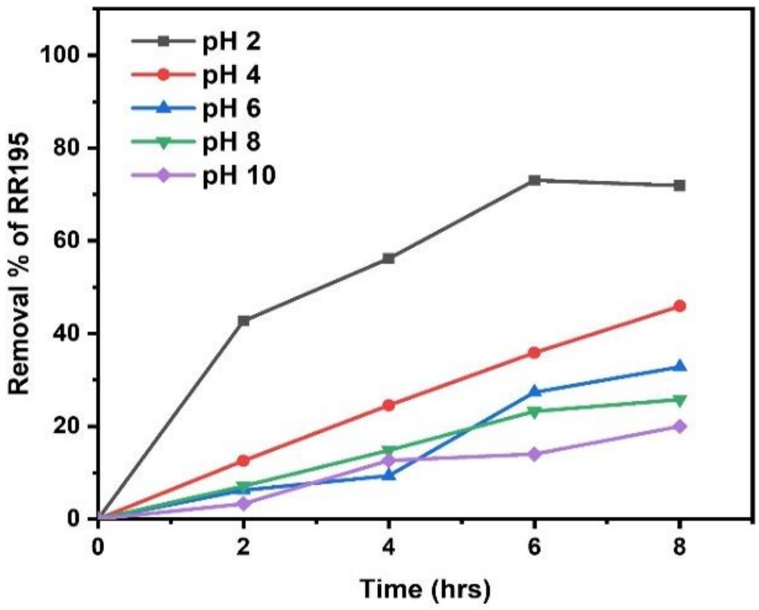

Table S1. Effect of Different fresh algal biomass of U. fasciata on Removal % of three Reactive Dyes.

Figure S5

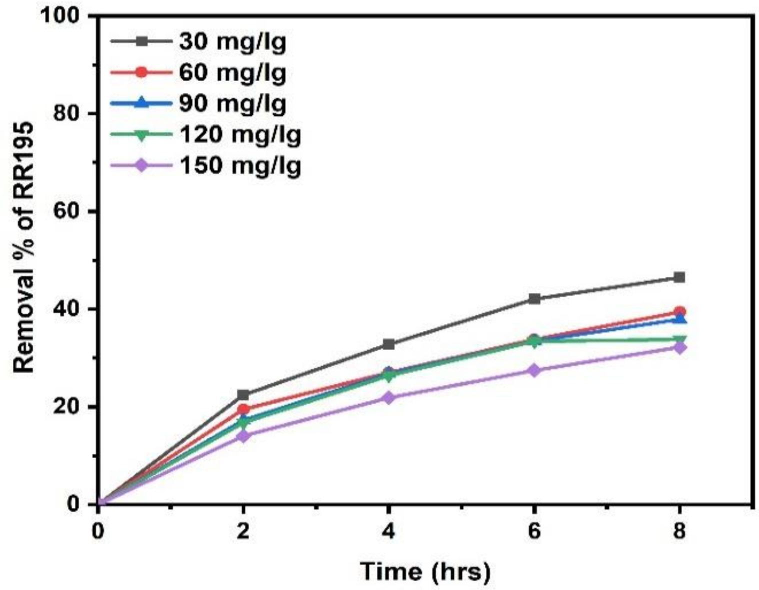

Table S2. Effect of Different fresh algal biomass of Pterocladia capillacea on Removal % of three Reactive Dyes.

Figure S6

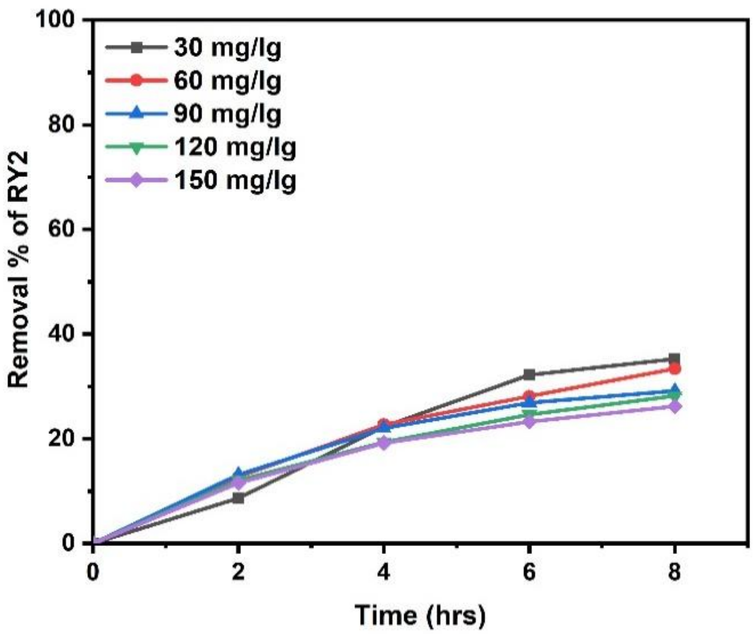

LSD: Least significant difference at 0.05. * : Statistically significant at $p \leq 0.05$. ** : Statistically significant at $p \leq 0.01$.

Figure S7

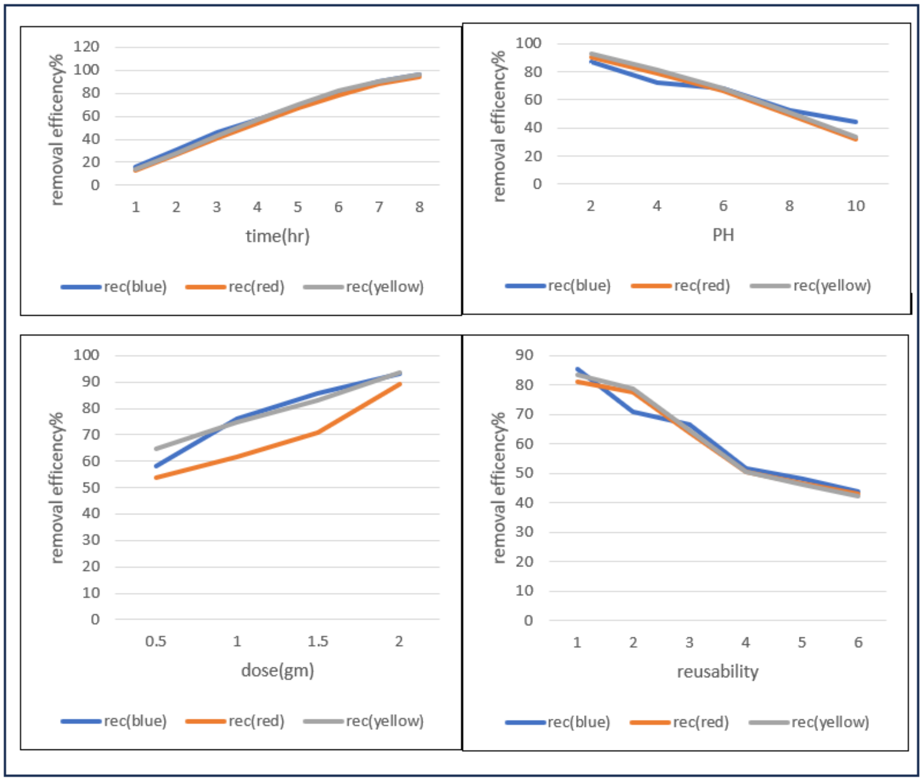

Different subscripts are significant. Data are expressed in mean ± SD.

Figure S8

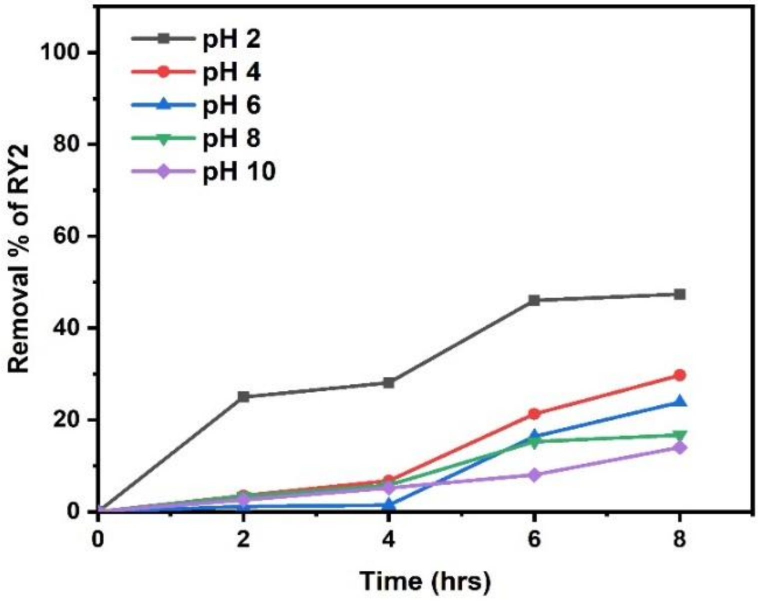

Table S3. Effect of Different dried algal biomass of Ulva fasciata on Removal % of three Reactive Dyes.

Figure S9

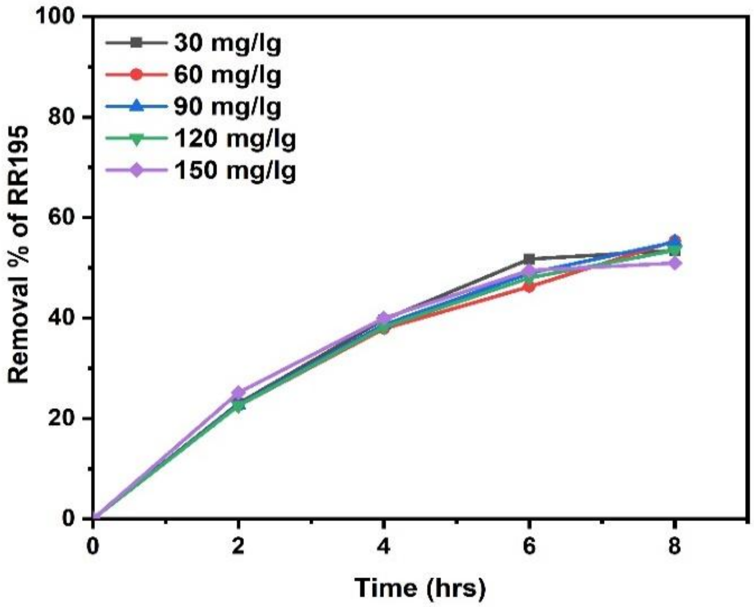

Table S4. Effect of Different dried algal biomass of Pterocladia capillacea on Removal % of three Reactive Dyes.

Figure S10

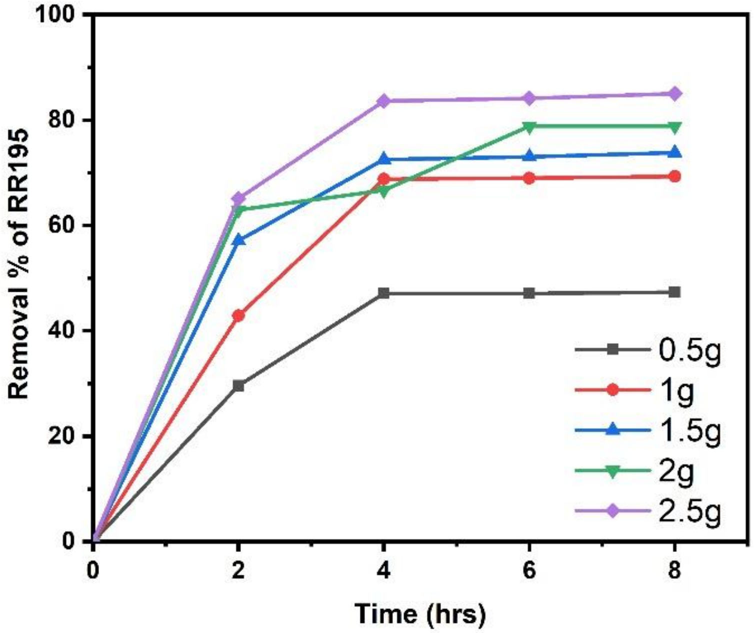

LSD: Least significant difference at 0.05. * : Statistically significant at p ≤ 0.05. ** : Statistically significant at p ≤ 0.01.

Figure S11

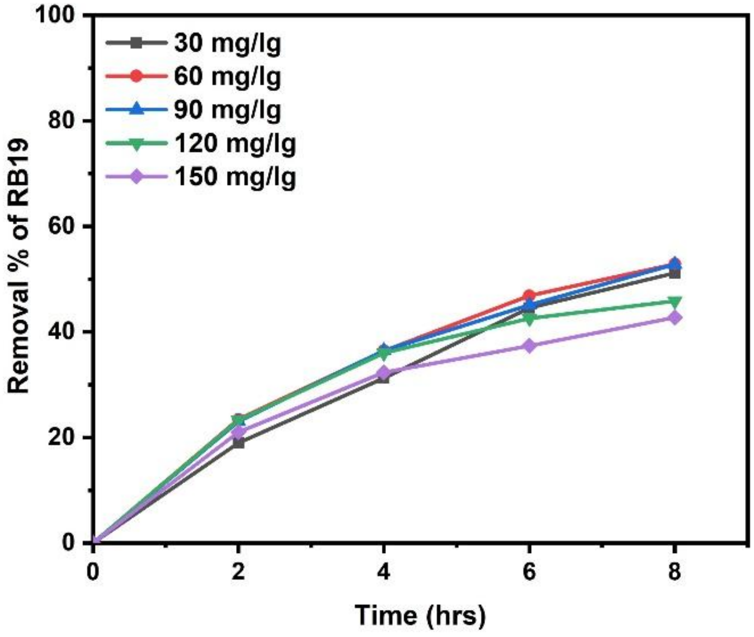

Different subscripts are significant.   Data are expressed in mean ± SD.

Figure S12

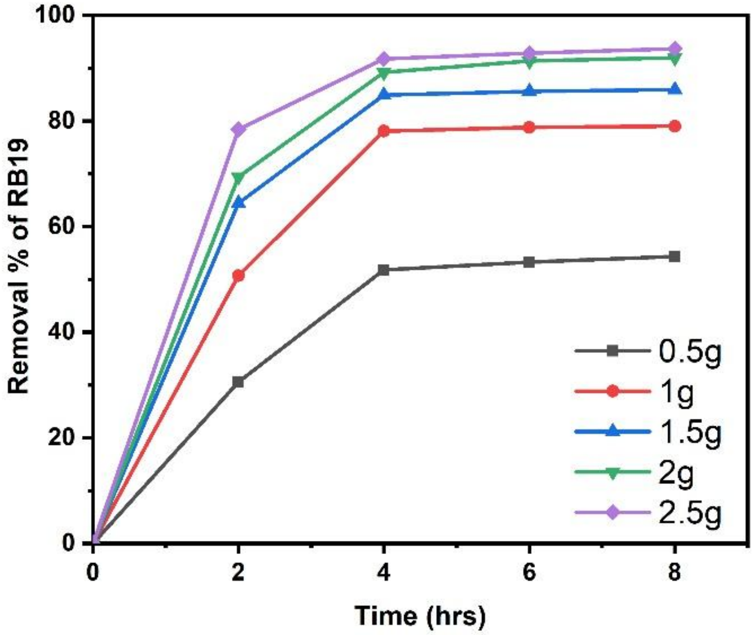

Figure S1. Characterization of MOF (a-FTIR, b-TGA, c-SEM, d-XRD)

Figure S13

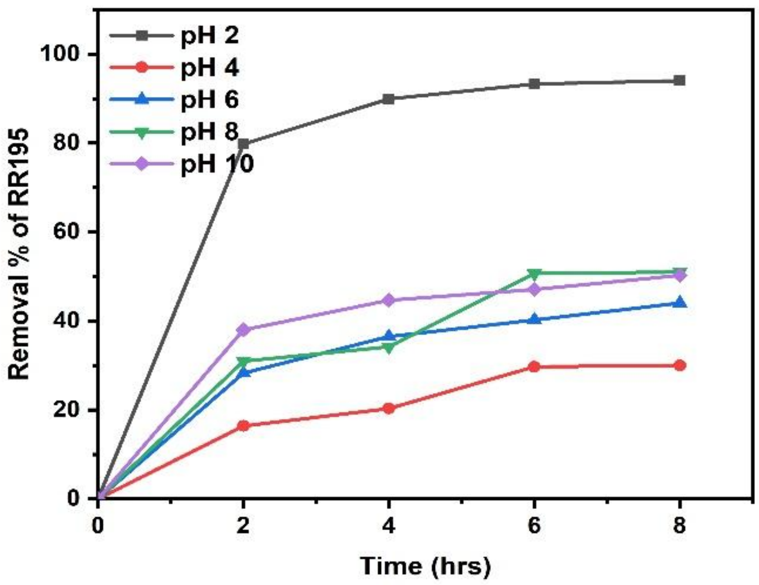

Table S5. Effect of Different Initial Dye Concentrations (mg/L) on the Removal % of three Reactive Dyes by fresh Ulva fasciata.

Figure S14

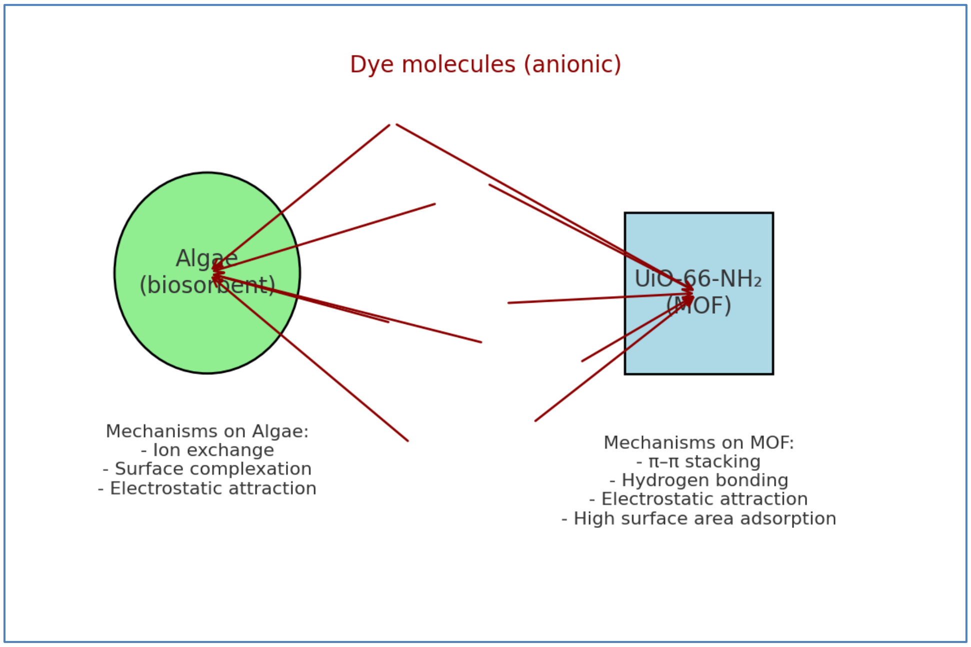

LSD: Least significant difference at 0.05. * : Statistically significant at $p \leq 0.05$. ** : Statistically significant at $p \leq 0.01$.

Figure S15

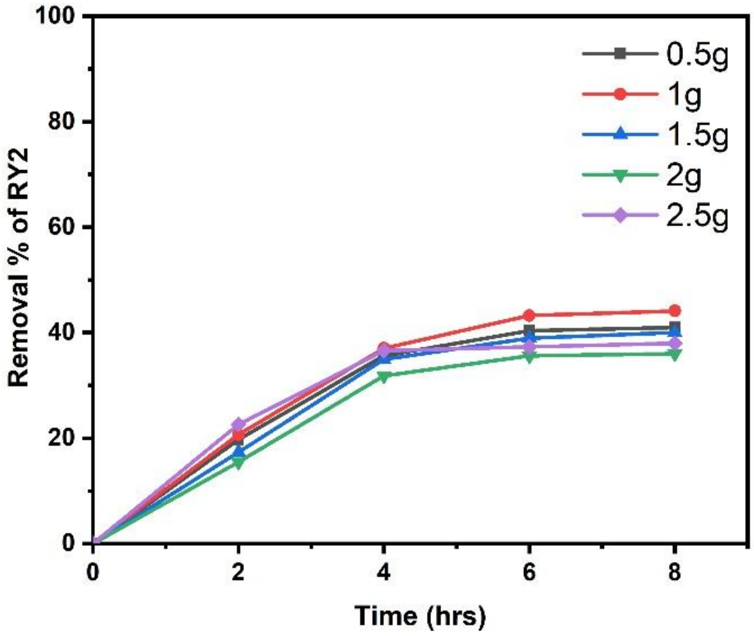

Different subscripts are significant.   Data are expressed in mean ± SD.

Figure S16

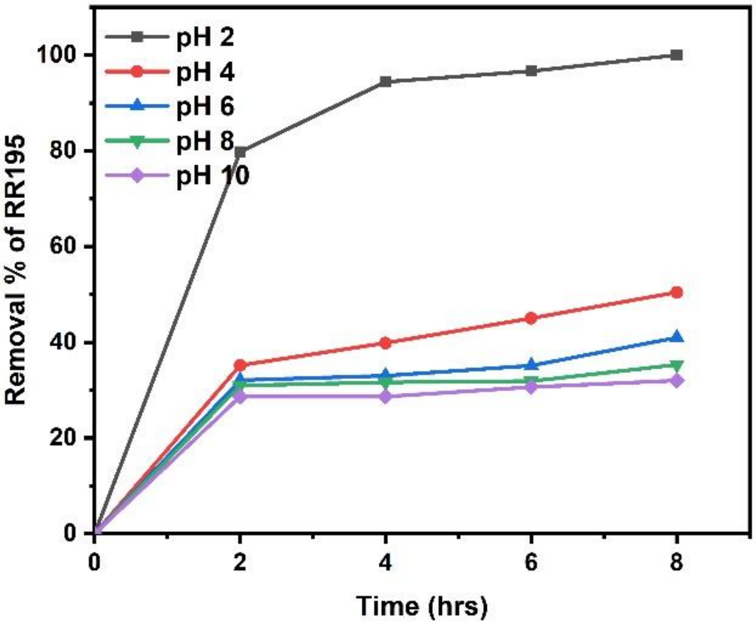

Table S6. Effect of Different Initial Dye Concentrations (mg/L) on the Removal % of three Reactive Dyes by fresh Pterocladia capillacea.

Figure S17

$$\frac{t}{q_t} = \frac{1}{k_2 q_e^2} + \frac{t}{q_e}$$

LSD: Least significant difference at 0.05.   * : Statistically significant at $p \leq 0.05$.   ** : Statistically significant at $p \leq 0.01$.

Figure S18

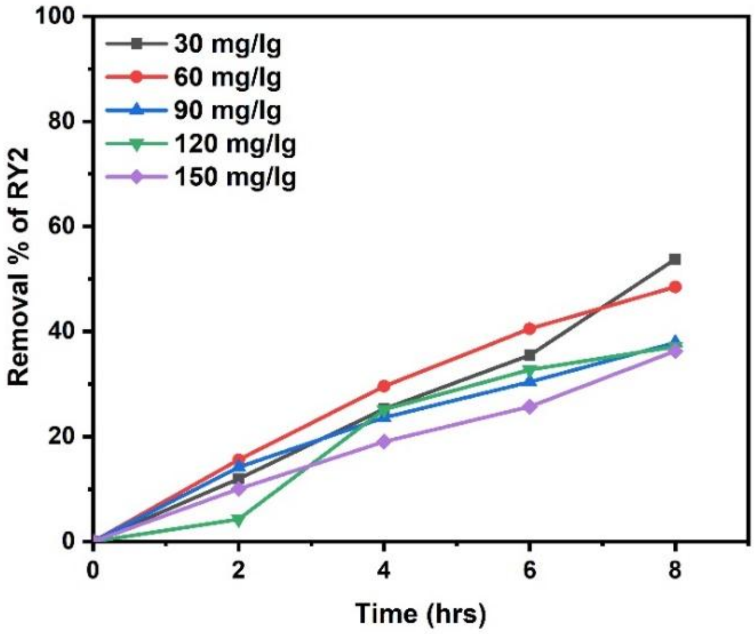

Different subscripts are significant. Data are expressed in mean ± SD.

Figure S19

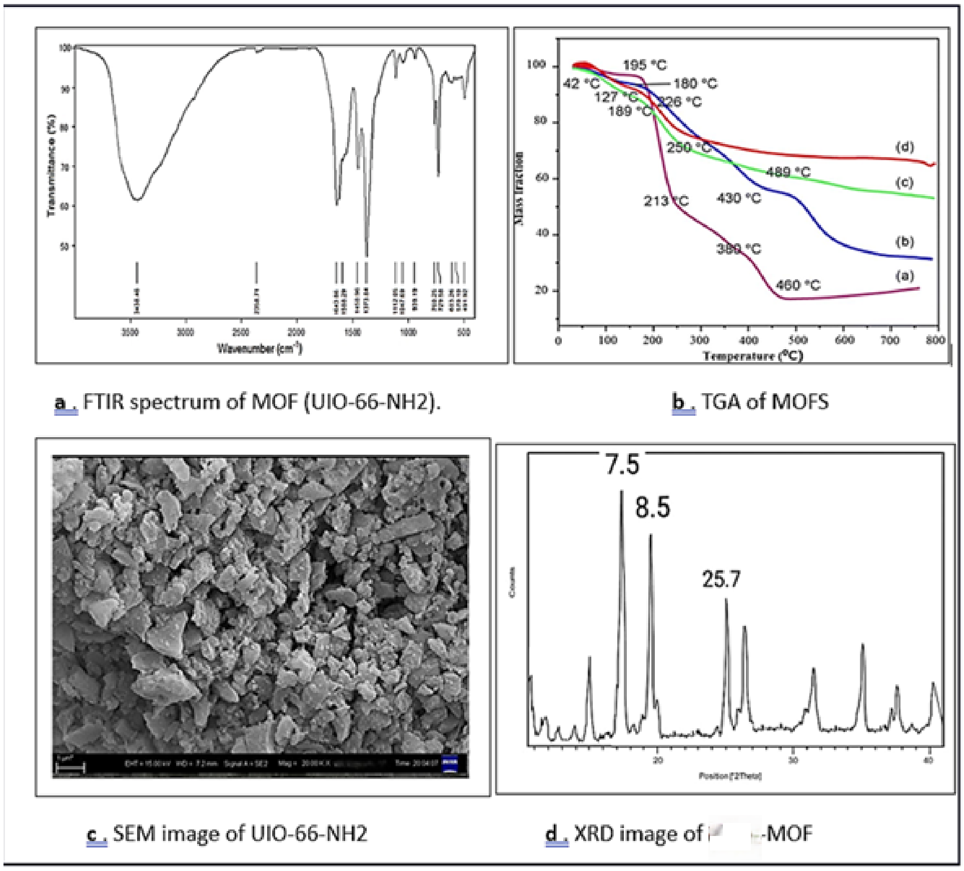

a. FTIR spectrum of MOF (UIO-66-NH2).

b. TGA of MOFS

c. SEM image of UIO-66-NH2

d. XRD image of [illegible]-MOF

Table S7. Effect of Different Initial Dye Concentrations (mg/L) on the Removal % of three Reactive Dyes by dried Ulva fasciata.

Figure S20

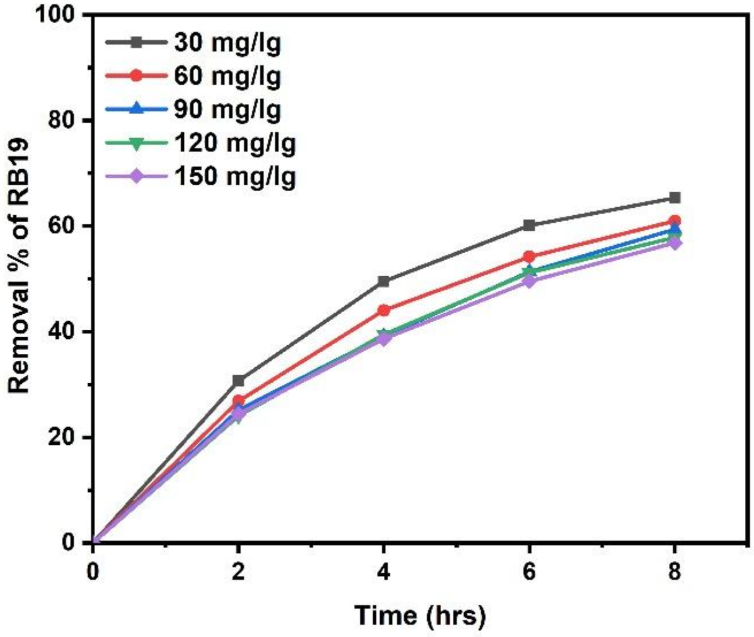

LSD: Least significant difference at 0.05. * : Statistically significant at p ≤ 0.05. ** : Statistically significant at p ≤ 0.01.

Figure S21

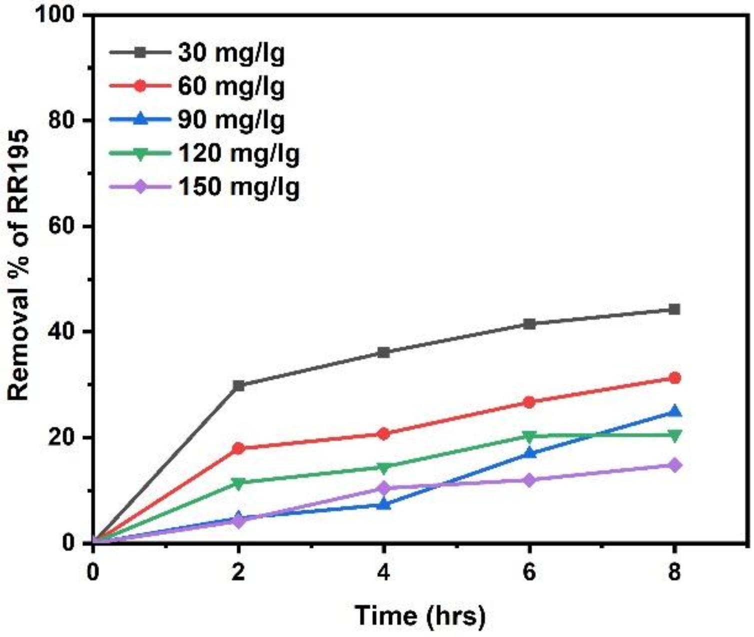

Different subscripts are significant. Data are expressed in mean ± SD.

Figure S22

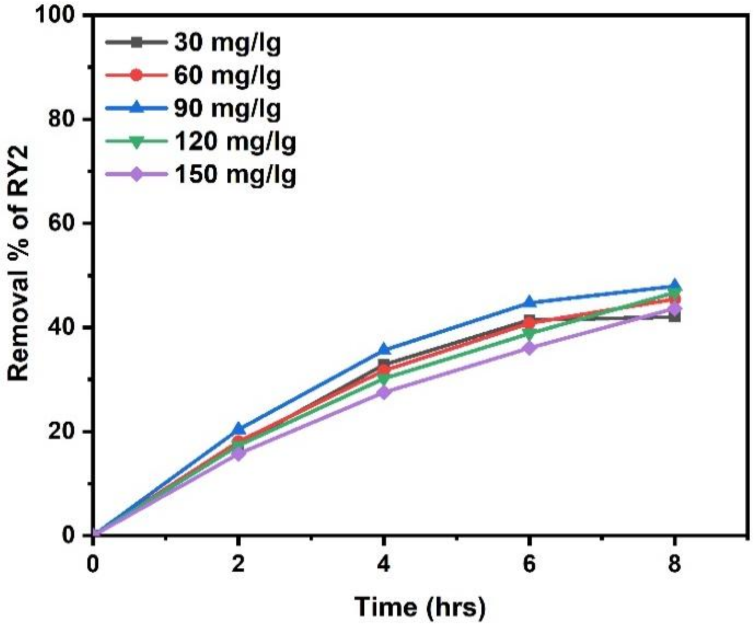

Table S8. Effect of Different Initial Dye Concentrations (mg/L) on the Removal % of three Reactive Dyes by dried Pterocladia capillacea.

Figure S23

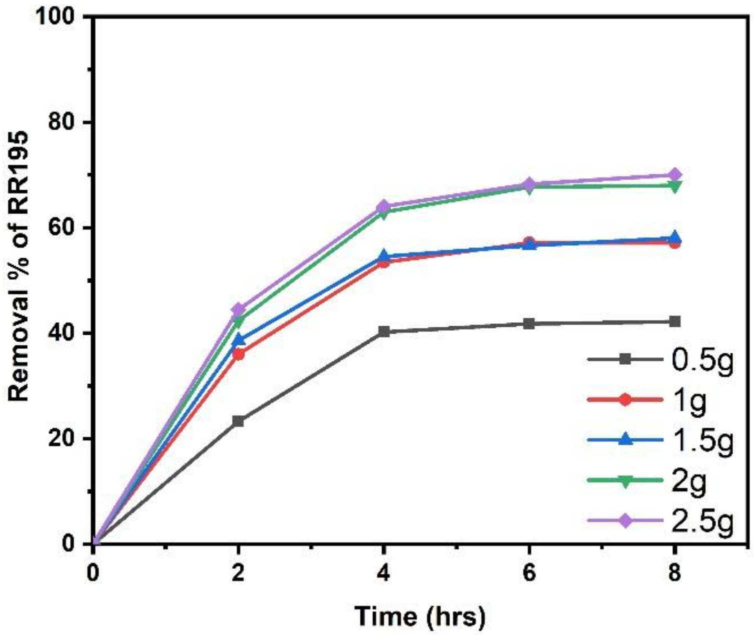

LSD: Least significant difference at 0.05. * : Statistically significant at $p \leq 0.05$. ** : Statistically significant at $p \leq 0.01$.

Figure S24

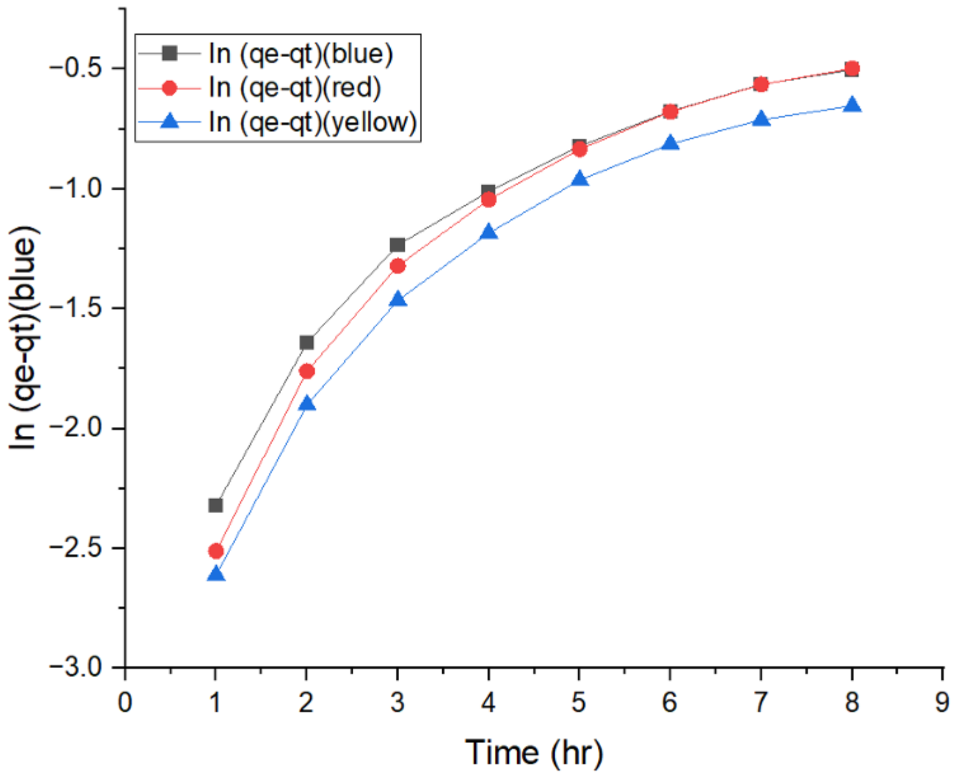

Different subscripts are significant.   Data are expressed in mean ± SD.

Figure S25

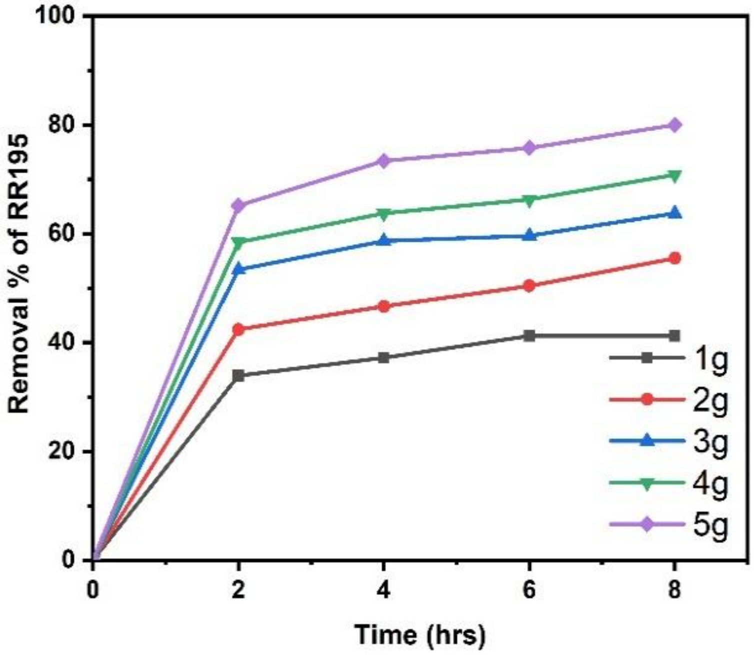

Table S9. Effect of Different pHs on Removal % of three Reactive Dyes using fresh algal biomass of U. fasciata.

Figure S26

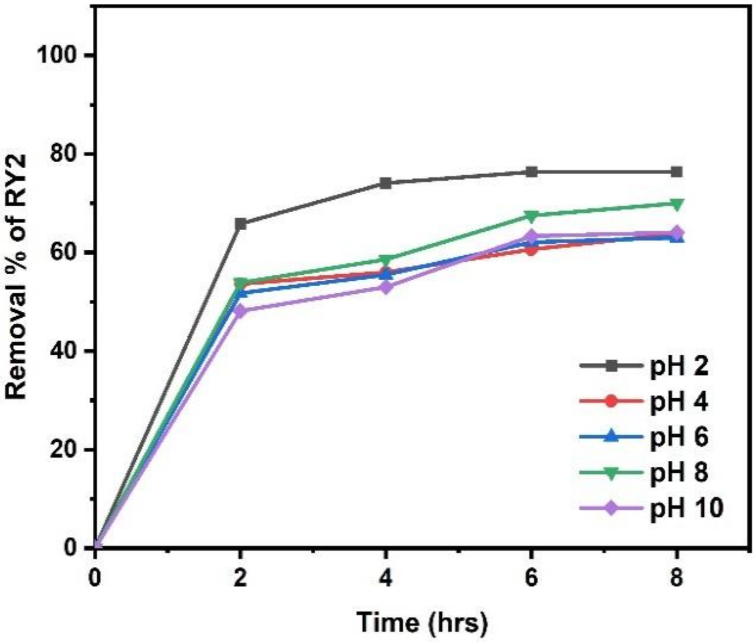

LSD: Least significant difference at 0.05. * : Statistically significant at $p \leq 0.05$. ** : Statistically significant at $p \leq 0.01$.

Figure S27

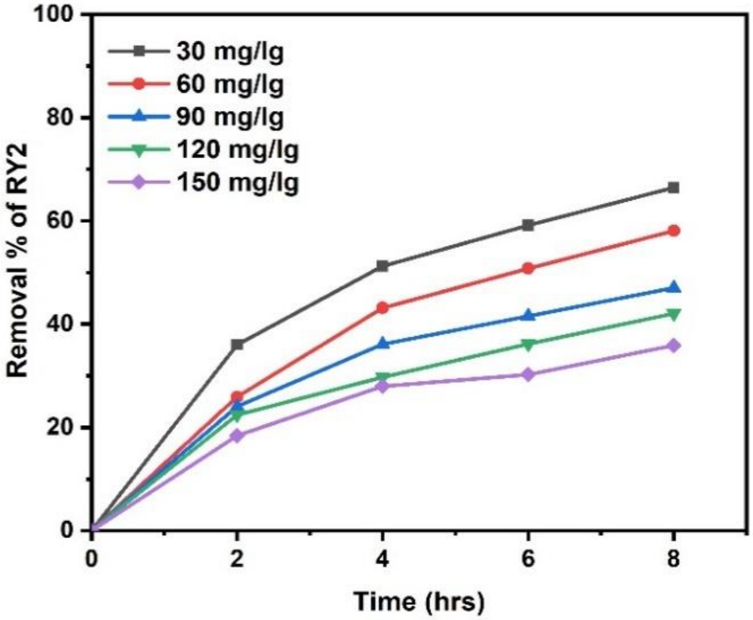

Different subscripts are significant. Data are expressed in mean ± SD.

Figure S28

Concentration, ppm	equation	R^2
0.15 (blue)	Y=-0.01854x +0.1541	R^2=0.98461
1.43 (red)	Y=-0.00878x +0.04307	R^2=0.95157
2.76 (yellow)	Y=-0.01859x +0.09848	R^2=0.98511

Table S10. Effect of Different pHs on Removal % of three Reactive Dyes using fresh algal biomass of P. capillacea.

Figure S29

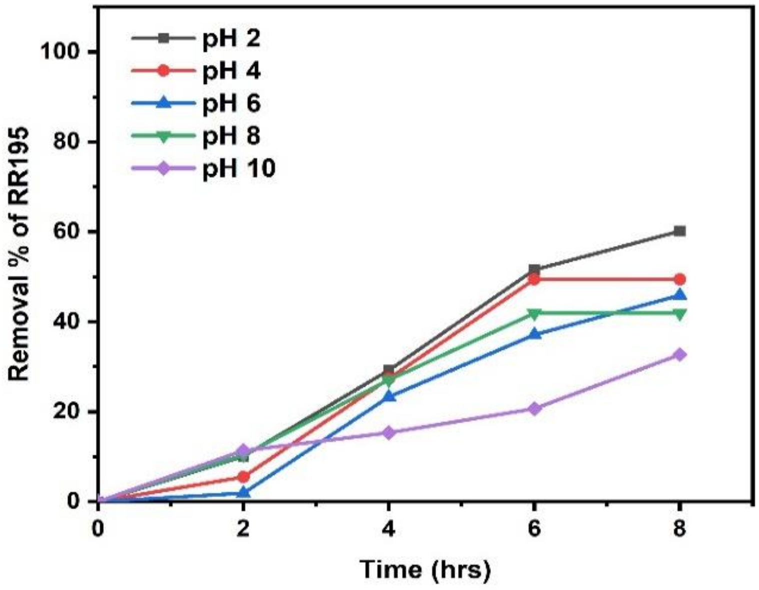

LSD: Least significant difference at 0.05. * : Statistically significant at $p \le 0.05$. ** : Statistically significant at $p \le 0.01$.

Figure S30

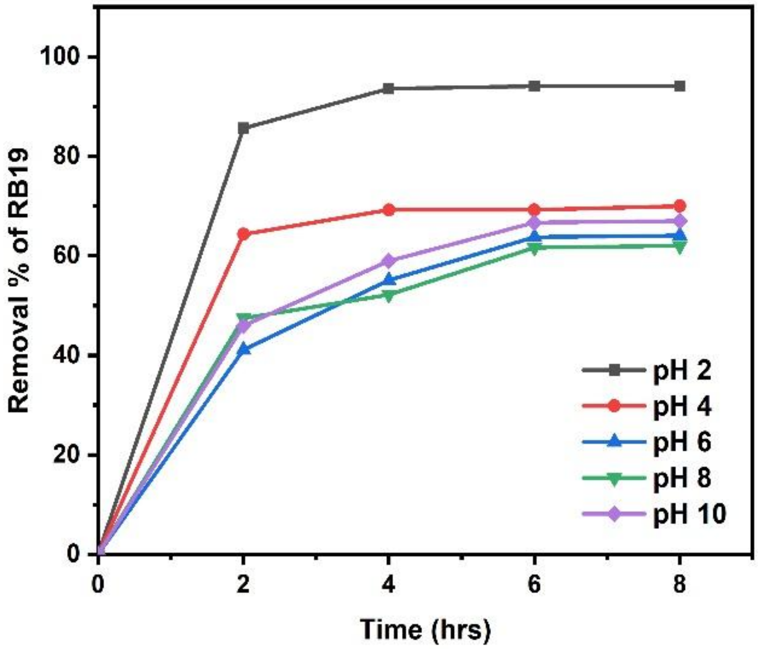

Different subscripts are significant. Data are expressed in mean ± SD.

Figure S31

Plain Text

```
Kinetic Model Parameters and R-squared values (Fresh Ulva fasciata):
RY2:
  Pseudo-First-Order: Re = 67.82, k1 = 0.8278, R-squared = 0.6819
  Pseudo-Second-Order: Re = 75.36, k2 = 0.0183, R-squared = 0.8242
RR195:
  Pseudo-First-Order: Re = 96.90, k1 = 1.1556, R-squared = 0.6281
  Pseudo-Second-Order: Re = 102.86, k2 = 0.0270, R-squared = 0.8230
RB19:
  Pseudo-First-Order: Re = 99.29, k1 = 1.3692, R-squared = 0.9808
  Pseudo-Second-Order: Re = 102.68, k2 = 0.0478, R-squared = 0.9754
RB5:
  Pseudo-First-Order: Re = 97.05, k1 = 1.3622, R-squared = 0.9998
  Pseudo-Second-Order: Re = 100.26, k2 = 0.0496, R-squared = 0.9328
```

Table S11. Effect of Different pHs on Removal % of three Reactive Dyes using dried algal biomass of Ulva fasciata.

Figure S32

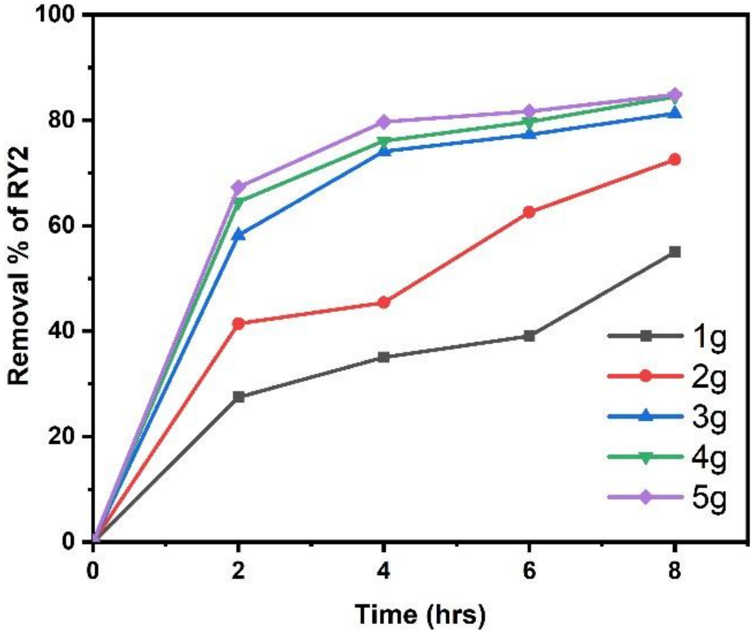

LSD: Least significant difference at 0.05. * : Statistically significant at $p \leq 0.05$. ** : Statistically significant at $p \leq 0.01$.

Figure S33

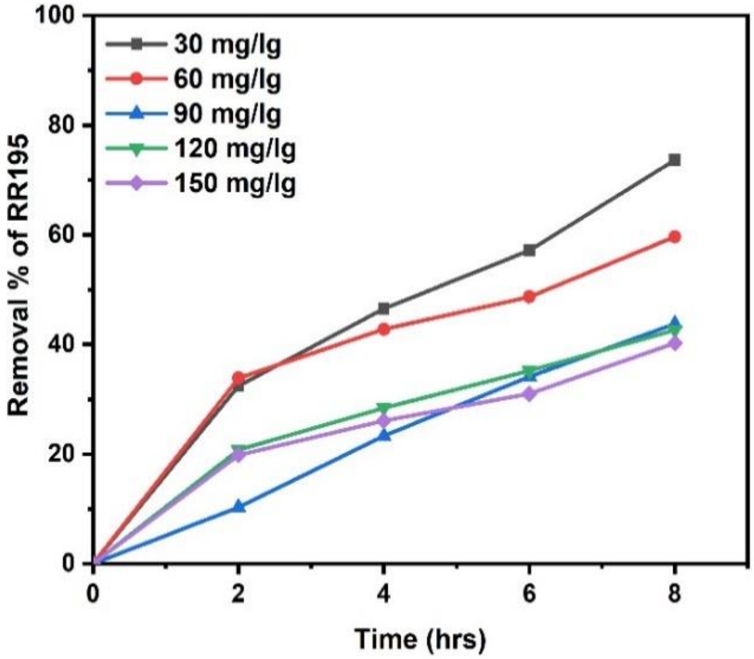

Different subscripts are significant. Data are expressed in mean ± SD.

Figure S34

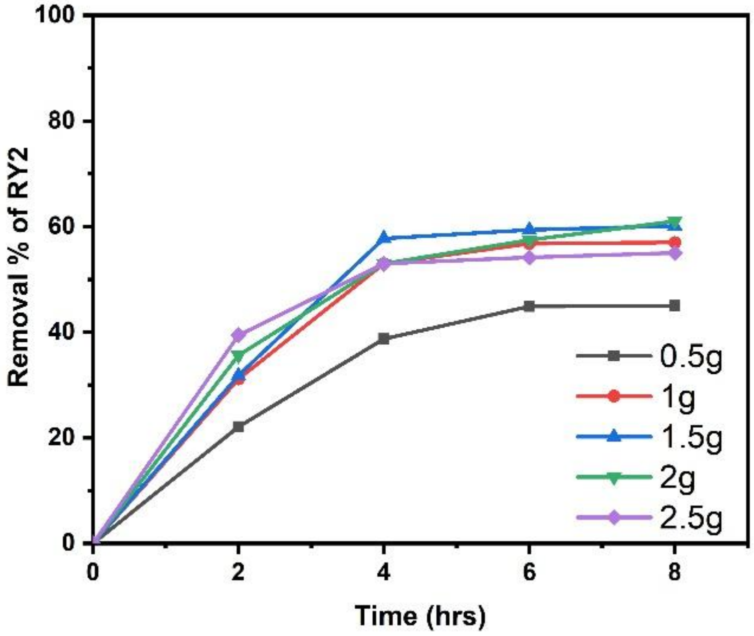

Table S12. Effect of Different pHs on Removal % of three Reactive Dyes using dried algal biomass of Pterocladia capillacea.

Figure S35

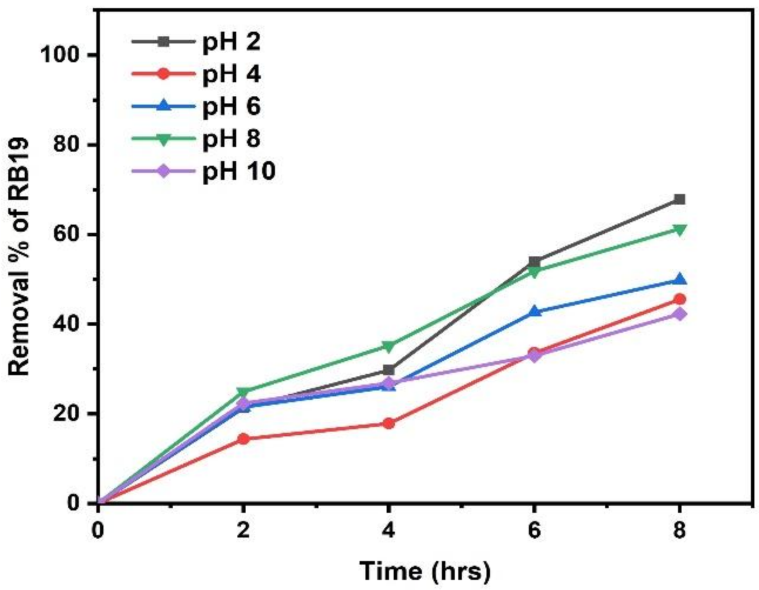

LSD: Least significant difference at 0.05. * : Statistically significant at $p \le 0.05$. ** : Statistically significant at $p \le 0.01$.

Figure S36

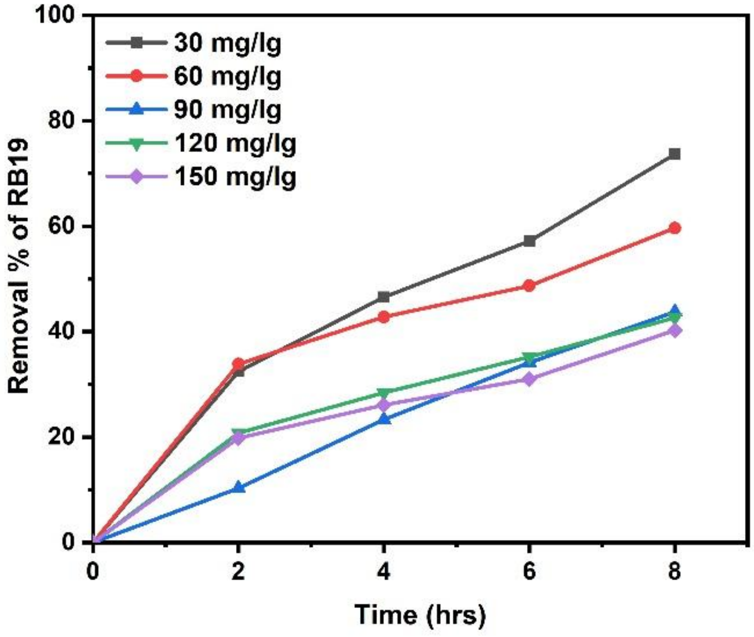

Different subscripts are significant.   Data are expressed in mean ± SD.

Figure S37

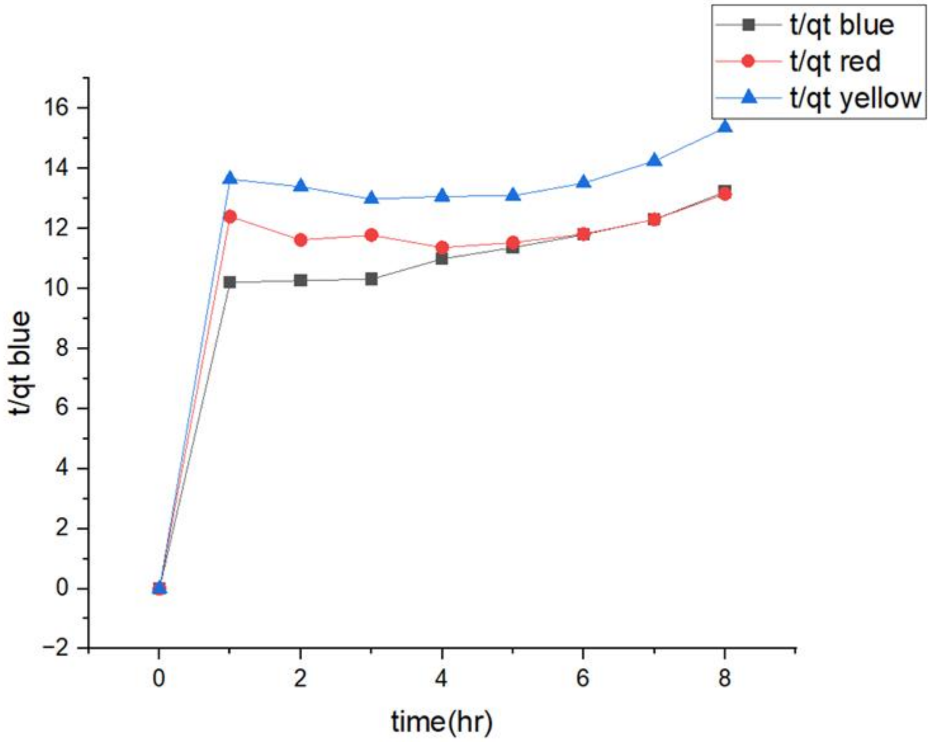

Figure S2.

Figure S38

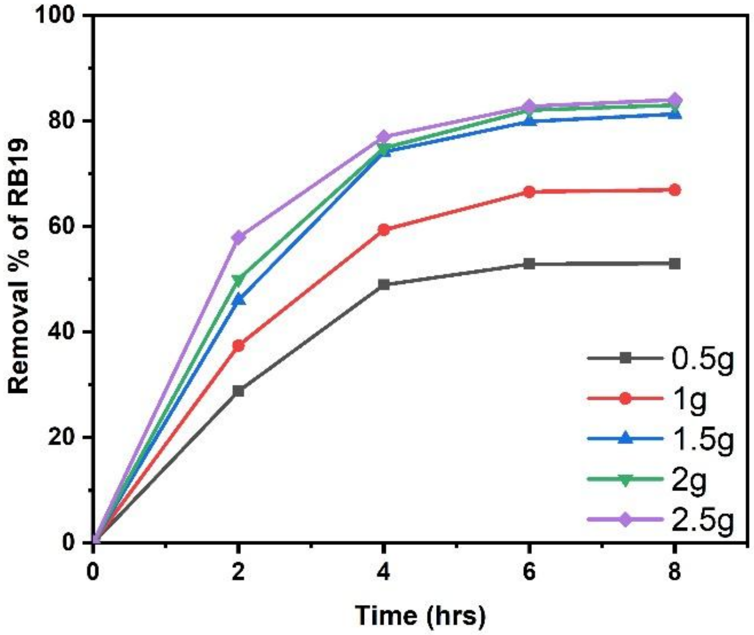

Adsorption kinetics

Figure S39

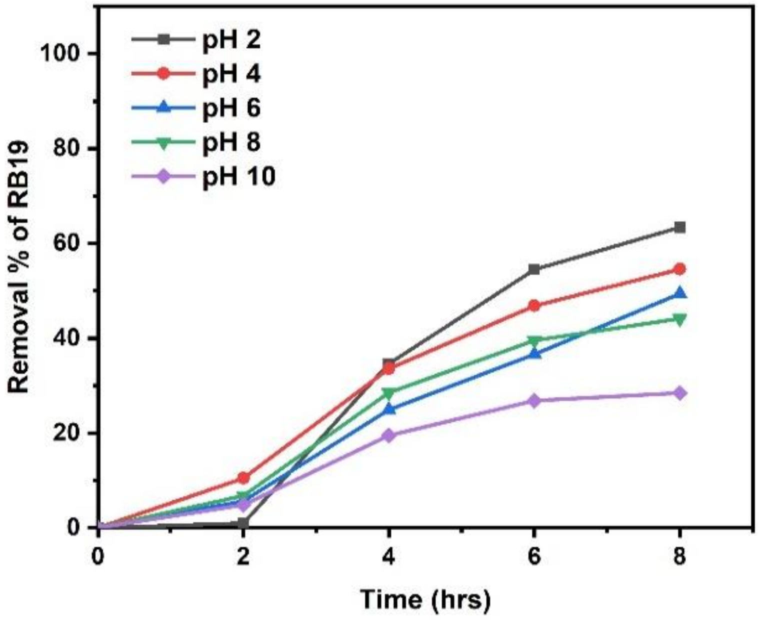

Illustrates the relationship between reactive dyes (yellow, red, and blue) pollutant ions adsorbed on both two tested algal species and the nanoparticle MOF with time. Adsorption process takes place through solution/solid interface then pollutants diffuse in the solution fluid film then contaminants move towards the active site in the nanomaterial then to the internal pores of the nanomaterial through intraparticle diffusion process followed by finally adsorption takes place of the pollutants at the adsorption sites [48].Pseudo-first-order reactions Pseudo-first-order kinetics was applied to study the adsorption rate of reactive dyes on nanoparticles MOF at equilibrium. it undergoes a physical adsorption process [49-51]. Equation – represents pseudo-first-order reaction as follows:

Figure S40

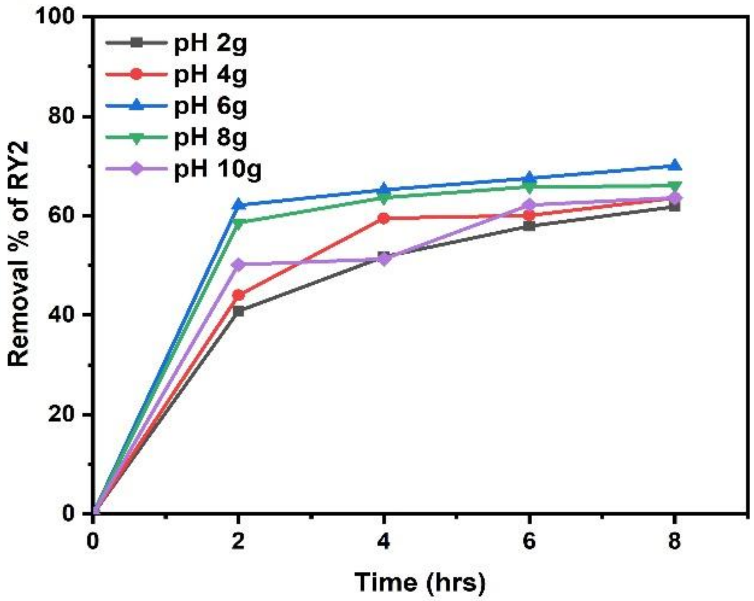

$$\ln (q_e - q_t) = \ln(q_e) - k_1 * t$$

Figure S41

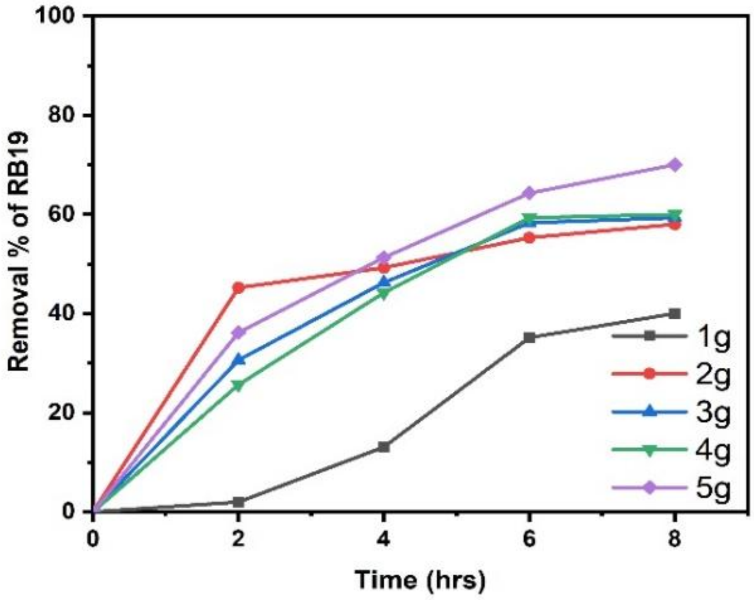

Where, qe is the quantity adsorbed at equilibrium (mg/g) and qt is the quantity absorbed at time t (mg/g). k1 is the rate constant for the pseudo-first-order sorption (min−1). The slope of log (qe - qt) vs. t should be a straight line if the adsorption reaction is first order. Pseudo-second order reactions Pseudo-second order kinetics was applied to study the rate of reactive dyes removal at equilibrium. It undergoes a chemical adsorption process. 2nd eq sup. Data

Figure S42

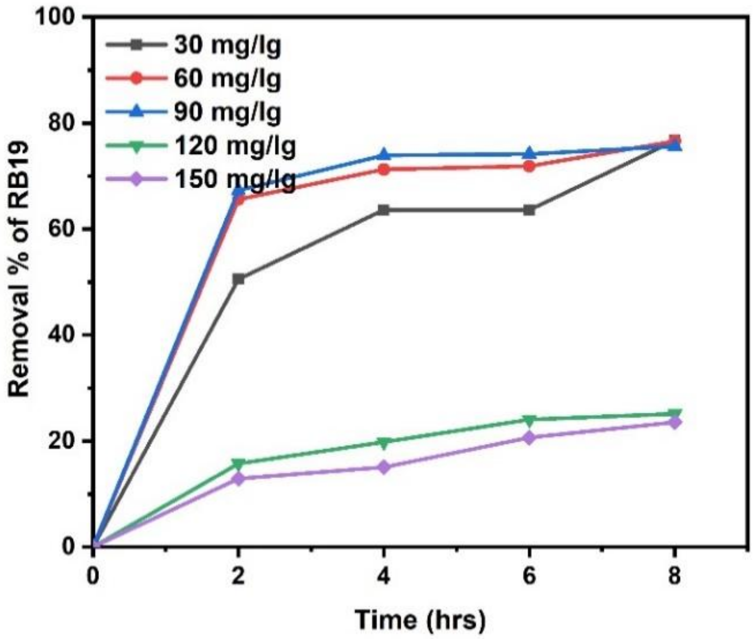

Table S13. The following graph illustrates the experimental data points and the fitted pseudo-first order and pseudo-second-order kinetic models for each dye by using two marine green algae.

Figure S43

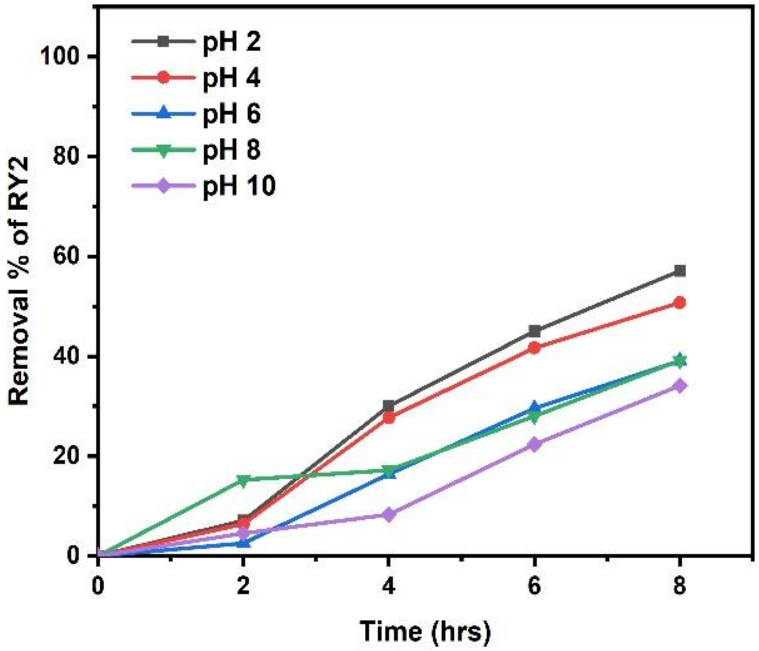

Figure S3. Figure (9): The following graph illustrates the experimental data points and the fitted pseudo-first order and pseudo-second-order kinetic models for each dye by using green algae

Figure S44

Concentration, ppm	Equation	R^2
**0.15 (blue)**	**Y=-0.02073x +4.70424**	**R^2=0.99576**
**1.43 (red)**	**Y=-0.01294x +3.00303**	**R^2=0.99054**
**2.76 (yellow)**	**Y=-0.01595x +6.28939**	**R^2=0.977721**

Table S14. Adsorption Kinetic parameters of Pseudo first & Second order for dyes adsorption:

Figure S45

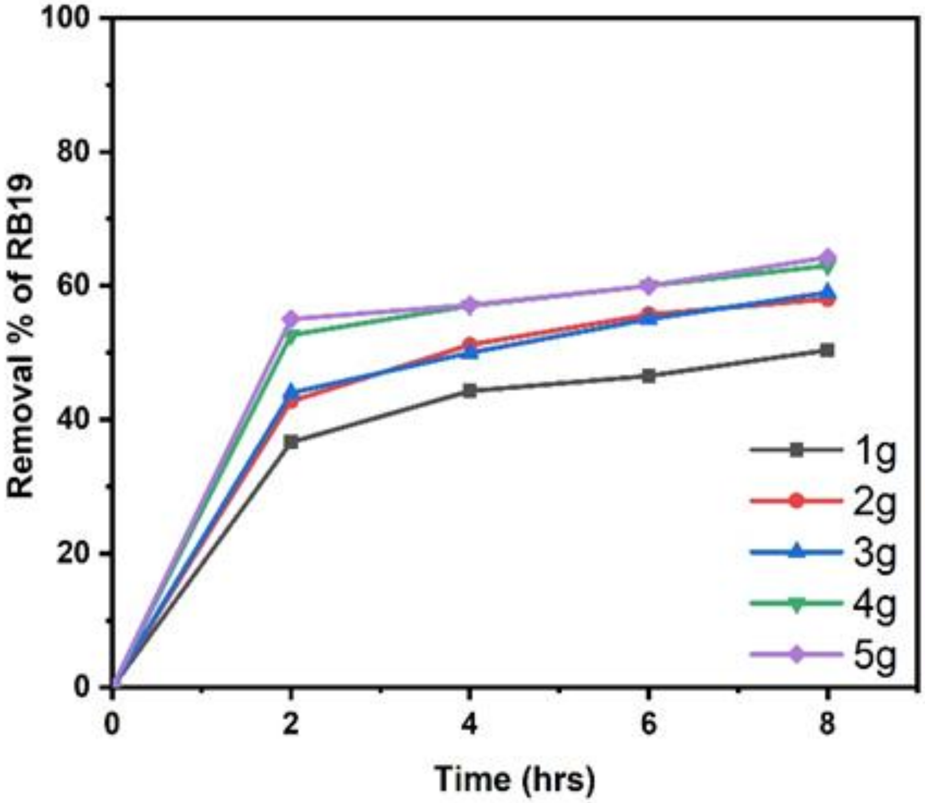

Table S15. Pseudo-First Order Adsorption Kinetic reactions.

Figure S46

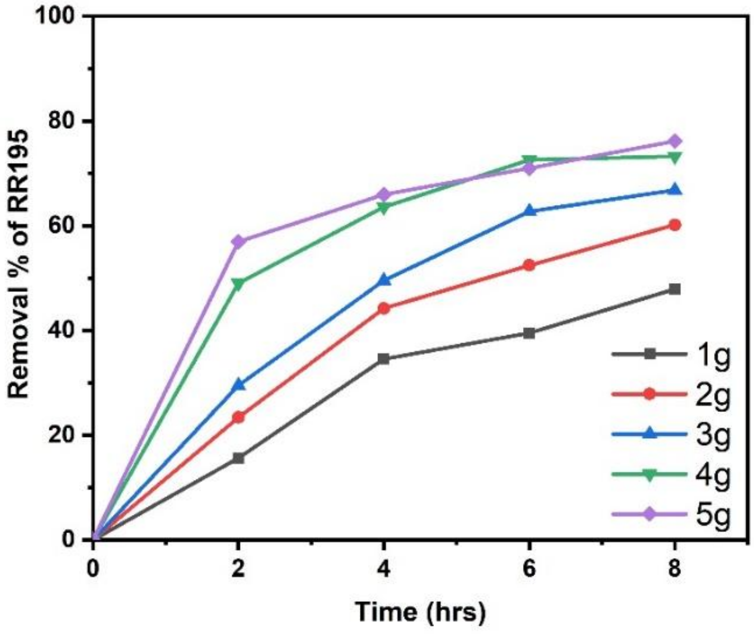

Table S16. Pseudo-Second order Adsorption kinetic reactions.

Figure S47

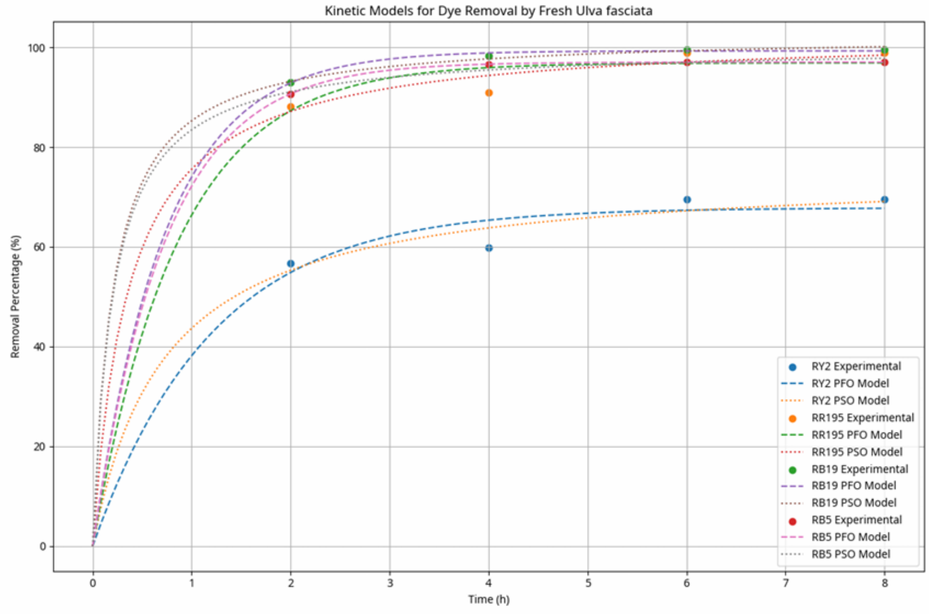

Figure S4. Figure (10): First-Second order reactions Plot for the adsorption process

Table S1

Time (hr)	Type of Dye	1 g	2 g	3 g	4 g	5 g	F (p)	LSD
0	RY2 (λmax = 404 nm)	0.00 ± 0.00[a]	0.00 ± 0.00[b]	0.00 ± 0.00[c]	0.00 ± 0.00[d]	0.00 ± 0.00[e]	1.574 (0.212)	0.25
2	RY2 (λmax = 404 nm)	27.49 ± 0.27[e]	41.43 ± 0.41[d]	58.17 ± 0.58[c]	64.54 ± 0.65[b]	67.33 ± 0.67[a]	4.350* (0.010)	0.30
4	RY2 (λmax = 404 nm)	35.06 ± 0.35[e]	45.42 ± 0.45[d]	74.10 ± 0.74[c]	76.10 ± 0.76[b]	79.68 ± 0.80[a]	4.282* (0.011)	0.32
6	RY2 (λmax = 404 nm)	39.04 ± 0.39[e]	62.55 ± 0.63[d]	77.29 ± 0.77[c]	79.68 ± 0.80[b]	81.67 ± 0.82[a]	4.210* (0.012)	0.35
8	RY2 (λmax = 404 nm)	54.98 ± 0.55[e]	72.51 ± 0.73[d]	81.27 ± 0.81[c]	84.46 ± 0.84[b]	84.86 ± 0.85[a]	4.210* (0.012)	0.36
0	RR195 (λmax = 540 nm)	0.00 ± 0.00[a]	0.00 ± 0.00[b]	0.00 ± 0.00[c]	0.00 ± 0.00[d]	0.00 ± 0.00[e]	2.225 (0.090)	0.25
2	RR195 (λmax = 540 nm)	33.92 ± 0.34[e]	42.40 ± 0.42[d]	53.36 ± 0.53[c]	58.42 ± 0.58[b]	65.14 ± 0.65[a]	4.510* (0.009)	0.30

4	RR195 (λmax = 540 nm)	37.22 ± 0.37[e]	46.64 ± 0.47[d]	58.66 ± 0.59[c]	63.72 ± 0.64[b]	73.38 ± 0.73[a]	4.510* (0.009)	0.32
6	RR195 (λmax = 540 nm)	41.22 ± 0.41[e]	50.41 ± 0.50[d]	59.60 ± 0.60[c]	66.31 ± 0.66[b]	75.74 ± 0.76[a]	4.510* (0.009)	0.34
8	RR195 (λmax = 540 nm)	41.22 ± 0.41[e]	55.48 ± 0.55[d]	63.72 ± 0.64[c]	70.79 ± 0.71[b]	79.98 ± 0.80[a]	4.510* (0.009)	0.36
0	RB19 (λmax = 594 nm)	0.00 ± 0.00[a]	0.00 ± 0.00[b]	0.00 ± 0.00[c]	0.00 ± 0.00[d]	0.00 ± 0.00[e]	1.660 (0.199)	0.25
2	RB19 (λmax = 594 nm)	2.01 ± 0.02[e]	45.23 ± 0.45[a]	30.65 ± 0.31[c]	25.63 ± 0.26[d]	36.18 ± 0.36[b]	4.510* (0.009)	0.30
4	RB19 (λmax = 594 nm)	13.07 ± 0.13[e]	49.25 ± 0.49[b]	46.23 ± 0.46[c]	44.22 ± 0.44[d]	51.26 ± 0.51[a]	4.282* (0.012)	0.32
6	RB19 (λmax = 594 nm)	35.18 ± 0.35[e]	55.28 ± 0.55[d]	58.29 ± 0.58[c]	59.30 ± 0.59[b]	64.32 ± 0.64[a]	4.350* (0.010)	0.35
8	RB19 (λmax	40.00 ± 0.40[e]	58.00 ± 0.58[d]	59.30 ± 0.59[c]	60.00 ± 0.60[b]	70.00 ± 0.70[a]	4.282* (0.011)	0.36

= 594 nm)

Table S2

Time (hr)	Type of Dye	1 g	2 g	3 g	4 g	5 g	F (p)	LSD
0	RY2 (λmax = 404 nm)	0.00 ± 0.00[a]	0.00 ± 0.00[b]	0.00 ± 0.00[c]	0.00 ± 0.00[d]	0.00 ± 0.00[e]	1.215 (0.210)	0.25
2	RY2 (λmax = 404 nm)	34.88 ± 0.35[c]	30.75 ± 0.31[d]	37.73 ± 0.38[a]	27.13 ± 0.27[e]	32.30 ± 0.32[b]	4.350* (0.010)	0.30
4	RY2 (λmax = 404 nm)	35.66 ± 0.36[e]	45.22 ± 0.45[d]	50.39 ± 0.50[c]	49.87 ± 0.50[b]	56.85 ± 0.57[a]	4.282* (0.011)	0.32
6	RY2 (λmax = 404 nm)	37.73 ± 0.38[e]	50.90 ± 0.51[d]	54.78 ± 0.55[c]	53.75 ± 0.54[b]	58.66 ± 0.59[a]	4.350* (0.010)	0.34
8	RY2 (λmax = 404 nm)	47.03 ± 0.47[e]	57.11 ± 0.57[d]	61.24 ± 0.61[c]	62.53 ± 0.63[b]	67.44 ± 0.67[a]	4.210* (0.012)	0.36
0	RR195 (λmax = 540 nm)	0.00 ± 0.00[a]	0.00 ± 0.00[b]	0.00 ± 0.00[c]	0.00 ± 0.00[d]	0.00 ± 0.00[e]	1.188 (0.220)	0.25
2	RR195	15.65 ±	23.39 ±	29.52 ±	49.03 ±	56.94 ±	4.510*	0.30

	(λmax = 540 nm)	0.16[e]	0.23[d]	0.30[c]	0.49[b]	0.57[a]	(0.009)	
4	RR195 (λmax = 540 nm)	34.52 ± 0.35[e]	44.19 ± 0.44[d]	49.52 ± 0.50[c]	63.55 ± 0.64[b]	65.97 ± 0.66[a]	4.510* (0.009)	0.32
6	RR195 (λmax = 540 nm)	39.52 ± 0.40[e]	52.42 ± 0.52[d]	62.74 ± 0.63[c]	72.58 ± 0.73[a]	70.97 ± 0.71[b]	4.510* (0.009)	0.34
8	RR195 (λmax = 540 nm)	47.90 ± 0.48[e]	60.16 ± 0.60[d]	66.77 ± 0.67[c]	73.23 ± 0.73[b]	76.13 ± 0.76[a]	4.510* (0.009)	0.36
0	RB19 (λmax = 594 nm)	0.00 ± 0.00[a]	0.00 ± 0.00[b]	0.00 ± 0.00[c]	0.00 ± 0.00[d]	0.00 ± 0.00[e]	1.241 (0.200)	0.25
2	RB19 (λmax = 594 nm)	36.64 ± 0.37[e]	42.75 ± 0.43[d]	44.00 ± 0.44[c]	52.67 ± 0.53[b]	55.00 ± 0.55[a]	4.510* (0.009)	0.30
4	RB19 (λmax = 594 nm)	44.27 ± 0.44[e]	51.15 ± 0.51[d]	50.00 ± 0.50[c]	57.00 ± 0.57[b]	57.11 ± 0.57[a]	4.282* (0.010)	0.32
6	RB19 (λmax = 594 nm)	46.56 ± 0.47[e]	55.73 ± 0.56[d]	55.00 ± 0.55[c]	60.00 ± 0.60[b]	60.00 ± 0.60[a]	4.350* (0.010)	0.34

8	RB19 (λmax = 594 nm)	50.38 ± 0.50^{e}	58.02 ± 0.58^{d}	59.00 ± 0.59^{c}	63.00 ± 0.63^{b}	64.23 ± 0.64^{a}	4.282* (0.011)	0.36

Table S3

Figure 2. Effect of Different fresh algal biomass of both Ulva fasciata (a,b,c) and Pterocladia capillacea (d,e,f) on the removal of RY2, RR195, and RB19, respectively.

Figure 2. Effect of Different fresh algal biomass of both Ulva fasciata (a,b,c) and Pterocladia capillacea (d,e,f) on the removal of RY2, RR195, and RB19, respectively.

Table S4

Time (hr)	Type of Dye	0.5 g	1 g	1.5 g	2 g	2.5 g	F (p)	LSD
0	RY2 (λmax = 404 nm)	0.00 ± 0.00^{a}	0.00 ± 0.00^{b}	0.00 ± 0.00^{c}	0.00 ± 0.00^{d}	0.00 ± 0.00^{e}	1.024 (0.639)	0.25
2	RY2 (λmax = 404 nm)	22.09 ± 0.22^{e}	31.12 ± 0.31^{d}	31.83 ± 0.32^{c}	35.63 ± 0.36^{b}	39.43 ± 0.39^{a}	4.350* (0.010)	0.30
4	RY2 (λmax = 404 nm)	38.72 ± 0.39^{e}	52.97 ± 0.53^{d}	57.72 ± 0.58^{a}	52.97 ± 0.53^{b}	52.97 ± 0.53^{c}	3.650* (0.027)	0.32
6	RY2 (λmax	44.89 ± 0.45^{e}	56.77 ± 0.57^{d}	59.38 ± 0.59^{a}	57.48 ± 0.57^{b}	54.16 ± 0.54^{c}	4.350* (0.010)	0.34

	= 404 nm)							
8	RY2 (λmax = 404 nm)	45.00 ± 0.45^{e}	57.00 ± 0.57^{d}	60.10 ± 0.60^{a}	61.00 ± 0.61^{b}	55.00 ± 0.55^{c}	4.210* (0.012)	0.36
0	RR195 (λmax = 540 nm)	0.00 ± 0.00^{a}	0.00 ± 0.00^{b}	0.00 ± 0.00^{c}	0.00 ± 0.00^{d}	0.00 ± 0.00^{e}	1.131 (0.552)	0.25
2	RR195 (λmax = 540 nm)	29.63 ± 0.30^{e}	42.86 ± 0.43^{d}	57.14 ± 0.57^{c}	62.96 ± 0.63^{b}	65.08 ± 0.65^{a}	4.510* (0.009)	0.30
4	RR195 (λmax = 540 nm)	47.09 ± 0.47^{e}	68.78 ± 0.69^{d}	72.49 ± 0.72^{c}	66.67 ± 0.67^{b}	83.60 ± 0.84^{a}	4.510* (0.009)	0.32
6	RR195 (λmax = 540 nm)	47.09 ± 0.47^{e}	69.00 ± 0.69^{d}	73.00 ± 0.73^{c}	78.84 ± 0.79^{b}	84.13 ± 0.84^{a}	4.510* (0.009)	0.34
8	RR195 (λmax = 540 nm)	47.30 ± 0.47^{e}	69.30 ± 0.69^{d}	73.80 ± 0.74^{c}	78.84 ± 0.79^{b}	85.00 ± 0.85^{a}	4.510* (0.009)	0.36
0	RB19 (λmax = 594 nm)	0.00 ± 0.00^{a}	0.00 ± 0.00^{b}	0.00 ± 0.00^{c}	0.00 ± 0.00^{d}	0.00 ± 0.00^{e}	1.175 (0.547)	0.25

2	RB19 (λmax = 594 nm)	30.58 ± 0.31^{e}	50.72 ± 0.51^{d}	64.39 ± 0.64^{c}	69.42 ± 0.69^{b}	78.42 ± 0.78^{a}	4.510* (0.009)	0.30
4	RB19 (λmax = 594 nm)	51.80 ± 0.52^{e}	78.06 ± 0.78^{d}	84.89 ± 0.85^{c}	89.21 ± 0.89^{b}	91.73 ± 0.92^{a}	4.350* (0.010)	0.32
6	RB19 (λmax = 594 nm)	53.24 ± 0.53^{e}	78.78 ± 0.79^{d}	85.61 ± 0.86^{c}	91.37 ± 0.91^{b}	92.81 ± 0.93^{a}	4.510* (0.009)	0.35
8	RB19 (λmax = 594 nm)	54.32 ± 0.54^{e}	79.00 ± 0.79^{d}	85.90 ± 0.86^{c}	92.00 ± 0.92^{b}	93.70 ± 0.94^{a}	4.510* (0.009)	0.36

Table S5

Time (hr)	Type of Dye	0.5 g	1 g	1.5 g	2 g	2.5 g	F (p)	LSD
0	RY2 (λmax = 404 nm)	0.00 ± 0.00^{a}	0.00 ± 0.00^{b}	0.00 ± 0.00^{c}	0.00 ± 0.00^{d}	0.00 ± 0.00^{e}	1.200 (0.215)	0.25
2	RY2 (λmax = 404 nm)	19.71 ± 0.20^{d}	20.67 ± 0.21^{c}	17.34 ± 0.17^{e}	15.44 ± 0.15^{f}	22.57 ± 0.23^{a}	4.280* (0.012)	0.30
4	RY2 (λmax = 404	35.63 ± 0.36^{b}	37.05 ± 0.37^{a}	34.92 ± 0.35^{c}	31.83 ± 0.32^{e}	36.58 ± 0.36^{d}	4.210* (0.013)	0.32

	nm)							
6	RY2 (λmax = 404 nm)	40.38 ± 0.40[b]	43.23 ± 0.43[a]	38.95 ± 0.39[c]	35.63 ± 0.36[e]	37.29 ± 0.37[d]	4.310* (0.011)	0.34
8	RY2 (λmax = 404 nm)	41.00 ± 0.41[b]	44.11 ± 0.44[a]	40.00 ± 0.40[c]	36.00 ± 0.36[e]	38.00 ± 0.38[d]	4.250* (0.012)	0.36
0	RR195 (λmax = 540 nm)	0.00 ± 0.00[a]	0.00 ± 0.00[b]	0.00 ± 0.00[c]	0.00 ± 0.00[d]	0.00 ± 0.00[e]	1.180 (0.225)	0.25
2	RR195 (λmax = 540 nm)	23.28 ± 0.23[e]	35.98 ± 0.36[d]	38.62 ± 0.39[c]	42.33 ± 0.42[b]	44.44 ± 0.44[a]	4.510* (0.009)	0.30
4	RR195 (λmax = 540 nm)	40.21 ± 0.40[e]	53.44 ± 0.53[d]	54.50 ± 0.55[c]	62.96 ± 0.63[b]	64.02 ± 0.64[a]	4.480* (0.009)	0.32
6	RR195 (λmax = 540 nm)	41.80 ± 0.42[e]	57.14 ± 0.57[d]	56.61 ± 0.57[c]	67.72 ± 0.68[b]	68.25 ± 0.68[a]	4.490* (0.009)	0.34
8	RR195 (λmax = 540 nm)	42.20 ± 0.42[e]	57.14 ± 0.57[d]	58.00 ± 0.58[c]	68.00 ± 0.68[b]	70.00 ± 0.70[a]	4.470* (0.009)	0.36
0	RB19	0.00 ±	0.00 ±	0.00 ±	0.00 ±	0.00 ±	1.210	0.25

	(λmax = 594 nm)	0.00^{a}	0.00^{b}	0.00^{c}	0.00^{d}	0.00^{e}	(0.218)	
2	RB19 (λmax = 594 nm)	28.78 ± 0.29^{e}	37.41 ± 0.37^{d}	46.04 ± 0.46^{c}	50.00 ± 0.50^{b}	57.91 ± 0.58^{a}	4.520* (0.009)	0.30
4	RB19 (λmax = 594 nm)	48.92 ± 0.49^{e}	59.35 ± 0.59^{d}	74.10 ± 0.74^{c}	74.82 ± 0.75^{b}	76.98 ± 0.77^{a}	4.495* (0.010)	0.32
6	RB19 (λmax = 594 nm)	52.88 ± 0.53^{e}	66.55 ± 0.67^{d}	79.86 ± 0.80^{c}	82.01 ± 0.82^{b}	82.73 ± 0.83^{a}	4.505* (0.009)	0.34
8	RB19 (λmax = 594 nm)	53.00 ± 0.53^{e}	66.90 ± 0.67^{d}	81.29 ± 0.81^{c}	83.00 ± 0.83^{b}	84.00 ± 0.84^{a}	4.490* (0.009)	0.36

Table S6

Figure 3. Effect of Different dried algal biomass of both Ulva fasciata (a,b,c) and Pterocladia capillacea (d,e,f) on the removal of RY2, RR195, and RB19, respectively.

Figure 3. Effect of Different dried algal biomass of both Ulva fasciata (a,b,c) and Pterocladia capillacea (d,e,f) on the removal of RY2, RR195, and RB19, respectively.

Table S7

Ti me	Typ e of	Initial Dye Concentra	Initial Dye Concentra	Initial Dye Concentra	Initial Dye Concentra	Initial Dye Concentra	ANO VA	Statisti cally

(hr)	Dye	tions	tions	tions	tions	tions	p-value	Significant (P < 0.05)
Time (hr)	Type of Dye	30 mg/L	60 mg/L	90 mg/L	120 mg/L	150 mg/L	ANOVA p-value	Statistically Significant (P < 0.05)
0	RY2 (λmax = 404 nm)	0.00 ± 0.00[a]	0.00 ± 0.00[a]	0.00 ± 0.00[a]	0.00 ± 0.00[a]	0.00 ± 0.00[a]	1.200 (0.215)	0.00
2	RY2 (λmax = 404 nm)	35.99 ± 0.36[a]	25.93 ± 0.26[b]	24.08 ± 0.24[c]	22.43 ± 0.22[d]	18.39 ± 0.18[e]	4.350* (0.010)	0.30
4	RY2 (λmax = 404 nm)	51.21 ± 0.51[a]	43.16 ± 0.43[b]	36.12 ± 0.36[c]	29.71 ± 0.30[d]	27.98 ± 0.28[e]	4.282* (0.011)	0.32
6	RY2 (λmax = 404 nm)	59.17 ± 0.59[a]	50.80 ± 0.51[b]	41.54 ± 0.42[c]	36.15 ± 0.36[d]	30.26 ± 0.30[e]	4.350* (0.010)	0.34
8	RY2 (λmax = 404	66.44 ± 0.66[a]	58.08 ± 0.58[b]	47.07 ± 0.47[c]	42.09 ± 0.42[d]	35.87 ± 0.36[e]	4.210* (0.012)	0.36

	nm)							
		Initial Dye Concentrations	Initial Dye Concentrations	Initial Dye Concentrations	Initial Dye Concentrations	Initial Dye Concentrations		
		10 mg/L	20 mg/L	30 mg/L	40 mg/L	50 mg/L		
0	RR195 (λmax = 540 nm)	0.00 ± 0.00[a]	0.00 ± 0.00[a]	0.00 ± 0.00[a]	0.00 ± 0.00[a]	0.00 ± 0.00[a]	1.100 (0.552)	0.25
2	RR195 (λmax = 540 nm)	29.82 ± 0.30[a]	17.92 ± 0.18[b]	4.76 ± 0.05[e]	11.42 ± 0.11[c]	4.23 ± 0.04[d]	4.600* (0.009)	0.28
4	RR195 (λmax = 540 nm)	36.14 ± 0.36[a]	20.72 ± 0.21[b]	7.31 ± 0.07[e]	14.42 ± 0.14[c]	10.41 ± 0.10[d]	4.700* (0.009)	0.30
6	RR195 (λmax = 540 nm)	41.49 ± 0.41[a]	26.70 ± 0.27[b]	16.96 ± 0.17[d]	20.35 ± 0.20[c]	12.01 ± 0.12[e]	4.750* (0.009)	0.32
8	RR195 (λmax =	44.25 ± 0.44[a]	31.31 ± 0.31[b]	24.84 ± 0.25[c]	20.58 ± 0.21[d]	14.84 ± 0.15[e]	4.800* (0.009)	0.34

	540 nm)							
		Initial Dye Concentrations 20 mg/L	Initial Dye Concentrations 40 mg/L	Initial Dye Concentrations 60 mg/L	Initial Dye Concentrations 80 mg/L	Initial Dye Concentrations 100 mg/L		
0	RB19 (λmax = 594 nm)	0.00 ± 0.00^{a}	0.00 ± 0.00^{a}	0.00 ± 0.00^{a}	0.00 ± 0.00^{a}	0.00 ± 0.00^{a}	1.150 (0.547)	0.25
2	RB19 (λmax = 594 nm)	50.57 ± 0.50^{b}	65.63 ± 0.65^{a}	67.32 ± 0.67^{a}	15.74 ± 0.16^{d}	12.89 ± 0.13^{e}	4.720* (0.009)	0.28
4	RB19 (λmax = 594 nm)	63.64 ± 0.64^{b}	71.25 ± 0.71^{a}	73.90 ± 0.74^{a}	19.84 ± 0.20^{d}	15.04 ± 0.15^{e}	4.800* (0.010)	0.30
6	RB19 (λmax = 594 nm)	63.64 ± 0.64^{b}	71.88 ± 0.72^{a}	74.15 ± 0.74^{a}	24.05 ± 0.24^{d}	20.66 ± 0.21^{c}	4.850* (0.009)	0.32
8	RB19 (λm	76.70 ± 0.77^{b}	76.59 ± 0.77^{b}	75.63 ± 0.76^{b}	25.12 ± 0.25^{c}	23.55 ± 0.24^{c}	4.900* (0.009)	0.34

ax = 594 nm)

Table S8

Time (hr)	Type of Dye	Initial Dye Concentrations	Initial Dye Concentrations	Initial Dye Concentrations	Initial Dye Concentrations	Initial Dye Concentrations	ANOVA p-value	Statistically Significant (P < 0.05)
Time (hr)	Type of Dye	30 mg/L	60 mg/L	90 mg/L	120 mg/L	150 mg/L	ANOVA p-value	Statistically Significant (P < 0.05)
0	RY2 (λmax = 404 nm)	0.00 ± 0.00^{a}	0.00 ± 0.00^{a}	0.00 ± 0.00^{a}	0.00 ± 0.00^{a}	0.00 ± 0.00^{a}	1.200 (0.639)	0.00
2	RY2 (λmax = 404 nm)	11.95 ± 0.12^{b}	15.56 ± 0.16^{a}	14.19 ± 0.14^{ab}	4.28 ± 0.04^{e}	10.03 ± 0.10^{c}	4.400* (0.010)	0.28
4	RY2 (λmax = 404 nm)	25.26 ± 0.25^{b}	29.58 ± 0.30^{a}	23.61 ± 0.24^{c}	25.16 ± 0.25^{b}	19.03 ± 0.19^{d}	4.500* (0.011)	0.30
6	RY2 (λmax =	35.49 ± 0.35^{b}	40.52 ± 0.41^{a}	30.42 ± 0.30^{c}	32.73 ± 0.33^{c}	25.70 ± 0.26^{d}	4.600* (0.010)	0.32

	404 nm)							
8	RY2 (λmax = 404 nm)	53.78 ± 0.54[a]	48.50 ± 0.49[b]	37.88 ± 0.38[c]	37.12 ± 0.37[c]	36.31 ± 0.36[c]	4.700* (0.009)	0.34
		Initial Dye Concentrations	Initial Dye Concentrations	Initial Dye Concentrations	Initial Dye Concentrations	Initial Dye Concentrations		
		10 mg/L	20 mg/L	30 mg/L	40 mg/L	50 mg/L		
0	RR195 (λmax = 540 nm)	0.00 ± 0.00a	0.00 ± 0.00a	0.00 ± 0.00a	0.00 ± 0.00a	0.00 ± 0.00a	–	-
2	RR195 (λmax = 540 nm)	32.47 ± 2.15a	33.87 ± 2.40a	10.29 ± 0.82b	20.81 ± 1.55ab	19.80 ± 1.32ab	0.004 **	2.42
4	RR195 (λmax = 540 nm)	46.52 ± 3.25a	42.79 ± 2.98a	23.33 ± 1.86b	28.44 ± 2.05ab	26.13 ± 1.74ab	0.006 **	3.41
6	RR195 (λmax =	57.16 ± 3.91a	48.71 ± 3.42a	34.15 ± 2.33b	35.22 ± 2.28ab	31.01 ± 2.02b	0.002 **	3.81

	540 nm)						
8	RR195 (λmax = 540 nm)	73.67 ± 5.02a	59.65 ± 4.11a	43.74 ± 3.12b	42.62 ± 2.87ab	40.26 ± 2.64b	0.001 **	5.39
		Initial Dye Concentrations	Initial Dye Concentrations	Initial Dye Concentrations	Initial Dye Concentrations	Initial Dye Concentrations		
		20 mg/L	40 mg/L	60 mg/L	80 mg/L	100 mg/L		
0	RB19 (λmax = 594 nm)	0.00 ± 0.00[a]	0.00 ± 0.00[a]	0.00 ± 0.00[a]	0.00 ± 0.00[a]	0.00 ± 0.00[a]	0.959	0.00
2	RB19 (λmax = 594 nm)	27.45 ± 1.37[a]	69.72 ± 3.49[b]	71.18 ± 3.56[b]	68.90 ± 3.45[b]	65.56 ± 3.28[b]	0.025*	5.12
4	RB19 (λmax = 594 nm)	47.06 ± 2.35[a]	75.84 ± 3.79[b]	79.56 ± 3.98[b]	75.20 ± 3.76[b]	78.89 ± 3.94[b]	0.013*	5.58
6	RB19 (λm	47.06 ± 2.35[a]	81.96 ± 4.10[b]	78.08 ± 3.90[b]	77.56 ± 3.88[b]	78.70 ± 3.94[b]	0.021*	5.75

	ax = 594 nm)							
8	RB19 (λmax = 594 nm)	56.86 ± 2.84[a]	81.96 ± 4.10[b]	80.05 ± 4.00[b]	80.91 ± 4.05[b]	80.37 ± 4.02[b]	0.038*	6.02

Table S9

Time (hr)	Type of Dye	Initial Dye Concentrations	Initial Dye Concentrations	Initial Dye Concentrations	Initial Dye Concentrations	Initial Dye Concentrations	ANOVA p-value	Statistically Significant (P < 0.05)
Time (hr)	Type of Dye	30 mg/L	60 mg/L	90 mg/L	120 mg/L	150 mg/L	ANOVA p-value	Statistically Significant (P < 0.05)
0	RY2 (λmax = 404 nm)	0.00 ± 0.00[a]	0.00 ± 0.00[a]	0.00 ± 0.00[a]	0.00 ± 0.00[a]	0.00 ± 0.00[a]	0.246	0.00
2	RY2 (λmax = 404 nm)	17.46 ± 0.87[a]	18.08 ± 0.90[a]	20.36 ± 1.02[a]	17.33 ± 0.87[a]	15.76 ± 0.79[a]	0.031*	1.65
4	RY2 (λm	32.84 ± 1.64[a]	31.68 ± 1.58[a]	35.63 ± 1.78[a]	30.18 ± 1.51[a]	27.54 ± 1.38[a]	0.014*	2.35

	ax = 404 nm)							
6	RY2 (λmax = 404 nm)	41.42 ± 2.07^{a}	40.80 ± 2.04^{a}	44.75 ± 2.24^{a}	38.81 ± 1.94^{a}	36.09 ± 1.80^{a}	0.012*	2.85
8	RY2 (λmax = 404 nm)	42.01 ± 2.10^{a}	45.44 ± 2.27^{a}	47.93 ± 2.40^{a}	46.68 ± 2.33^{a}	43.64 ± 2.18^{a}	0.016*	3.02
		Initial Dye Concentrations	Initial Dye Concentrations	Initial Dye Concentrations	Initial Dye Concentrations	Initial Dye Concentrations		
		10 mg/L	20 mg/L	30 mg/L	40 mg/L	50 mg/L		
0	RR195 (λmax = 540 nm)	0.00 ± 0.00^{a}	0.00 ± 0.00^{a}	0.00 ± 0.00^{a}	0.00 ± 0.00^{a}	0.00 ± 0.00^{a}	0.339	0.00
2	RR195 (λmax = 540 nm)	22.99 ± 1.15^{a}	22.56 ± 1.13^{a}	22.71 ± 1.14^{a}	22.56 ± 1.13^{a}	25.16 ± 1.26^{a}	0.050*	1.98
4	RR195 (λmax =	39.66 ± 1.98^{a}	37.88 ± 1.89^{a}	38.64 ± 1.93^{a}	38.24 ± 1.91^{a}	39.98 ± 2.00^{a}	0.045*	2.75

	540 nm)							
6	RR195 (λmax = 540 nm)	51.72 ± 2.59[a]	46.24 ± 2.31[a]	48.90 ± 2.45[a]	48.01 ± 2.40[a]	49.46 ± 2.47[a]	0.026*	3.15
8	RR195 (λmax = 540 nm)	53.45 ± 2.67[a]	55.34 ± 2.77[a]	55.13 ± 2.76[a]	53.51 ± 2.68[a]	50.97 ± 2.55[a]	0.016*	3.42
		Initial Dye Concentrations	Initial Dye Concentrations	Initial Dye Concentrations	Initial Dye Concentrations	Initial Dye Concentrations		
		20 mg/L	40 mg/L	60 mg/L	80 mg/L	100 mg/L		
0	RB19 (λmax = 594 nm)	0.00 ± 0.00[a]	0.00 ± 0.00[a]	0.00 ± 0.00[a]	0.00 ± 0.00[a]	0.00 ± 0.00[a]	0.225	0.00
2	RB19 (λmax = 594 nm)	30.70 ± 1.54[a]	26.86 ± 1.34[b]	25.12 ± 1.26[b]	24.04 ± 1.20[b]	24.42 ± 1.22[b]	0.026*	2.35
4	RB19 (λm	49.56 ± 2.48[a]	44.02 ± 2.20[b]	39.20 ± 1.96[b]	39.46 ± 1.97[b]	38.65 ± 1.93[b]	0.012*	3.15

	ax = 594 nm)							
6	RB19 (λmax = 594 nm)	60.09 ± 3.00^{a}	54.18 ± 2.71^{b}	51.36 ± 2.57^{b}	51.17 ± 2.56^{b}	49.55 ± 2.48^{b}	0.016*	3.85
8	RB19 (λmax = 594 nm)	65.35 ± 3.27^{a}	60.95 ± 3.05^{b}	59.36 ± 2.97^{b}	57.83 ± 2.89^{b}	56.81 ± 2.84^{b}	0.017*	4.12

Table S10

Time (hr)	Type of Dye	Initial Dye Concentrations	Initial Dye Concentrations	Initial Dye Concentrations	Initial Dye Concentrations	Initial Dye Concentrations	ANOVA p-value	Statistically Significant (P < 0.05)
Time (hr)	Type of Dye	30 mg/L	60 mg/L	90 mg/L	120 mg/L	150 mg/L	ANOVA p-value	Statistically Significant (P < 0.05)
0	RY2 (λmax = 404 nm)	0.00 ± 0.00^{a}	0.00 ± 0.00^{a}	0.00 ± 0.00^{a}	0.00 ± 0.00^{a}	0.00 ± 0.00^{a}	0.509	0.00
2	RY2	8.67 ± 0.43^{a}	12.72 ± 0.64^{b}	13.14 ± 0.66^{b}	12.08 ± 0.60^{b}	11.54 ± 0.58^{b}	0.020*	0.95

	(λmax = 404 nm)							
4	RY2 (λmax = 404 nm)	22.29 ± 1.11[a]	22.73 ± 1.14[a]	22.01 ± 1.10[a]	19.39 ± 0.97[b]	19.21 ± 0.96[b]	0.033*	1.55
6	RY2 (λmax = 404 nm)	32.20 ± 1.61[a]	28.14 ± 1.41[b]	26.92 ± 1.35[b]	24.65 ± 1.23[b]	23.29 ± 1.16[b]	0.010*	2.10
8	RY2 (λmax = 404 nm)	35.29 ± 1.76[a]	33.39 ± 1.67[a]	29.17 ± 1.46[b]	28.18 ± 1.41[b]	26.21 ± 1.31[b]	0.010*	2.25
		Initial Dye Concentrations	Initial Dye Concentrations	Initial Dye Concentrations	Initial Dye Concentrations	Initial Dye Concentrations		
		10 mg/L	20 mg/L	30 mg/L	40 mg/L	50 mg/L		
0	RR195 (λmax = 540 nm)	0.00 ± 0.00[a]	0.00 ± 0.00[a]	0.00 ± 0.00[a]	0.00 ± 0.00[a]	0.00 ± 0.00[a]	0.514	0.00
2	RR195 (λmax =	22.40 ± 1.12[a]	19.46 ± 0.97[b]	17.27 ± 0.86[b]	16.76 ± 0.84[b]	14.07 ± 0.70[b]	0.009*	1.55

	540 nm)							
4	RR195 (λmax = 540 nm)	32.79 ± 1.64[a]	27.03 ± 1.35[b]	26.80 ± 1.34[b]	26.37 ± 1.32[b]	21.87 ± 1.09[b]	0.017*	2.10
6	RR195 (λmax = 540 nm)	42.08 ± 2.10[a]	33.78 ± 1.69[b]	33.45 ± 1.67[b]	33.38 ± 1.67[b]	27.47 ± 1.37[b]	0.024*	2.65
8	RR195 (λmax = 540 nm)	46.45 ± 2.32[a]	39.42 ± 1.97[b]	37.95 ± 1.90[b]	33.78 ± 1.69[b]	32.20 ± 1.61[b]	0.011*	2.85
		Initial Dye Concentrations	Initial Dye Concentrations	Initial Dye Concentrations	Initial Dye Concentrations	Initial Dye Concentrations		
		20 mg/L	40 mg/L	60 mg/L	80 mg/L	100 mg/L		
0	RB19 (λmax = 594 nm)	0.00 ± 0.00[a]	0.00 ± 0.00[a]	0.00 ± 0.00[a]	0.00 ± 0.00[a]	0.00 ± 0.00[a]	0.504	0.00
2	RB19 (λm	18.96 ± 0.95[a]	23.43 ± 1.17[b]	23.03 ± 1.15[b]	23.29 ± 1.16[b]	21.01 ± 1.05[b]	0.021*	1.75

	ax = 594 nm)							
4	RB19 (λmax = 594 nm)	31.28 ± 1.56^{a}	36.43 ± 1.82^{b}	36.44 ± 1.82^{b}	35.98 ± 1.80^{b}	32.32 ± 1.62^{a}	0.037*	2.45
6	RB19 (λmax = 594 nm)	44.55 ± 2.23^{a}	46.87 ± 2.34^{a}	45.11 ± 2.26^{a}	42.56 ± 2.13^{a}	37.37 ± 1.87^{b}	0.010*	2.95
8	RB19 (λmax = 594 nm)	51.18 ± 2.56^{a}	52.90 ± 2.65^{a}	52.84 ± 2.64^{a}	45.85 ± 2.29^{b}	42.73 ± 2.14^{b}	0.015*	3.25

Table S11

Figure 5. Effect of Different Initial Dye Concentrations on the Removal of RY2, RR195, and RB19 by fresh Ulva fasciata (a,b,c) and Pterocladia capillacea (d,e,f)

Figure 5. Effect of Different Initial Dye Concentrations on the Removal of RY2, RR195, and RB19 by fresh Ulva fasciata (a,b,c) and Pterocladia capillacea (d,e,f)

Table S12

Figure 6. Effect of Different Initial Dye Concentrations on the Removal of RY2, RR195, and RB19 by dried Ulva fasciata (a,b,c) and Pterocladia capillacea (d,e,f)

Figure 6. Effect of Different Initial Dye Concentrations on the Removal of RY2, RR195, and RB19 by dried Ulva fasciata (a,b,c) and Pterocladia capillacea (d,e,f)

Table S13

Time (hr)	Type of Dye	pH 2	pH 4	pH 6	pH 8	pH 10	ANOVA p-value	Statistically Significant (P < 0.05)
0	RY2 (λmax = 404 nm)	0.00 ± 0.00[a]	0.00 ± 0.00[a]	0.00 ± 0.00[a]	0.00 ± 0.00[a]	0.00 ± 0.00[a]	0.252	0.00
2	RY2 (λmax = 404 nm)	40.79 ± 2.04[a]	44.00 ± 2.20[a]	62.07 ± 3.10[b]	58.61 ± 2.93[b]	50.14 ± 2.51[a]	0.009*	4.25
4	RY2 (λmax = 404 nm)	51.75 ± 2.59[a]	59.48 ± 2.97[a]	65.23 ± 3.26[b]	63.61 ± 3.18[b]	51.29 ± 2.56[a]	0.016*	4.75
6	RY2 (λmax = 404 nm)	57.89 ± 2.89[a]	60.06 ± 3.00[a]	67.53 ± 3.38[b]	65.83 ± 3.29[b]	62.18 ± 3.11[a]	0.009*	5.10
8	RY2 (λmax = 404 nm)	61.84 ± 3.09[a]	63.56 ± 3.18[a]	70.00 ± 3.50[b]	66.00 ± 3.30[a]	63.61 ± 3.18[a]	0.014*	5.35
0		0.00 ±	0.00 ±	0.00 ±	0.00 ±	0.00 ±	0.575	0.00

	RR195 (λmax = 540 nm)	0.00[a]	0.00[a]	0.00[a]	0.00[a]	0.00[a]		
2	RR195 (λmax = 540 nm)	79.78 ± 3.99[a]	35.16 ± 1.76[b]	32.08 ± 1.60[b]	30.97 ± 1.55[b]	28.67 ± 1.43[b]	0.012*	5.10
4	RR195 (λmax = 540 nm)	94.38 ± 4.72[a]	39.84 ± 1.99[b]	33.00 ± 1.65[b]	31.61 ± 1.58[b]	28.67 ± 1.43[b]	0.012*	5.85
6	RR195 (λmax = 540 nm)	96.63 ± 4.83[a]	45.00 ± 2.25[b]	35.11 ± 1.76[b]	31.90 ± 1.60[b]	30.67 ± 1.53[b]	0.009*	6.20
8	RR195 (λmax = 540 nm)	100.00 ± 5.00[a]	50.44 ± 2.52[b]	41.00 ± 2.05[b]	35.33 ± 1.77[b]	32.00 ± 1.60[b]	0.009*	6.50
0	RB19 (λmax = 594 nm)	0.00 ± 0.00[a]	0.00 ± 0.00[a]	0.00 ± 0.00[a]	0.00 ± 0.00[a]	0.00 ± 0.00[a]	0.766	0.00
2	RB19 (λmax = 594 nm)	85.64 ± 4.28[a]	64.34 ± 3.22[b]	41.13 ± 2.06[b]	47.43 ± 2.37[b]	45.93 ± 2.30[b]	0.009*	5.75
4	RB19 (λmax = 594	93.56 ± 4.68[a]	69.23 ± 3.46[b]	55.09 ± 2.75[b]	52.17 ± 2.61[b]	58.94 ± 2.95[a]	0.009*	6.20

	nm)							
6	RB19 (λmax = 594 nm)	94.06 ± 4.70[a]	69.23 ± 3.46[b]	63.77 ± 3.19[a]	61.66 ± 3.08[a]	66.67 ± 3.33[a]	0.009*	6.50
8	RB19 (λmax = 594 nm)	94.06 ± 4.70[a]	70.00 ± 3.50[a]	64.10 ± 3.21[a]	62.00 ± 3.10[a]	67.00 ± 3.35[a]	0.009*	6.75

Table S14

Time (hr)	Type of Dye	pH 2	pH 4	pH 6	pH 8	pH 10	ANOVA p-value	Statistically Significant ($P < 0.05$)
0	RY2 (λmax = 404 nm)	0.00 ± 0.00[a]	0.00 ± 0.00[a]	0.00 ± 0.00[a]	0.00 ± 0.00[a]	0.00 ± 0.00[a]	0.938	0.00
2	RY2 (λmax = 404 nm)	65.79 ± 3.29[a]	53.64 ± 2.68[b]	51.72 ± 2.59[b]	53.89 ± 2.69[b]	48.14 ± 2.41[b]	0.014*	4.15
4	RY2 (λmax = 404 nm)	74.12 ± 3.71[a]	55.98 ± 2.80[b]	55.46 ± 2.77[b]	58.61 ± 2.93[b]	53.01 ± 2.65[b]	0.011*	4.60
6	RY2 (λmax = 404 nm)	76.32 ± 3.82[a]	60.64 ± 3.03[b]	62.07 ± 3.10[b]	67.50 ± 3.38[a]	63.32 ± 3.17[b]	0.012*	4.95
8	RY2	76.32 ±	63.85 ±	63.00 ±	70.00 ±	64.10 ±	0.024*	5.20

	(λmax = 404 nm)	3.82[a]	3.19[b]	3.15[b]	3.50[a]	3.21[b]		
0	RR195 (λmax = 540 nm)	0.00 ± 0.00[a]	0.00 ± 0.00[a]	0.00 ± 0.00[a]	0.00 ± 0.00[a]	0.00 ± 0.00[a]	0.498	0.00
2	RR195 (λmax = 540 nm)	79.78 ± 3.99[a]	16.41 ± 0.82[b]	28.30 ± 1.42[b]	30.97 ± 1.55[b]	38.00 ± 1.90[b]	0.009*	5.25
4	RR195 (λmax = 540 nm)	89.89 ± 4.49[a]	20.31 ± 1.02[b]	36.48 ± 1.82[b]	34.19 ± 1.71[b]	44.67 ± 2.23[b]	0.009*	5.90
6	RR195 (λmax = 540 nm)	93.26 ± 4.66[a]	29.69 ± 1.48[b]	40.25 ± 2.01[b]	50.67 ± 2.53[a]	47.10 ± 2.36[a]	0.009*	6.30
8	RR195 (λmax = 540 nm)	94.00 ± 4.70[a]	30.00 ± 1.50[b]	44.00 ± 2.20[a]	51.00 ± 2.55[a]	50.24 ± 2.51[a]	0.010*	6.65
0	RB19 (λmax = 594 nm)	0.00 ± 0.00[a]	0.00 ± 0.00[a]	0.00 ± 0.00[a]	0.00 ± 0.00[a]	0.00 ± 0.00[a]	0.850	0.00
2	RB19 (λmax = 594 nm)	91.09 ± 4.55[a]	56.64 ± 2.83[b]	53.96 ± 2.70[b]	49.80 ± 2.49[b]	67.07 ± 3.35[a]	0.009*	6.00

4	RB19 (λmax = 594 nm)	96.04 ± 4.80[a]	62.94 ± 3.15[b]	60.75 ± 3.04[b]	63.24 ± 3.16[b]	82.11 ± 4.11[a]	0.016*	6.50
6	RB19 (λmax = 594 nm)	97.03 ± 4.85[a]	69.23 ± 3.46[b]	69.06 ± 3.45[a]	71.54 ± 3.58[a]	88.62 ± 4.43[a]	0.012*	6.85
8	RB19 (λmax = 594 nm)	97.88 ± 4.89[a]	69.93 ± 3.50[b]	69.43 ± 3.47[a]	71.94 ± 3.60[a]	89.00 ± 4.45[a]	0.012*	7.10

Table S15

Time (hr)	Type of Dye	pH 2	pH 4	pH 6	pH 8	pH 10	ANOVA p-value	Statistically Significant (P < 0.05)
0	RY2 (λmax = 404 nm)	0.00 ± 0.00[a]	0.00 ± 0.00[a]	0.00 ± 0.00[a]	0.00 ± 0.00[a]	0.00 ± 0.00[a]	0.042	0.00
2	RY2 (λmax = 404 nm)	7.10 ± 0.36[a]	6.41 ± 0.32[a]	2.59 ± 0.13[b]	15.28 ± 0.76[a]	4.58 ± 0.23[b]	0.018*	0.85
4	RY2 (λmax = 404 nm)	30.00 ± 1.50[a]	27.70 ± 1.39[a]	16.38 ± 0.82[b]	17.22 ± 0.86[b]	8.31 ± 0.42[b]	0.011*	1.70
6	RY2 (λmax = 404	45.00 ± 2.25[a]	41.69 ± 2.08[a]	29.60 ± 1.48[b]	28.06 ± 1.40[b]	22.35 ± 1.12[b]	0.009*	2.25

	nm)							
8	RY2 (λmax = 404 nm)	57.11 ± 2.86[a]	50.73 ± 2.54[a]	39.08 ± 1.95[b]	39.17 ± 1.96[b]	34.10 ± 1.71[b]	0.012*	2.75
0	RR195 (λmax = 540 nm)	0.00 ± 0.00[a]	0.00 ± 0.00[a]	0.00 ± 0.00[a]	0.00 ± 0.00[a]	0.00 ± 0.00[a]	0.429	0.00
2	RR195 (λmax = 540 nm)	10.11 ± 0.51[a]	5.47 ± 0.27[b]	1.89 ± 0.09[b]	10.32 ± 0.52[a]	11.33 ± 0.57[a]	0.017*	0.90
4	RR195 (λmax = 540 nm)	29.21 ± 1.46[a]	27.34 ± 1.37[a]	23.27 ± 1.16[a]	27.10 ± 1.36[a]	15.33 ± 0.77[b]	0.019*	1.85
6	RR195 (λmax = 540 nm)	51.56 ± 2.58[a]	49.44 ± 2.47[a]	37.11 ± 1.86[b]	41.94 ± 2.10[b]	20.67 ± 1.03[b]	0.009*	2.50
8	RR195 (λmax = 540 nm)	60.16 ± 3.01[a]	49.44 ± 2.47[a]	45.91 ± 2.30[a]	41.94 ± 2.10[b]	32.67 ± 1.63[b]	0.009*	2.95
0	RB19 (λmax = 594 nm)	0.00 ± 0.00[a]	0.00 ± 0.00[a]	0.00 ± 0.00[a]	0.00 ± 0.00[a]	0.00 ± 0.00[a]	0.988	0.00
2	RB19	0.99 ±	10.49 ±	5.66 ±	6.81 ±	4.88 ±	0.021*	0.70

	(λmax = 594 nm)	0.05[b]	0.52[a]	0.28[b]	0.34[b]	0.24[b]		
4	RB19 (λmax = 594 nm)	34.65 ± 1.73[a]	33.57 ± 1.68[a]	24.91 ± 1.25[b]	28.51 ± 1.43[a]	19.51 ± 0.98[b]	0.012*	2.10
6	RB19 (λmax = 594 nm)	54.46 ± 2.72[a]	46.85 ± 2.34[a]	36.60 ± 1.83[b]	39.57 ± 1.98[a]	26.83 ± 1.34[b]	0.009*	2.65
8	RB19 (λmax = 594 nm)	63.37 ± 3.17[a]	54.55 ± 2.73[a]	49.36 ± 2.47[a]	44.15 ± 2.21[a]	28.46 ± 1.42[b]	0.009*	3.10

Table S16

Time (hr)	Type of Dye	pH 2	pH 4	pH 6	pH 8	pH 10	ANOVA p-value	Statistically Significant (P < 0.05)
0	RY2 (λmax = 404 nm)	0.00 ± 0.00[a]	0.00 ± 0.00[a]	0.00 ± 0.00[a]	0.00 ± 0.00[a]	0.00 ± 0.00[a]	0.742	0.00
2	RY2 (λmax = 404 nm)	25.00 ± 1.25[a]	3.50 ± 0.18[b]	1.15 ± 0.06[b]	3.33 ± 0.17[b]	2.58 ± 0.13[b]	0.035*	1.20
4	RY2 (λmax = 404 nm)	28.07 ± 1.40[a]	6.71 ± 0.34[b]	1.44 ± 0.07[b]	5.83 ± 0.29[b]	5.16 ± 0.26[b]	0.011*	1.65

6	RY2 (λmax = 404 nm)	46.05 ± 2.30[a]	21.28 ± 1.06[b]	16.38 ± 0.82[b]	15.28 ± 0.76[b]	8.02 ± 0.40[b]	0.012*	2.20
8	RY2 (λmax = 404 nm)	47.37 ± 2.37[a]	29.74 ± 1.49[b]	23.85 ± 1.19[b]	16.67 ± 0.83[b]	14.04 ± 0.70[b]	0.009*	2.50
0	RR195 (λmax = 540 nm)	0.00 ± 0.00[a]	0.00 ± 0.00[a]	0.00 ± 0.00[a]	0.00 ± 0.00[a]	0.00 ± 0.00[a]	0.223	0.00
2	RR195 (λmax = 540 nm)	42.70 ± 2.14[a]	12.58 ± 0.63[b]	6.25 ± 0.31[b]	7.10 ± 0.36[b]	3.33 ± 0.17[b]	0.012*	2.10
4	RR195 (λmax = 540 nm)	56.18 ± 2.81[a]	24.53 ± 1.23[b]	9.37 ± 0.47[b]	14.84 ± 0.74[b]	12.67 ± 0.63[b]	0.009*	2.75
6	RR195 (λmax = 540 nm)	73.03 ± 3.65[a]	35.85 ± 1.79[b]	27.34 ± 1.37[b]	23.23 ± 1.16[b]	14.00 ± 0.70[b]	0.009*	3.40
8	RR195 (λmax = 540 nm)	71.91 ± 3.60[a]	45.91 ± 2.30[a]	32.81 ± 1.64[b]	25.81 ± 1.29[b]	20.00 ± 1.00[b]	0.009*	3.65
0	RB19 (λmax	0.00 ± 0.00[a]	0.00 ± 0.00[a]	0.00 ± 0.00[a]	0.00 ± 0.00[a]	0.00 ± 0.00[a]	0.228	0.00

	= 594 nm)							
2	RB19 (λmax = 594 nm)	21.29 ± 1.06^{a}	14.34 ± 0.72^{a}	21.51 ± 1.08^{a}	24.90 ± 1.25^{a}	22.36 ± 1.12^{a}	0.017*	1.80
4	RB19 (λmax = 594 nm)	29.70 ± 1.49^{a}	17.83 ± 0.89^{b}	26.04 ± 1.30^{a}	35.18 ± 1.76^{a}	26.83 ± 1.34^{a}	0.011*	2.30
6	RB19 (λmax = 594 nm)	53.96 ± 2.70^{a}	33.57 ± 1.68^{b}	42.64 ± 2.13^{a}	51.78 ± 2.59^{a}	32.93 ± 1.65^{b}	0.012*	2.95
8	RB19 (λmax = 594 nm)	67.82 ± 3.39^{a}	45.53 ± 2.28^{b}	49.81 ± 2.49^{a}	61.26 ± 3.06^{a}	42.31 ± 2.12^{b}	0.010*	3.40

Table S17

Figure 7. Effect of Different pHs on the Removal of RY2, RR195, and RB19 by fresh Ulva fasciata (a,b,c) and Pterocladia capillacea (d,e,f)

Figure 7. Effect of Different pHs on the Removal of RY2, RR195, and RB19 by fresh Ulva fasciata (a,b,c) and Pterocladia capillacea (d,e,f)
